# s-Block cooperative catalysis: alkali metal magnesiate-catalysed cyclisation of alkynols†
†Electronic supplementary information (ESI) available: General experimental procedures, additional kinetic plots, NMR spectra of catalytic reactions, and characterization data for new compounds (PDF). See DOI: 10.1039/c9sc01598a


**DOI:** 10.1039/c9sc01598a

**Published:** 2019-05-14

**Authors:** Michael Fairley, Laia Davin, Alberto Hernán-Gómez, Joaquín García-Álvarez, Charles T. O'Hara, Eva Hevia

**Affiliations:** a WestCHEM , Department of Pure and Applied Chemistry , University of Strathclyde , Glasgow , G1 1XL , UK . Email: charlie.ohara@strath.ac.uk; b Departamento de Química Orgánica e Inorgánica , Facultad de Química , Universidad de Oviedo , E-33071 Oviedo , Spain

## Abstract

Mixed s-block metal organometallic reagents have been successfully utilised in the catalytic intramolecular hydroalkoxylation of alkynols. This success has been attributed to the unique manner in which these reagents can overcome the challenges of the reaction: namely OH activation and coordination to and then addition across a C≡C bond. In order to optimise the reaction conditions and to garner vital catalytic system requirements, a series of alkali metal magnesiates were enlisted for the catalytic intramolecular hydroalkoxylation of 4-pentynol. In a prelude to the main investigation, the homometallic magnesium dialkyl reagent MgR_2_ (where R = CH_2_SiMe_3_) was utilised. This reagent was unsuccessful in cyclising the alcohol into 2-methylenetetrahydrofuran **2a** or 5-methyl-2,3-dihydrofuran **2b**, even in the presence of multidentate Lewis donor molecules such as *N*,*N*,*N*′,*N*′′,*N*′′-pentamethyldiethylenetriamine (PMDETA). Alkali metal magnesiates M^I^MgR_3_ (when M^I^ = Li, Na or K) performed the cyclisation unsatisfactorily both in the absence/presence of *N*,*N*,*N*′,*N*′-tetramethylethylenediamine (TMEDA) or PMDETA. When higher-order magnesiates (*i.e.*, M^I^_2_MgR_4_) were employed, in general a marked increase in yield was observed for M^I^ = Na or K; however, the reactions were still sluggish with long reaction times (22–36 h). A major improvement in the catalytic activity of the magnesiates was observed when the crown ether molecule 15-crown-5 was combined with sodium magnesiate Na_2_MgR_4_(TMEDA)_2_ furnishing yields of 87% with **2a** : **2b** ratios of 95 : 5 after 5 h. Similar high yields of 88% with **2a** : **2b** ratios of 90 : 10 after 3 h were obtained combining 18-crown-6 with potassium magnesiate K_2_MgR_4_(PMDETA)_2_. Having optimised these systems, substrate scope was examined to probe the range and robustness of 18-crown-6/K_2_MgR_4_(PMDETA)_2_ as a catalyst. A wide series of alkynols, including terminal and internal alkynes which contain a variety of potentially reactive functional groups, were cyclised. In comparison to previously reported monometallic systems, bimetallic 18-crown-6/K_2_MgR_4_(PMDETA)_2_ displays enhanced reactivity towards internal alkynol-cyclisation. Kinetic studies revealed an inhibition effect of substrate on the catalysts *via* adduct formation and requiring dissociation prior to the rate limiting cyclisation step.

## Abstract 2

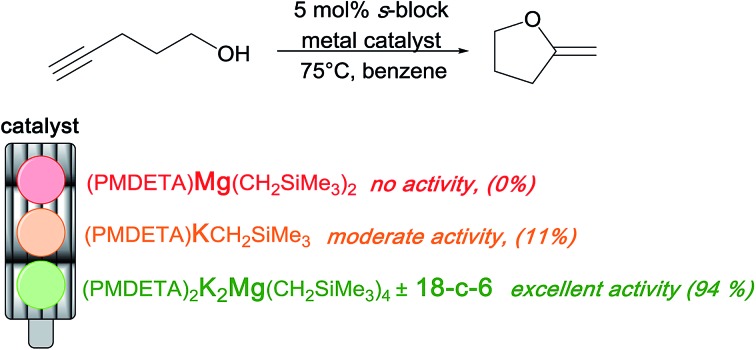
Through mixed metal cooperativity, alkali metal magnesiates efficiently catalyse the cyclisation of alkynols.

## Introduction

Since first reported by Wittig in 1951,^1^ alkali metal magnesiates have evolved from mere curiosities to a new family of versatile organometallic reagents which finds widespread applications in organic synthesis.^2^–^7^ By engaging metal–metal cooperativities, these bimetallic systems can offer superior chemo- and regioselectivities and/or functional group tolerances to those of their monometallic counterparts.^8^ Most reactivity studies have focused on using these reagents as metallating reagents (*via* Mg–H or Mg–X exchange processes) as well as anionic transfer agents to unsaturated organic molecules.^9^,^10^ Some key breakthroughs in the field which highlight the synergistic power of these bimetallic partnerships include Knochel's Turbo Grignard reagents RMgCl·LiCl (R = alkyl) which allow the functionalisation of a wide range of organic molecules *via* Mg–halogen exchange^2^ or Mulvey and O'Hara's template metallation, where mixed Na/Mg macrocyclic bases enable remote di-magnesiation of aromatic molecules.^4^,^5^ Despite the increasing interest that s-block metal catalysis is currently attracting,^9^,^11^–^30^ catalytic applications of these bimetallic systems have hardly been researched.^31^ Breaking new ground in this area, we recently reported sodium tris(alkyl)magnesiate NaMg(CH_2_SiMe_3_)_3_ 32 as an efficient precatalyst for hydroamination of a variety of carbodiimides and isocyanates.^33^,^34^ Operating synergistically, this bimetallic precatalyst displays an enhanced catalytic ability compared to those found for its homometallic components NaCH_2_SiMe_3_ and Mg(CH_2_SiMe_3_)_2_, allowing hydroaminations to take place under very mild reaction conditions (mostly at ambient temperature) in nearly quantitative yields.

Going beyond these proof of concept studies, here we investigate the catalytic ability of alkali metal magnesiates to promote intramolecular hydroalkoxylation reactions of alkynyl alcohols. Being 100% atom efficient,^35^ this is a highly desirable route to provide straightforward access to a series of synthetically significant O-heterocycles such as hydrofurans, pyrans, benzofurans, *etc.* that are fundamentally important to many natural products^36^ and pharmaceutical^37^ compounds. However, the large bond enthalpy of typical O–H bonds and the modest reactivity of electron-rich olefins with nucleophiles can make these transformations especially challenging.^38^

**Scheme 1:**
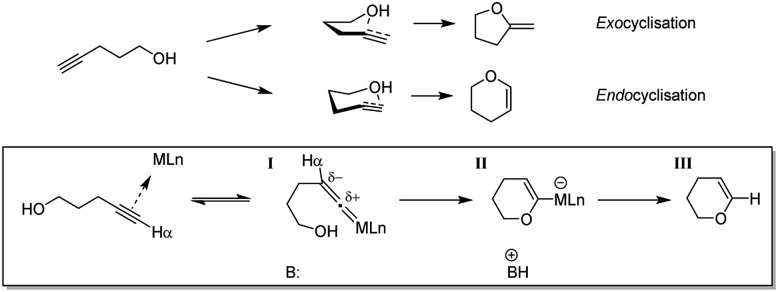
Conversion of 4-pentynol to respective five-membered exo- and six-membered endocyclised products. The latter conversion proceeds *via* vinylidene (**I**) and Fischer oxacarbene (**II**) intermediates.

A catalogue of catalysts has been developed for the cyclisation of alkynols, including various transition metal complexes as well as alkaline earth and f-block compounds. Transition metals previously used to promote this transformation are mostly precious metals^39^–^48^ but a few others such as copper,^49^ iron^50^ and tungsten^51^,^52^ have also been investigated. Different metal complexes have been found to offer different product selectivities, as there are two distinct cyclisation routes for hydroalkoxylation of alkynols (Scheme 1). For instance, tungsten, ruthenium and molybdenum complexes have been used to produce endocyclised enol ether products. This occurs due to the proposed formation of an η^2^-vinylidene metal complex intermediate (Scheme 1, **I**), which is then susceptible to nucleophilic addition to generate a Fischer oxacarbene (Scheme 1, **II**) that ultimately yields the endocyclised product (Scheme 1, **III**).^53^–^56^ However more commonly, where no electronically biased intermediate is formed, exocyclisation occurs due to the more favourable approach of the nucleophile with respect to the π-system.^57^,^58^

**Scheme 2:**
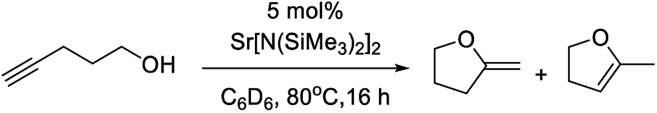
Hydroalkoxylation of 4-pentynol catalysed by heavier alkaline earth metal amides.^62^

Besides the numerous examples of transition metal catalysts, the use of f- and s-block catalysts for this reaction has been investigated. In terms of f-block chemistry, Marks has utilised lanthanide amide catalysts,^59^,^60^ while Otero and Lara-Sánchez have employed heteroscorpionate rare earth catalysts.^61^ For s-block catalysis, Hill^62^ has used calcium, strontium and barium amides (Scheme 2).

These particular studies highlight that transformations proceed selectively *via* exocyclisation, although with alkaline earth metal amides two different product isomers were observed, an internal and external alkene. Since Hill's initial study, several papers focusing on alkali metal and alkaline earth catalysts have been published. Liu has shown that potassium *tert*-butoxide is an effective and selective catalyst for cyclisation of aromatic alkynylamines and alkynols,^63^ albeit with the need for elevated temperatures and highly polar solvents. Utilising similar reaction conditions, Wang employed potassium carbonate to prepare indole/pyrrole-fused 1,4-oxazines.^64^ Baire has also recently reported that cycloisomerisation of *cis*-6-hydroxy- and *cis*-6-acyloxyhex-2-en-4-ynals to 2-acylfurans and 2-(1-acyloxyalkenyl)furans is achievable using calcium catalysis.^65^ In contrast, magnesium-based reagents have not followed suit by showing very little promise to catalyse these types of transformations; whereas the catalytic properties of s-block bimetallic complexes remain unexplored in this context.

Expanding the scope of s-block cooperative catalysis, here we report the first catalytic applications of alkali metal magnesiates to promote cyclisation reactions, focusing on intramolecular hydroalkoxylation of alkynols. Benefitting from the enhanced metallating and nucleophilic abilities of these synergistic systems, we envisaged that they could play a dual role in overcoming the main challenges encountered in these transformations, facilitating not only OH activation, but also the required addition across C≡C bonds. Combining kinetic experiments with reactivity studies, here we provide informative mechanistic insights on how cooperative effects can be maximised as well as on the key role that each metal plays in these novel magnesiate-catalysed transformations.

## Results and discussion

Intrigued by the lack of a magnesium amide-catalysed reaction from the series of alkaline earth metals investigated by Hill, our first reaction used the neutral bis(alkyl)magnesium reagent Mg(CH_2_SiMe_3_)_2_, containing thermally stable monosilyl groups, as a potential pre-catalyst. Using 4-pentynol (**1**) (as previously employed by Marks and Hill) for benchmarking with a catalytic quantity of the magnesium reagent, no reaction was observed even when the mixture was heated to 75 °C for 36 h (Table 1, entry 1).

**Table 1:** Optimisation of the catalyst for cyclisation of 4-pentynol (**1**)

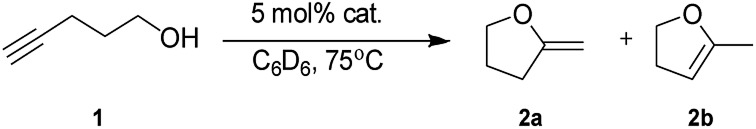				
Entry	Pre-catalyst (R = CH_2_SiMe_3_)	Time, h	Yield^*b*^ (%)	Isomer ratio (**2a** : **2b**)
1	MgR_2_^*a*^	36	0	—
2	MgR_2_ + 18-c-6 (±PMDETA)^*a*^ ^,^^*d*^	36	0	—
3	KR^*a*^	36	11	99 : 1
4	KR + 18-c-6^*c*^	16	70	93 : 7
5	LiMgR_3_^*a*^	36	3	—^*d*^
6	NaMgR_3_^*a*^	36	12	97 : 3
7	NaMgR_3_(TMEDA)^*a*^	36	13	96 : 4
8	KMgR_3_^*a*^	36	16	98 : 2
9	KMgR_3_(PMDETA)^*a*^	36	20	94 : 6
10	KMgR_3_ + 18-c-6^*a*^ ^,^^*d*^	10	77	81 : 19
11	Li_2_MgR_4_(TMEDA)_2_^*b*^	36	3	—^*e*^
12	Na_2_MgR_4_(TMEDA)_2_^*b*^	36	83	91 : 9
13	K_2_MgR_4_(PMDETA)_2_^*b*^	22	91	86 : 14
14	Na_2_MgR_4_(TMEDA)_2_ + 15-c-5^*b*^ ^,^^*d*^	5	87	95 : 5
15	K_2_MgR_4_(PMDETA)_2_ + 18-c-6^*b*^ ^,^^*d*^	3	88	90 : 10
16	K_2_MgR_4_(TMEDA)_2_ + 18-c-6^*b*^ ^,^^*d*^	3	94	90 : 10

^*a*^Reactions were performed in a Young's cap NMR tube, using 0.5 mmol substrate (4-pentynol) (**1**) and 0.025 mmol (5 mol%) pre-catalyst.

^*b*^Reactions were performed in a Young's cap NMR tube, using 0.6 mmol (1.2 eq.) substrate (4-pentynol) (**1**) and 0.025 (5 mol%) pre-catalyst. This additional 0.1 mmol (20 mol%/0.2 eq.) **1** was employed to convert the pre-catalyst to the ‘active catalyst’ (*vide infra*).

^*c*^Calculated from ^1^H NMR spectroscopic data by integration against an internal standard (10 mol% 1,2,3,4-tetraphenylnaphthalene).

^*d*^A stoichiometric quantity of crown ether co-catalyst used according to the alkali metal [*i.e.*, 10 mol% 18-c-6 for 5 mol% K_2_MgR_4_(PMDETA)_2_].

^*e*^Due to low yields obtained and the inherent error within the measurement, an isomer ratio is not reported.

This raises the question, “why is magnesium unable to mediate this cyclisation whereas calcium can?” Major contributory factors are likely due to the significant difference in ionic radii between the two metal cations and the low π-philicity of magnesium.^66^ DFT calculations were carried out by Marks investigating the quantity of alkynol molecules which could coordinate to the active lanthanide catalyst when La(HMDS)_3_ is added as a pre-catalyst.^67^ Interestingly these calculations showed that the most likely catalytically active species is a La ion coordinated to three alkynoxide ligands, binding *via* the oxide anion as well as forming a close-range π-interaction with the alkyne unit. An additional substrate molecule coordinates to the metal *via* its O atom, affording a coordinatively saturated species. Furthermore, it appears that these La–π-interactions activate the alkyne for insertion into the lanthanum–oxygen bond and additionally position the alkynoxide perfectly for cyclisation. Bearing this in mind, the lack of reactivity observed when using Mg(CH_2_SiMe_3_)_2_ could be due to the magnesium centre not being large enough to simultaneously bind to the oxide anion and be in close proximity to the π-density of the carbon–carbon triple bond.

We next focused on testing homometallic alkali metal organometallics, which, being significantly more polar than their Mg counterparts, have recently found applications in hydrophosphination catalysis.^68^ Li(CH_2_SiMe_3_) and Na(CH_2_SiMe_3_) were employed in the same reaction; however, the conversions were poor (<5%) (see ESI†). Using the heavier congener K(CH_2_SiMe_3_) (Table 1, entry 3) also results in low conversion over a 36 h period (11%). Although the conversion is again low, the fact that the reaction was more successful than those with Li(CH_2_SiMe_3_), Na(CH_2_SiMe_3_) and Mg(CH_2_SiMe_3_)_2_ can perhaps be attributed to the larger ionic radius of potassium *versus* the other metals allowing it to interact with the carbon–carbon triple bond, activating it for cyclisation.

Next, we investigated the catalytic ability of a lower-order (1 : 1, alkali metal : Mg ratio) magnesiate. It was envisaged that a bimetallic magnesiate could function cooperatively, *i.e.*, promote a dual-activation where a magnesium alkoxide forms, thus preparing one end of the substrate for cyclisation while the alkali metal could then interact with the π-system of the carbon–carbon triple bond. As alluded to previously, this latter feature is essential as the magnesium alone cannot provide this interaction. LiMg(CH_2_SiMe_3_)_3_ (Table 1, entry 5) was the first magnesiate tested but it struggled to promote the reaction, presumably again due to size limitations of the lithium centre (*i.e.*, it is sterically blocked by the ligand set). Moving to sodium (Table 1, entry 6), disappointingly, this sodium magnesiate only promoted the reaction modestly [about as effectively as K(CH_2_SiMe_3_)]. When the potassium magnesiate KMg(CH_2_SiMe_3_)_3_ (Table 1, entry 8) was employed, the yield (16%) was comparable to that observed for K(CH_2_SiMe_3_) (11%). So, in summary, lower order magnesiate reagents are generally poor at catalysing the hydroalkoxylation/cyclisation reaction.

In the hope of improving this situation, higher-order (2 : 1, alkali metal : Mg ratio) magnesiate reagents were considered. The Lewis basic proligands, TMEDA (*N*,*N*,*N*′,*N*′-tetramethylethylenediamine) and PMDETA (*N*,*N*,*N*′,*N*′′,*N*′′-pentamethyldiethylenetriamine), were employed to completely fill the coordination sphere of the alkali metals. Again, starting with the lithium derivative, the higher-order magnesiate (Table 1, entry 11) performed as poorly as the lower-order lithium magnesiate in the reaction (3%). However, when Na_2_MgR_4_(TMEDA)_2_ was employed (Table 1, entry 12), pleasingly a substantially more successful conversion of the alcohol to the cyclised product (83%) was obtained within 36 h. The reaction efficiency can be improved further when the potassium reagent K_2_MgR_4_(PMDETA)_2_ is used (91% after 22 h, Table 1, entry 13). These two higher-order magnesiates display greatly enhanced catalytic activity compared to that of homometallic K(CH_2_SiMe_3_). In addition, they continue to have a high isomer selectivity with up to 90% of the external alkene isomer being produced. The remarkable alkali metal effect observed between Li_2_MgR_4_(TMEDA) and K_2_MgR_4_(PMDETA)_2_ is even more striking when considering that both mixed-metal species are isostructural.^69^,^70^ At this juncture we reasoned that addition of TMEDA or PMDETA in these reactions influenced the marked increase in reactivity. To test whether these multidentate Lewis bases were the key drivers in increasing the yields, we tested the lower-order NaMgR_3_(TMEDA) and KMgR_3_(PMDETA) (Table 1, entries 7 and 9), but found that adding these multidentate ligands did not enhance reaction performance. With the higher-order magnesiates, the reaction rate using K_2_MgR_4_(PMDETA)_2_ is unaffected when the bidentate Lewis base TMEDA is used in place of its tridentate analogue PMDETA (Table 2, entry 16). Hence K_2_MgR_4_(TMEDA)_2_ displayed the same reaction time and selectivity as its PMDETA variant, demonstrating the reaction rate to be unaffected by the choice of Lewis base.

**Table 2:** Catalytic reactivity of K_2_MgR_4_(PMDETA)_2_ with the 18-crown-6 additive towards various structurally diverse alkynols

Entry^*a*^ ^,^^*c*^	Substrate (5 mol% cat., C_6_D_6_)	Yield^*b*^
1	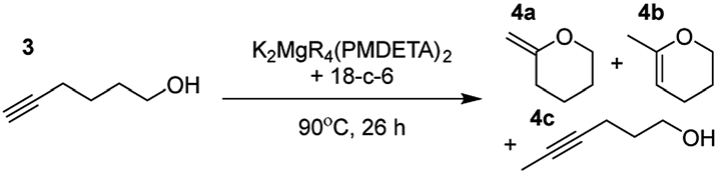	99% (14 : 9 : 77)
2	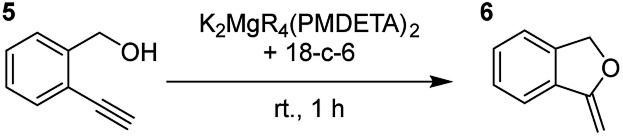	86%
3	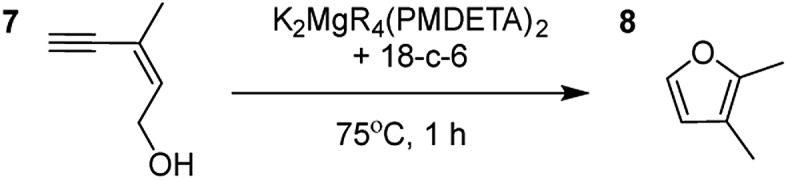	89%
4	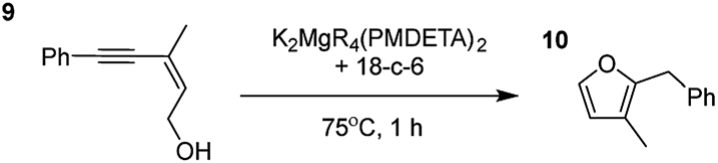	90%

^*a*^Reactions were performed in a Young's cap NMR tube, using 0.6 mmol (1.2 eq.) substrate (**3**, **5**, **7**, or **9**) and 0.025 mmol (5 mol%) pre-catalyst with 0.1 mmol (20 mol%/0.2 eq.) **1** consumed in converting the pre-catalyst to the ‘active catalyst’ (*vide infra*).

^*b*^Calculated from ^1^H NMR spectroscopic data by integration against an internal standard (10 mol% 1,2,3,4-tetraphenylnaphthalene).

^*c*^A stoichiometric quantity of crown ether co-catalyst used according to the alkali metal [*i.e.*, 10 mol% 18-c-6 for 5 mol% K_2_MgR_4_(PMDETA)_2_].

To establish whether or not both metals need to be present for the magnesiate to function, a catalytic amount of crown ether was added as a co-catalyst. As a crown ether of appropriate size is often able to sequester an alkali metal, the intention here was to ascertain, if the alkali metal was captured (non-cooperative), that the remaining anionic Mg(OR)_3_^–^ or Mg(OR)_4_^2–^ fragment, of the now solvent-separated complex, was not able to perform the catalysis on its own. Surprisingly, in contrast, the results obtained in these reactions showed a dramatic improvement in reactivity!

Adding catalytic quantities of 15-crown-5 or 18-crown-6 to the sodium and potassium systems respectively dramatically increased the activity of the magnesiate catalysts, with the sodium higher-order magnesiate now nearing completion in 5 h (Table 1, entry 14) and the potassium analogue in 3 h (Table 1, entry 15), in reactions retaining good selectivity. This reactivity enhancement was also seen with both the monometallic and lower order potassium magnesiate (Table 1, entries 4 and 10 respectively).

Due to the significant difference in completion rates of these two reactions (5 h *vs.* 3 h; Table 1, entries 14 and 15), the idea of a completely solvent-separated Mg(OR)_4_^2–^ dianion can be discounted as it would be expected that similar rates would be observed if this dianion was a catalytically active species. Although the crown ether is often expected to sequester the alkali metal there are examples of structures where the alkali metal is still able to maintain π-interactions with the anionic moiety whilst coordinated by the crown ether.^71^–^78^ Thus it appears that under these conditions, the addition of the crown ether must tune the coordination environment of K, modifying its Lewis acidity to generate a more catalytically competent species (*vide infra*). Therefore, these results suggest that the alkali metal is intimately involved and plays a key role in the cyclisation reaction. To ascertain whether the 18-crown-6 is catalytically active, when it is combined with only Mg(CH_2_SiMe_3_)_2_ the reaction fails (entry 2).

Summarising Table 1, it is clear that the organomagnesium compound used in this study is unable to catalytically promote cyclisation of alkynols. Homometallic alkali metal compounds can only poorly promote the reaction; however, when working cooperatively with a Mg compound the two metals can perform more efficiently by dual-activating the substrate. There are also clear trends that higher-order magnesiates perform better than lower-order magnesiates and that when comparing alkali metals K > Na ≫ Li presumably due to a sequential decrease in size and reduction in π-philicity. Perhaps the most surprising outcome of the optimisation study was that introducing a crown ether into higher-order magnesiates appears to greatly enhance the rate of the cyclisation, whilst maintaining a high selectivity. The reaction times and selectivity of our system (Table 1, entry 15) compare favourably with those of other catalytic systems. In terms of typical reaction times (3 h; Table 1, entry 15), this is similar to that observed by Marks (2.5 h) where only isomer **2a** is produced by employing La[N(SiMe_3_)_2_]_3_.^38^ Perhaps most pertinent to our study, Hill has employed heavy group 2 amides [Ae{N(SiMe_3_)_2_}_2_ where Ae = Ca, Sr, Ba], where reaction times ranged from 2.5–6 h, exhibiting a **2a** isomer selectivity of 90–97% (Scheme 2).^62^ Therefore to re-emphasize, our results show that by pairing a magnesium complex with a potassium one, it has been possible to overcome the severe limitations imposed by a magnesium homometallic catalyst on its own. Through cooperativity, the surprising effect was the generation of a potassium magnesiate catalyst which is even more active and equally as selective as the homometallic heavier group 2 congeners.

Having established the optimal conditions for cyclisation of 4-pentynol, the scope of the reaction was evaluated. Terminal and internal alkynes were tested to assess the range and robustness of the catalytic system (Tables 2 and 3). Interestingly, by increasing the alkynol chain length by one carbon atom (5-hexynol, **3**, Table 2, entry 1), the major product is isomerised alkyne (**4c**), where the carbon–carbon triple bond has moved from a terminal to an internal location (Table 2, entry 1). It can be envisaged that isomerisation occurs due to the angle of ring closure being unfavourable, and hence it will occur prior to a cyclisation process. Related to these findings, Tsurugi has reported that organomagnesium complexes can promote alkyne isomerisations of related compounds.^79^ Where the alkynol substrate is already held in a manner that is partially aligned to that of the cyclised product, the cyclisation progresses much more readily (Table 2, entry 2). This occurs even at ambient temperature in 1 h. Interesting selectivity is observed when it comes to cyclisation of *Z*-enynols (**7** and **9**). In Table 2 entry 3, with a terminal enynol (**7**) employing the optimised conditions we see the production of an aromatic furan product (**8**). This product differs from the product previously reported using heavier alkaline earth^59^,^60^,^80^ or lanthanide amides.^38^,^57^,^58^ With these catalytic systems the non-aromatic product, 3-methyl-2-methylene-2,5-dihydrofuran, is observed, and it is only when we move to a transition metal catalyst,^44^ we see formation of the aromatic furan product.

**Table 3:** Functional group tolerance of magnesiate-catalysed exocyclisation of internal alkynols

Entry^*a*^ ^,^^*c*^	Substrate [Ar–C≡C–(CH_2_)_3_OH]	Product(s)	Conditions [5 mol% K_2_MgR_4_(PMDETA)_2_, C_6_D_6_]	Yield (and product ratio)^*b*^
1	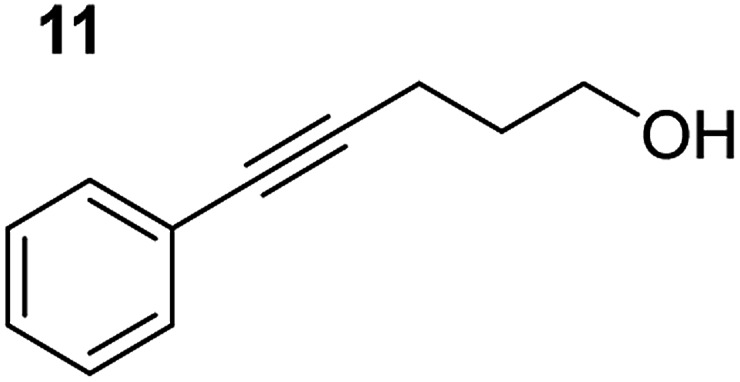	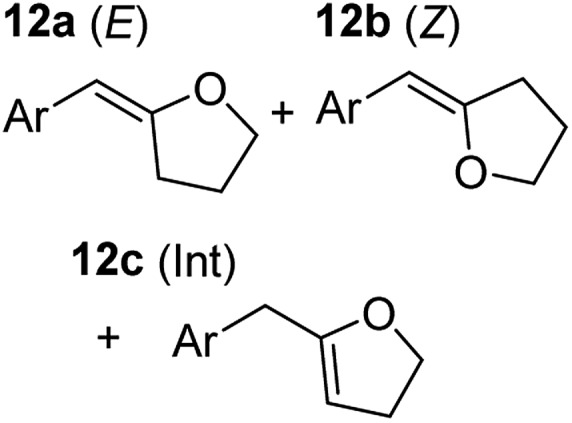	75 °C, 1 h	84% (10 : 90 : 0)
			40 °C, 36 h	6%
2	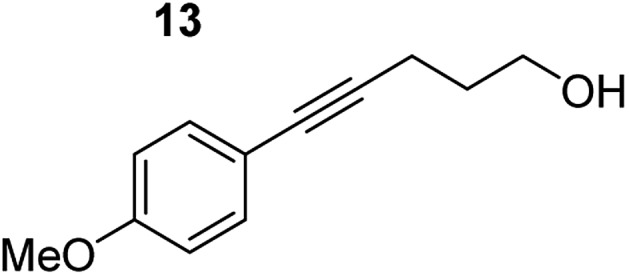	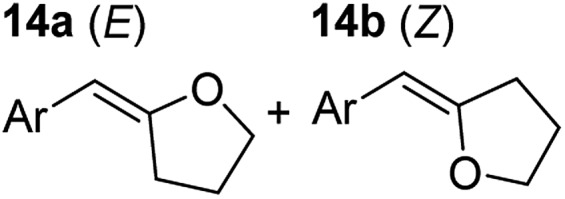	75 °C, 36 h	64% (31 : 69)
			40 °C, 36 h	1%
3	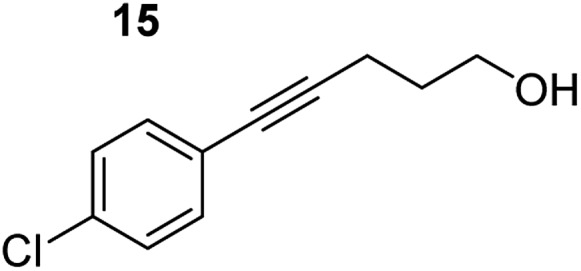	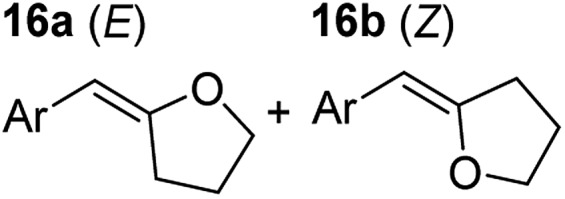	75 °C, 1 h	99% (9 : 91)
			40 °C, 36 h	51%
4	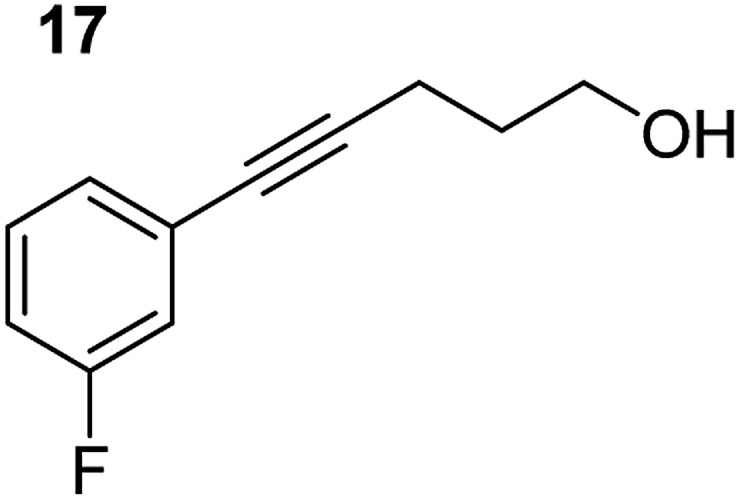	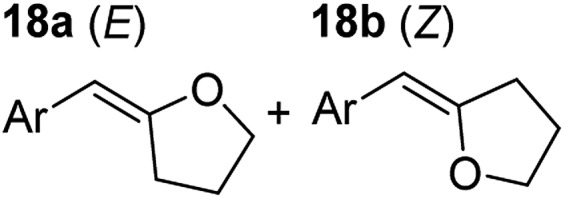	75 °C, 0.5 h	95% (8 : 92)
			40 °C, 24 h	82%
5	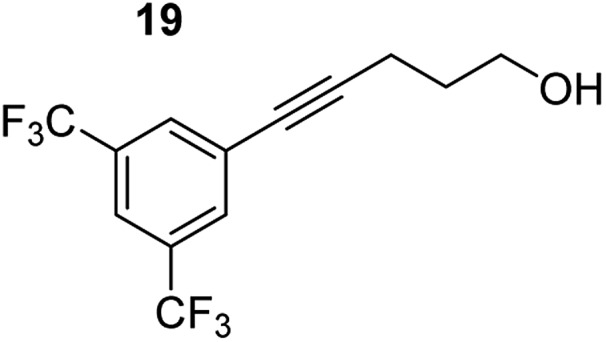	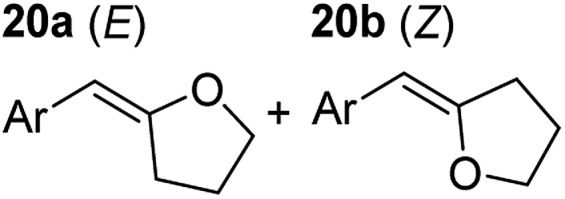	75 °C, 0.5 h	97% (4 : 96)
			40 °C, 2 h	93%
6	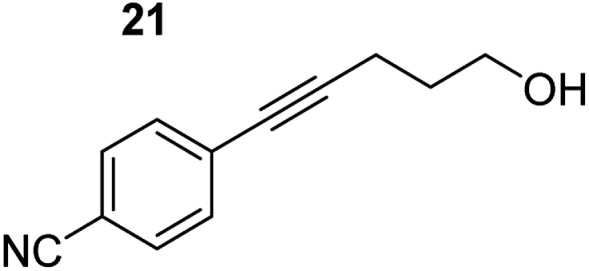	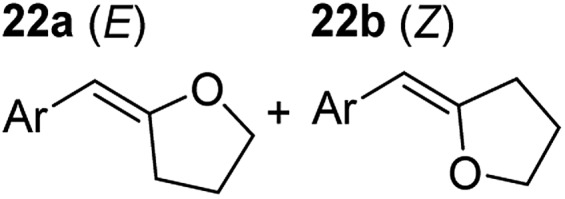	75 °C, 0.5 h	84% (6 : 94)
			40 °C, 0.5 h	76%
7	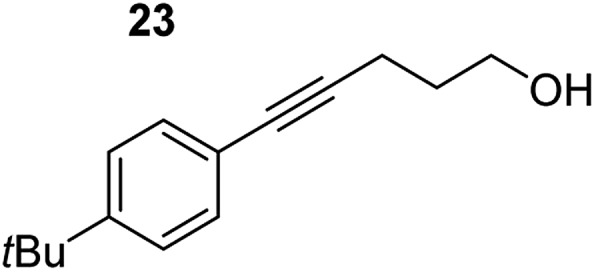	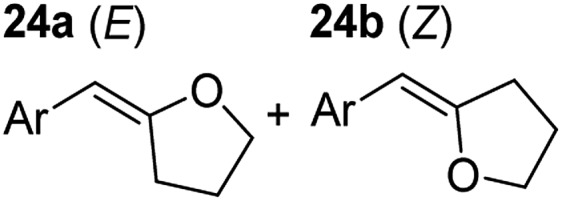	75 °C, 6 h	87% (11 : 89)
			40 °C, 36 h	2%

^*a*^Reactions were performed in a Young's cap NMR tube, using 0.5 mmol substrate (**11**, **13**, **15**, **17**, **19**, **21**, and **23**) and 0.025 mmol (5 mol%) pre-catalyst with 0.1 mmol (20 mol%/0.2 eq.) **1** consumed in converting the pre-catalyst to the ‘active catalyst’ (*vide infra*).

^*b*^Calculated from ^1^H NMR spectroscopic data by integration against an internal standard (10 mol% ferrocene).

^*c*^A stoichiometric quantity of crown ether co-catalyst used according to the alkali metal [*i.e.*, 10 mol% 18-c-6 for 5 mol% K_2_MgR_4_(PMDETA)_2_].

In order to further probe the functional group tolerance of the catalytic magnesiate system, a range of internal alkynes were synthesised with different pendant functional groups, including halogens, and electron-donating and electron-withdrawing groups. It transpired that no side reactions took place when MeO, F, Cl, CF_3_ or CN functional groups were incorporated into the substrate. Looking at 5-phenylpent-4-yn-1-ol (**11**) (Table 3, entry 1) the reaction reached completion in 1 h at 75 °C yielding only two product isomers. The major product being the external *Z*-alkene **12b** and the minor external *E* isomer **12a**. In the aforementioned studies by Hill,^62^ a mixture of three product isomers was obtained, the external alkene isomers (**12a**, **12b**) and an internal alkene (**12c**) (note these three products are still exocyclic). In this work, it was also noted that the product ratio appeared to have a degree of temperature dependence and required significantly harsher reaction conditions (90–110 °C, 16–40 h).

Despite altering the reaction temperature (reactions performed at both 40 and 75 °C), the same isomer ratios were obtained for all the reactions which tended towards completion (entries 4–6, Table 3). For entries 1–3, Table 3, the low temperature reactions (40 °C) were relatively less efficient which precluded accurate determination of the product ratio at this temperature. Focusing on the specific substrates reacting at 75 °C, when the electron donating methoxy group (entry 2, Table 3) is incorporated onto the 5-phenylpent-4-yn-1-ol scaffold, the reaction rate is dramatically reduced with only a 64% yield achieved in 36 h, compared to 1 h for the non-functionalised substrate (entry 1, Table 3). This reduction in reaction rate is also observed, albeit to a lesser extent, with the weaker donating *t*-butyl group (entry 7, Table 3). When mildly withdrawing halides are incorporated within the substrate (entries 3 and 4, Table 3) the reaction is complete in less than 1 h. At this juncture, it was deemed necessary to consider reaction times at 40 °C to ascertain whether any difference in relative rates was observed. With a Cl substituent (entry 3, Table 3), the yield was 51% after 36 h compared to 6% for the non-substituted alkynol (entry 1, Table 3). Using a F substituent, this yield can be increased to 82% in 24 h (entry 4, Table 3). Moving to stronger electron-withdrawing groups, incorporating trifluoromethyl (yield, 93%) brings the reaction time down to only 2 h at 40 °C (entry 5, Table 3), and if a cyano substituent (yield, 76%) is utilised this is further reduced to 30 min (entry 6, Table 3).

This set of substrates based on 5-phenylpent-4-yn-1-ol (**11**) show nicely the benefit of using a bimetallic system for these cyclisation reactions. With the heavier alkaline earth metal amides and lanthanide amides, substrate **11** is more challenging to cyclise, taking 16 h at 90 °C with Ca(HMDS)_2_ and about 15 h at 120 °C with La(HMDS)_2_. Marks^38^ has suggested that the reason for this sluggish reaction time with La(HMDS)_3_ is sterically driven by the phenyl substituent that prevents interaction between the metal and the internal alkyne. Using a bimetallic system, it appears that this steric clash is somewhat circumvented giving rise to a faster reaction time than the other two systems mentioned.

**Fig. 1:**
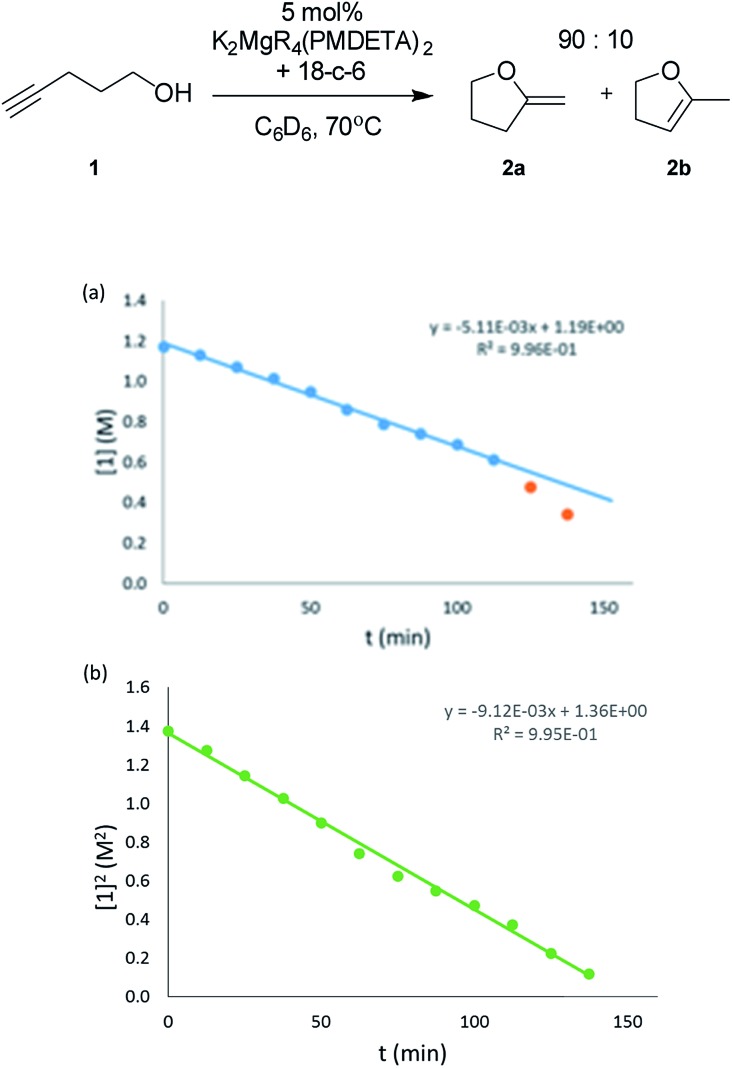
(a) Plot of the consumption of 4-pentynol (**1**) *versus* time (0.040 M cat., C_6_D_6_, 343 K). (b) Plot of the [4-pentynol (**1**)]^2^*versus* time (0.040 M cat., C_6_D_6_, 343 K).

In addition to reaction scope, kinetic studies have been carried out on the system. Prime motivations were to determine the reaction order of the alkynol substrate and catalyst present in the reaction, with the aim of providing insights into the reaction mechanism. The substrate chosen for these studies was 4-pentynol (**1**), as this was used for the initial parameterisation studies. A plot of concentration of **1** against time displays a linear fit until half conversion when a rate acceleration is observed [Fig. 1(a)].

**Scheme 3:**
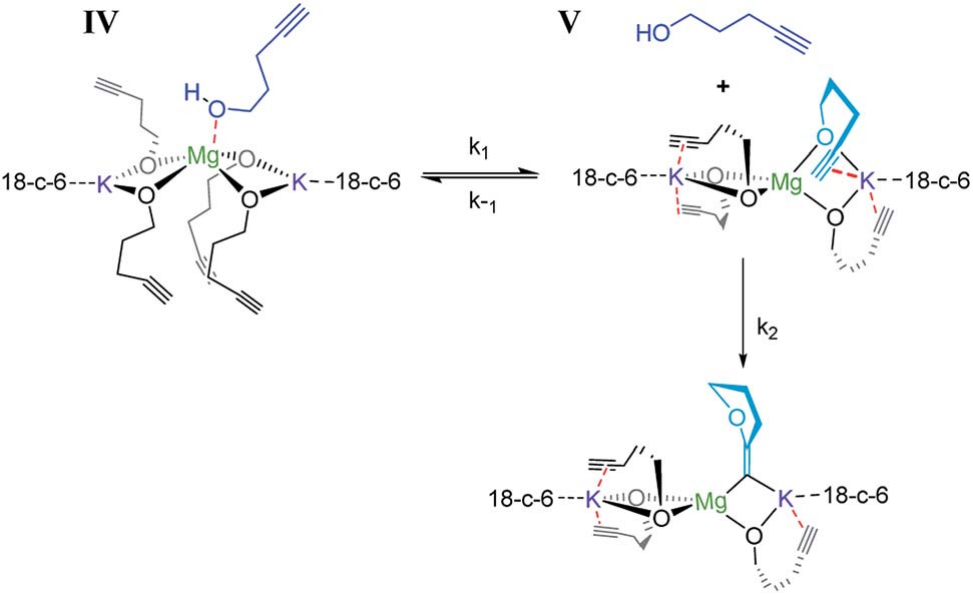
Reaction showing the coordination/decoordination of an additional alkynol molecule.

Consistent with substrate inhibition, the plot of [**1**]^2^*versus* time shows a good fit [Fig. 1(b)]. Further evidence was gathered by plotting initial reaction rates at varied alkynol concentrations (Fig. S1†), where greater rates were observed as the concentration of **1** was decreased. This negative first order was observed by Hill^62^ for the transformation employing heavier group 2 amide catalysts, where its origin is proposed to be the decoordination of a substrate molecule from the active catalyst being required before cyclisation can occur (Scheme 3).

When coordination occurs (**IV**), it appears that the cyclisation process is hindered. As the reaction is inverse first order in the substrate (*vide infra*) it is assumed that the incoming alkynol coordinates to the single Mg centre rather than the K centres, which are already highly coordinated by bonding to the crown ether. In previous studies, the addition of 18-crown-6 has had both beneficial^78^,^81^ and detrimental^77^ impacts on synthetic performance. Here we observe an improvement in catalytic activity, which could perhaps be explained by the 18-crown-6 that sufficiently satisfies the coordination environment of the metal, preventing further excess alcohol coordination from occurring which would cause inhibition.

Steady state approximation

First step slow
1

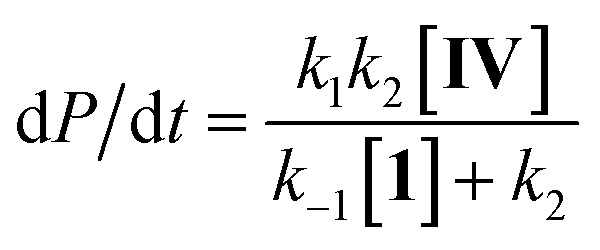

2 d*P*/d*t* = *k*_1_[**IV**]

Pre-equilibrium approximation

First step fast
3

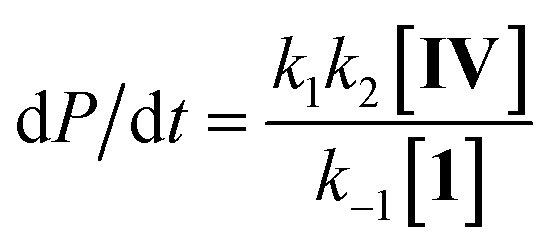




**Fig. 2:**
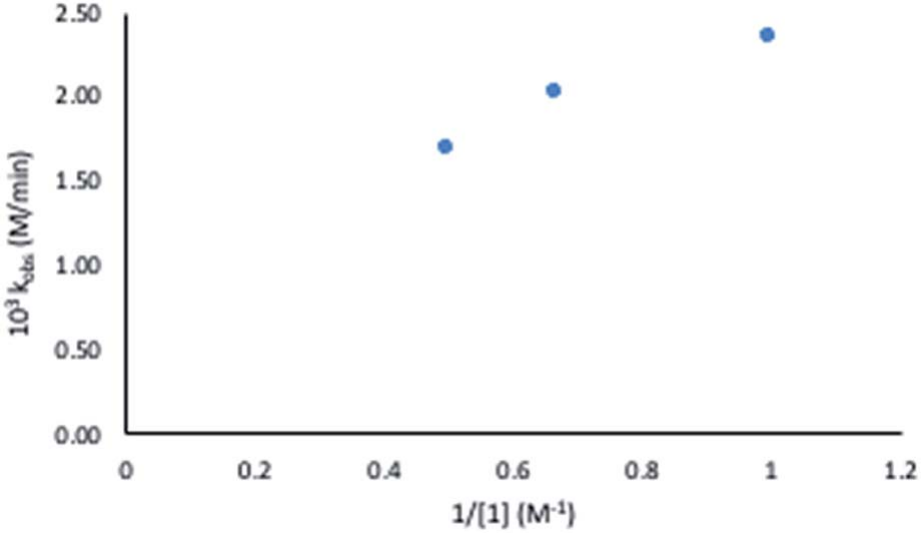
Observed rate constant at varied substrate concentrations *versus* inverse substrate concentration (**1**).

Considering this catalytic process as initial dissociation of **1** (*k*_1_ and *k*_–1_) followed by cyclisation reaction (*k*_2_) and using the steady-state approximation eqn (1) can be deduced. This equation can be simplified to eqn (2) at low concentrations of **1** (*k*_2_ ≫ *k*_–1_[**1**]), while at high concentrations of **1** (*k*_–1_[**1**] ≫ *k*_2_) eqn (3) rules this process. Alternatively, a pre-equilibrium approximation in which dissociation of **1** from magnesium does not influence the reaction rate leads exclusively to eqn (3), and hence to a good inverse first order relationship in **1** at any concentration of this ligand. The plot of the observed initial rates *vs.* 1/[**1**] (Fig. 2) reveals a linear fit with a non-zero intercept according to eqn (1), and hence consistent with a slow ligand dissociation step followed by a rapid cyclisation process.

**Fig. 3:**
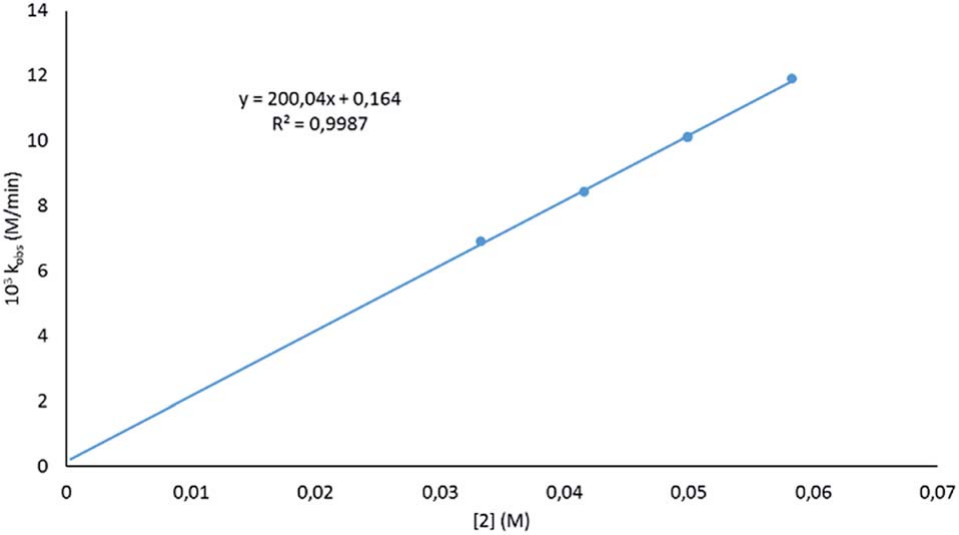
Plot of the observed rate constant at varied catalyst loadings *versus* catalyst concentration.

Looking at the catalyst order, a good fit was seen when the deduced rates for the different catalyst loadings (Fig. S2†) were plotted against the concentration of **2** (Fig. 3), indicating that the reaction has a first order dependence on the catalyst. This is similar to that found by Marks,^38^ but not the second order dependence that was found by Hill;^62^ however, in previous work by Hill using alkaline earth metal amides, a second order dependence was observed. This is said to be due to the pre-catalysts existing as dimers. The first order dependence on the magnesiate pre-catalyst observed here has also been observed in hydroamination reactions.^62^

**Fig. 4:**
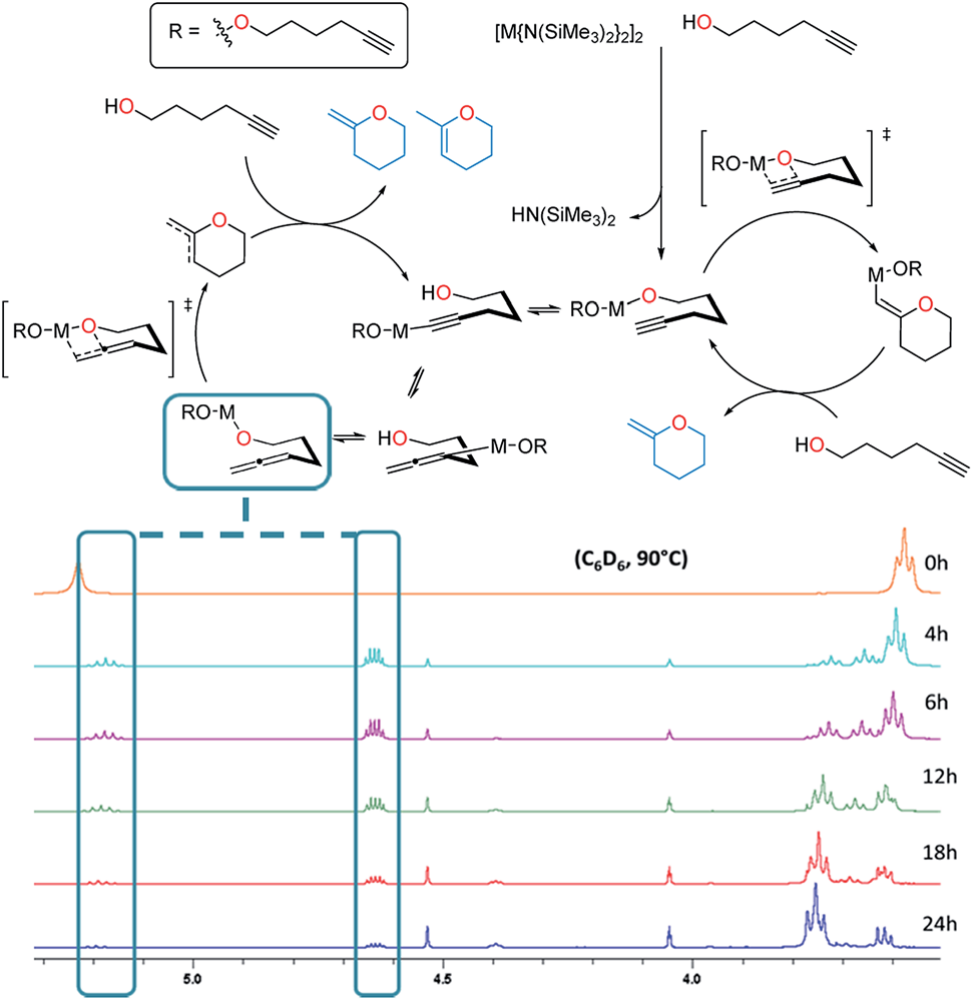
Evidence for the allene intermediate (*via*^1^H NMR spectroscopy, C_6_D_6_, 400 MHz) produced during the cyclisation of 5-hexynol (**3**) (Table 2, entry 1) as proposed by Hill.^62^

**Scheme 4:**
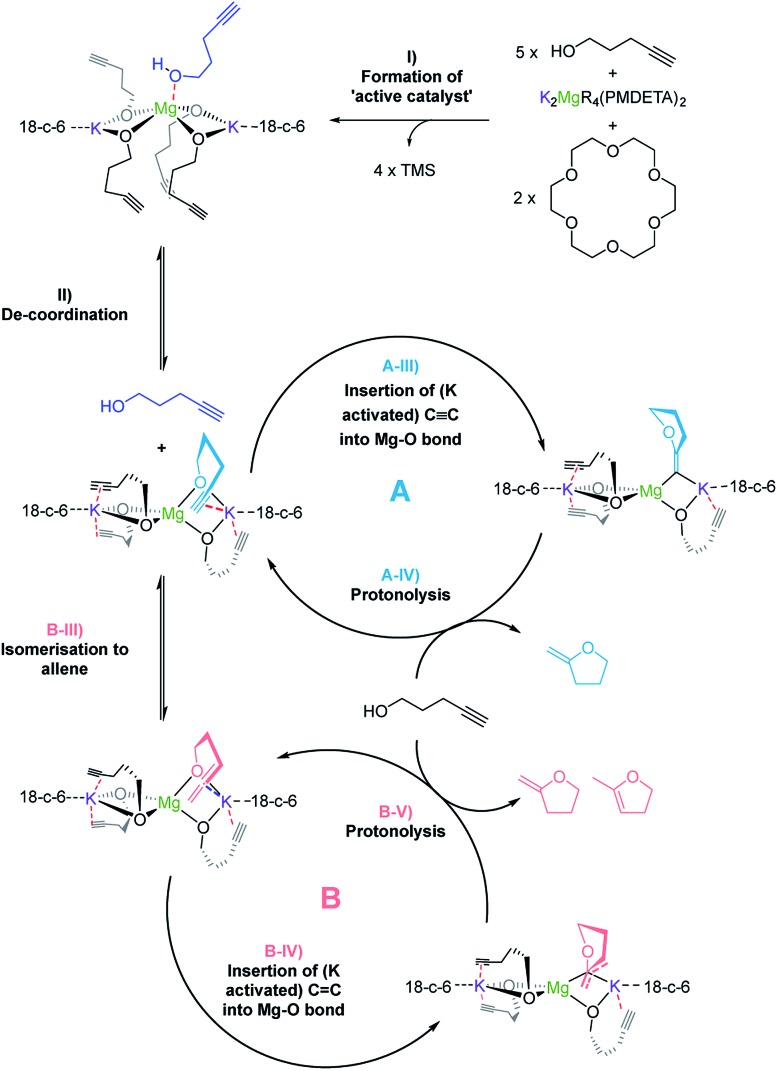
Proposed mechanism for the alkali metal magnesiate + crown ether co-catalyst mediated catalytic cyclisation of alkynols *via* dual activation.

In terms of the mechanism, there has been a previous proposition by Hill^62^ built upon the work of Marks.^38^ It reasons that there are two catalytic cycles at work simultaneously to allow for the production of the two product isomers observed. One cycle involves the alkyne passing through an allene intermediate, where it was demonstrated that starting from an allenyl alcohol instead of the alkynyl alcohol yielded the same products in a similar yield and ratio. In this work utilizing alkali metal magnesiates in combination with substrate **3**, we detected the presence of an allene intermediate (by ^1^H NMR spectroscopy, at *δ* 5.19 and 4.63 ppm) during the course of the reaction (Fig. 4). As such we believe that a similar two-cycle reaction mechanism is likely to be at play. The findings from the kinetics were also included with the other results to form a proposed mechanism for this alkali metal magnesiate-mediated catalysis (Scheme 4).

Overall the deduced mechanism starts with formation of the active catalyst from the magnesiate pre-catalyst. This involves the deprotonation of four alcohol substrate molecules to form a magnesiate alkoxide, liberating tetramethylsilane. This ‘active catalyst’ is then involved in a coordination/decoordination process with an additional substrate molecule as suggested by the kinetic studies. This additional molecule of **1** occupies the coordination sphere of the magnesium inhibiting cyclisation, giving rise to an inverse first order term in the substrate. Cyclisation (insertion of the carbon–carbon multiple bond into the magnesium oxygen bond) only occurs upon its decoordination.

In cycle A the carbon–carbon triple bond of the alkyne is directly inserted into the metal–oxygen bond, leading to the formation of only one product upon protonolysis. Cycle B on the other hand involves an equilibrium isomerisation from alkyne to allene which upon insertion into the metal–oxygen bond, and subsequent protonolysis, can yield two product isomers. Protonolysis releases the cyclised products and reforms the active catalyst completing the cycle.

Disclosing a unique cooperative behaviour under catalytic conditions, each of the metals plays a key role in this process, by simultaneously activating the O–H and C≡C bonds of the substrate. Thus, coordination of the C≡C bond to the larger more π-philic potassium centre enables further activation of this unsaturated group and brings it into close proximity to the di-anionic (magnesiate) {Mg(OR)_4_}^2–^ component, which is significantly more nucleophilic than a charge-neutral magnesium compound, facilitating intermolecular ring-closure to furnish the relevant oxygen-heterocycle.

## Experimental

### General considerations

All reactions were carried out under a protective atmosphere of argon using standard Schlenk techniques. Non-deuterated solvents were dried by heating to reflux over sodium ketyl radicals under nitrogen and deuterated solvents were degassed and stored over molecular sieves. Pre-catalysts [Mg(CH_2_SiMe_3_)_2_, K(CH_2_SiMe_3_), LiMg(CH_2_SiMe_3_)_3_, NaMg(CH_2_SiMe_3_)_3_, KMg(CH_2_SiMe_3_)_3_, Li_2_Mg(CH_2_SiMe_3_)_4_(TMEDA)_2_, Na_2_Mg(CH_2_SiMe_3_)_4_(TMEDA)_2_, K_2_Mg(CH_2_SiMe_3_)_4_(TMEDA)_2_ and K_2_Mg(CH_2_SiMe_3_)_4_(PMDETA)_2_] were prepared following literature procedures^2^ and handled in a glovebox. Li(CH_2_SiMe_3_), **1** and **5** were obtained from Sigma-Aldrich; **1** and **3** from Alfa-Aesar; and **7** from Fluorochem. Substrates **11**, **13**, **15**, **17**, **19** and **21** were synthesised based on literature procedures^82^ (characterisation data provided for new substrates and products **13–22** can be found in the ESI†). NMR spectra were recorded on a Bruker AV III 400 MHz spectrometer operating at 400.1 MHz for ^1^H, 100.6 MHz for ^13^C or 128.4 MHz for ^19^F.

### Procedure for pre-catalyst synthesis

Pre-catalysts were prepared and isolated prior to being employed in reactions. Na(CH_2_SiMe_3_),^83^,^84^ K(CH_2_SiMe_3_)^84^,^85^ and Mg(CH_2_SiMe_3_)_2_ (ref. 69) were prepared from literature procedures. TMEDA and PMDETA were distilled over CaH_2_ before use. K_2_Mg(CH_2_SiMe_3_)_4_(PMDETA)_2_ was prepared by suspending K(CH_2_SiMe_3_) (0.25 g, 2 mmol) and Mg(CH_2_SiMe_3_)_2_ (0.20 g, 1 mmol) in hexane (10 mL). The mixture was stirred at ambient temperature for 1 h. PMDETA (0.43 mL, 2 mmol) was added to this white suspension and the mixture was gently heated until homogeneity was achieved. After storage at –26 °C overnight, the mixture was filtered to yield a light yellow solid (typical yield; 0.62 g, 78%).

Li_2_Mg(CH_2_SiMe_3_)_4_(TMEDA)_2_, Na_2_Mg(CH_2_SiMe_3_)_4_(TMEDA)_2_, and K_2_Mg(CH_2_SiMe_3_)_4_(TMEDA)_2_ were prepared using similar methods to K_2_Mg(CH_2_SiMe_3_)_4_(PMDETA)_2_ 69 with the following modifications: substitution of TMEDA (0.30 mL, 2 mmol); Li(CH_2_SiMe_3_) (2 mL, 2 M in pentanes); and/or Na(CH_2_SiMe_3_) (0.22 g, 2 mmol) where appropriate.

LiMg(CH_2_SiMe_3_)_3_,^70^ NaMg(CH_2_SiMe_3_)_3_,^32^ and KMg(CH_2_SiMe_3_)_3_ were prepared using half the molar quantity of M(CH_2_SiMe_3_)_3_ (1 mL/0.11 g/0.13 g respectively), and toluene (5 mL) was added in place of the multidentate amine in order to achieve dissolution upon heating.

### Procedure for NMR scale reactions

In the catalytic cyclisation of 4-pentynol (**1**) with K_2_Mg(CH_2_SiMe_3_)_4_(PMDETA)_2_ + 18-c-6, 4-pentynol (56 μL, 0.6 mmol, 1.2 eq.) (0.2 eq./20 mol% excess required to form the ‘active catalyst’) was added to a Young's tap NMR tube alongside C_6_D_6_ (0.54 mL), 1,2,3,4-tetraphenylnaphthalene (0.022 g, 0.05 mmol, 0.1 eq.) and 18-crown-6 (0.013 g, 0.05 mmol, 0.1 eq.). To this K_2_Mg(CH_2_SiMe_3_)_4_(PMDETA)_2_ (0.020 g, 0.025 mmol, 0.05 eq.) was added and the tube was placed in an oil bath at 75 °C. The reaction was periodically monitored by ^1^H NMR spectroscopy and the yields obtained were calculated using NMR spectroscopic integrals and were relative to the internal standard. Optimisation and substrate scope reactions were carried out using similar procedures, employing 10 mol% of an appropriate internal standard [1,2,3,4-tetraphenylnaphthalene (0.022 g) or ferrocene (0.009 g)] and with the oil bath temperature being changed where necessary based on observed reaction times. 0.5 mmol alkynol substrate was used with the monometallic species and lower-order magnesiates, and 0.6 mmol with higher-order magnesiate pre-catalysts. 0.025 mmol pre-catalyst was employed in all cases, quantities shown in Table 3. In general, the reactions remain homogeneous until nearing completion. At this point an unidentified solid/gel forms, which is presumably a species akin to the ‘K_2_Mg(OAlk)_4_(18-c-6)_2_ resting-state’.

### Procedure for kinetic studies

In the kinetic studies to determine the rate dependence of alkynol in the cyclisation of 4-pentynol (**1**) with K_2_Mg(CH_2_SiMe_3_)_4_(PMDETA)_2_ + 18-c-6, 4-pentynol (56 μL, 0.6 mmol) was placed in a Young's tap NMR tube with C_6_D_6_ (0.6 mL), 1,2,3,4-tetraphenylnaphthalene (0.022 g, 0.05 mmol) and 18-crown-6 (0.010 g, 0.04 mmol). To this K_2_Mg(CH_2_SiMe_3_)_4_(PMDETA)_2_ (0.016 g, 0.02 mmol) was added. The reaction was maintained at 70 °C in the NMR spectrometer and was monitored by ^1^H NMR spectroscopy. Yields were calculated using NMR spectroscopic integrals characteristic of **2a** relative to the internal standard, 1,2,3,4-tetraphenylnaphthalene. This procedure was repeated with 0.90, and 1.20 mmol alkynol (**1**) to provide the data presented in Fig. S1† which was subsequently used in Fig. 2.

The same procedure was applied to the investigation of the dependence of the initial rates of the reaction on the catalyst. The same concentration of **1** was used while varying the concentration of the catalyst. In terms of the pre-catalyst, 0.025, 0.030 and 0.035 mmol quantities of K_2_Mg(CH_2_SiMe_3_)_4_(PMDETA)_2_ were employed, necessitating 0.05, 0.06 and 0.07 mmol 18-crown-6 co-catalyst. The data from these experiments with varying catalyst concentration are presented in Fig. S2† and are subsequently used in Fig. 3.

## Conclusions

In summary, alkali metal magnesiates have been shown to successfully promote the catalytic intramolecular hydroalkoxylation of alkynols through cooperative bimetallic catalysis. The roles of both magnesium and potassium components are crucial for the success of the process, affording a unique type of substrate activation that is not possible in conventional single-metal systems. Through cooperativity, the utilisation of an alkali metal magnesiate has overcome the inherent problems associated with homometallic magnesium systems. The optimised catalyst system, (PMDETA)_2_K_2_Mg(CH_2_SiMe_3_)_4_ paired with 18-crown-6 has been shown to function well with both terminal and internal alkyne substrates having a range of functional groups. Kinetic studies have revealed an inhibition effect of substrate on the catalyst under standard conditions (high concentrations of alkynol) by the formation of a coordination adduct which requires dissociation prior to the cyclisation step. Initial reactivity studies suggest that coordination of the 18-crown-6 to K finely tunes the reactivity of the bimetallic system, probably minimising the coordination of additional substrate molecules to the catalyst.

## Conflicts of interest

There are no conflicts to declare.

## Supplementary Material

Supplementary information
