# Supplementary material for: s-Block cooperative catalysis: alkali metal magnesiate-catalysed cyclisation of alkynols
Source: Chem Sci. 2019 May 14;10(22):5821–31. doi: 10.1039/c9sc01598a (PMC6568277; doi:10.1039/c9sc01598a)
Supplement: Supplementary file 1 [file SC-010-C9SC01598A-s001.pdf]

Electronic Supplementary Material (ESI) for Chemical Science.
This journal is © The Royal Society of Chemistry 2019

Supporting information

s-Block cooperative catalysis: alkali metal magnesiate-catalysed cyclisation of alkynols

Michael Fairley, Laia Davín, Alberto Hernán-Gómez, Joaquín García-Álvarez*, Charles T. O´Hara* and Eva Hevia*

Contents

General considerations 1
Procedure for pre-catalyst synthesis 2
Procedure for NMR scale reactions 2
Procedure for kinetic studies 2
Kinetics plots referred to in main text 3
Catalytic cyclisation reactions 4
Synthesis & characterisation of new alkynol and cyclic products 11
References 26

General considerations

All reactions were carried out under a protective atmosphere of argon using standard Schlenk techniques. Non-deuterated solvents were dried by heating to reflux over sodium ketyl radical under nitrogen and deuterated solvents were degassed and stored over

molecular sieves. Pre-catalysts [$Mg(CH_2SiMe_3)_2$, $K(CH_2SiMe_3)$, $LiMg(CH_2SiMe_3)_3$, $NaMg(CH_2SiMe_3)_3$, $KMg(CH_2SiMe_3)_3$, $Li_2Mg(CH_2SiMe_3)_4(TMEDA)_2$, $Na_2Mg(CH_2SiMe_3)_4(TMEDA)_2$, $K_2Mg(CH_2SiMe_3)_4(TMEDA)_2$ and $K_2Mg(CH_2SiMe_3)_4(PMDETA)_2$] were prepared following literature procedures[S1] and handled in a glovebox. $Li(CH_2SiMe_3)$, **1** and **5** were obtained from Sigma-Aldrich; **1** and **3** from Alfa-Aesar; and **7** from Fluorochem. Substrates **11**, **13**, **15**, **17**, **19** and **21** were synthesised based on literature procedure.[S2] NMR spectra were recorded on a Bruker AVIII 400 MHz spectrometer operating at 400.1 MHz for ^{1}H, 100.6 MHz for ^{13}C or 128.4 MHz for ^{19}F.

Procedure for pre-catalyst synthesis

Pre-catalysts were prepared and isolated prior to being employed in reactions. $Na(CH_2SiMe_3)$, $K(CH_2SiMe_3)$ and $Mg(CH_2SiMe_3)_2$ were prepared from literature prcoedures.[S1] TMEDA and PMDETA were distilled over CaH_2 before use.

$K_2Mg(CH_2SiMe_3)_4(PMDETA)_2$ was prepared by suspending $K(CH_2SiMe_3)$ (0.25 g, 2 mmol) and $Mg(CH_2SiMe_3)_2$ (0.20 g, 1 mmol) in hexane (10 mL). The mixture was stirred at ambient temperature for 1 h. PMDETA (0.43 mL, 2 mmol) was added to this white suspension and the mixture gently heated until homogeneity was achieved. After storing at −26°C overnight, the mixture was filtered to yield a pale yellow solid (typical yield; 0.62 g, 78%).

$Li_2Mg(CH_2SiMe_3)_4(TMEDA)_2$, $Na_2Mg(CH_2SiMe_3)_4(TMEDA)_2$, $K_2Mg(CH_2SiMe_3)_4(TMEDA)_2$ were prepared using similar methods to $K_2Mg(CH_2SiMe_3)_4(PMDETA)_2$ with the following modifications: substitution of TMEDA (0.30 mL, 2 mmol); $Li(CH_2SiMe_3)$ (2 mL, 2 M in pentanes); and/or $Na(CH_2SiMe_3)$ (0.22 g, 2 mmol) where appropriate.

$LiMg(CH_2SiMe_3)_3$, $NaMg(CH_2SiMe_3)_3$, $KMg(CH_2SiMe_3)_3$ were prepared using half the molar quantity of $M(CH_2SiMe_3)_3$ (1 mL / 0.11 g/0.13g respectively), and toluene (5 mL) was added in place of the multidentate amine in order to achieve dissolution upon heating.

Procedure for NMR scale reactions

In the catalytic cyclisation of 4-pentynol (**1**) with $K_2Mg(CH_2SiMe_3)_4(PMDETA)_2$ + 18-C-6, 4-pentynol (56 µL, 0.6 mmol, 1.2 eq.) (0.2 eq. / 20 mol% excess required to form the 'active catalyst') was added to a Young's tap NMR tube alongside C_6D_6 (0.54 mL), 1,2,3,4,-tetraphenylnaphthalene (0.022 g, 0.05 mmol, 0.1 eq.) and 18-crown-6 (0.013 g, 0.05 mmol, 0.1 eq.). To this $K_2Mg(CH_2SiMe_3)_4(PMDETA)_2$ (0.020 g, 0.025 mmol, 0.05 eq.) was added and the tube placed in an oil bath at 75°C. The reaction was periodically monitored by ^{1}H NMR spectroscopy and the yields obtained were calculated using NMR spectroscopic integrals and are relative to the internal standard. Optimisation and substrate scope reactions were carried out using similar procedures, employing 10 mol% of an appropriate internal standard [1,2,3,4-tetraphenylnaphthalene (0.022 g) or ferrocene (0.009 g)] and with the oil bath temperature being changed where necessary based on observed reaction times. 0.5 mmol of alkynol substrate was used with the monometallic species and lower-order magnesiates, and 0.6 mmol with higher-order magnesiate pre-catalysts. 0.025 mmol pre-catalyst was employed in all cases, quantities shown in Table 4.

Table S1 - Quantities of pre-catalysts employed in the catalytic cyclisation reactions

Entry	Pre-catalyst (R = CH_2SiMe_3)	Mass	Entry	Pre-catalyst	Mass
1	MgR_2	0.005 g	6	$Li_2MgR_4(TMEDA)_2$	0.015 g
2	KR	0.003 g	7	$Na_2MgR_4(TMEDA)_2$	0.016 g
3	$LiMgR_3$	0.007 g	8	$K_2MgR_4(TMEDA)_2$	0.017 g
4	$NaMgR_3$	0.008 g	9	$K_2MgR_4(PMDETA)_2$	0.020 g
5	$KMgR_3$	0.008 g			

Procedure for kinetic studies

In the kinetic studies to determine the rate dependence of alkynol in the cyclisation of 4-pentynol (**1**) with $K_2Mg(CH_2SiMe_3)_4(PMDETA)_2$ + 18-C-6, 4-pentynol (56 µL, 0.6 mmol) was placed in a Young's tap NMR tube with C_6D_6 (0.6 mL), 1,2,3,4-tetraphenylnaphthalene (0.022 g, 0.05 mmol) and 18-crown-6 (0.010 g, 0.04 mmol). To this $K_2Mg(CH_2SiMe_3)_4(PMDETA)_2$ (0.016 g, 0.02 mmol) was added. The reaction was maintained at 70°C in the NMR spectrometer and was monitored by ^{1}H NMR spectroscopy. Yields were calculated using NMR spectroscopic integrals characteristic to **2a** relative to the internal standard, 1,2,3,4-tetraphenylnaphthalene. This procedure was repeated with 0.90, 1.20 and 1.35 mmol of alkynol (**1**) to provide the data presented in Figure 4.

The same procedure was applied to the investigation of the dependence on catalyst of the reaction. The same quantity of solvent and standard were used along with a fixed 1.20 mmol quantity of alkynol (**1**). In terms of pre-catalyst, 0.025, 0.030 and 0.035 mmol quantities of $K_2Mg(CH_2SiMe_3)_4(PMDETA)_2$ were employed, necessitating 0.05, 0.06 and 0.07 mmol of 18-crown-6 co-catalyst. The data from these experiments varying catalyst concentration is presented in Figure 6.

Kinetics plots referred to in main text

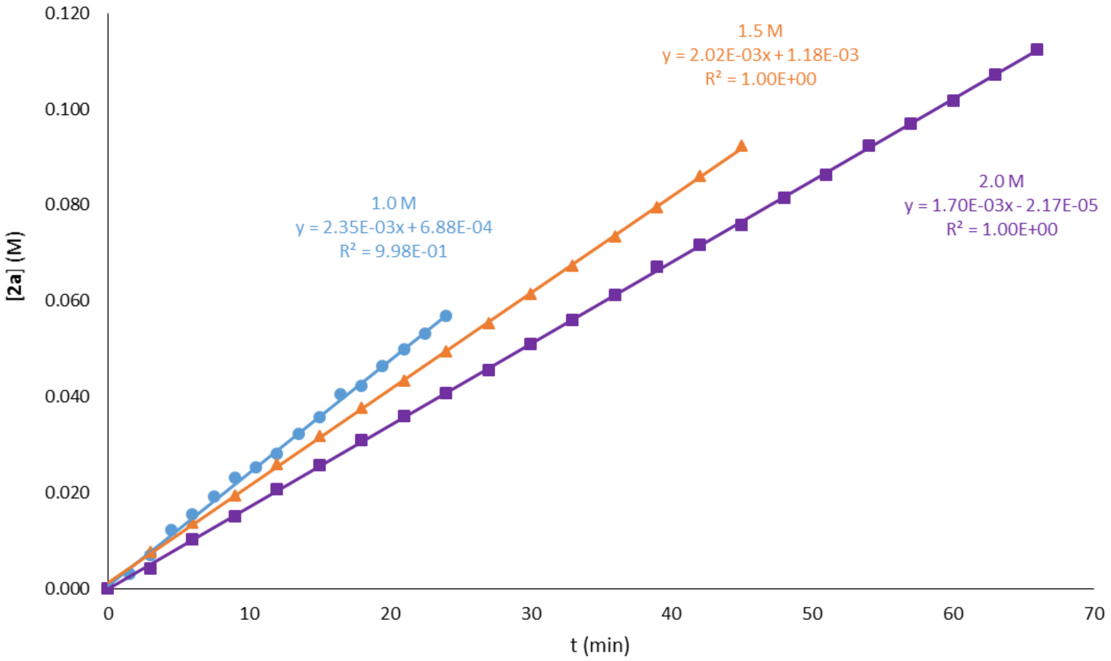

S2 - Production of product **2a** (0.033 M cat., C_6D_6, 343 K) at varied substrate concentrations (**1**) (1.0 - 2.0 M)

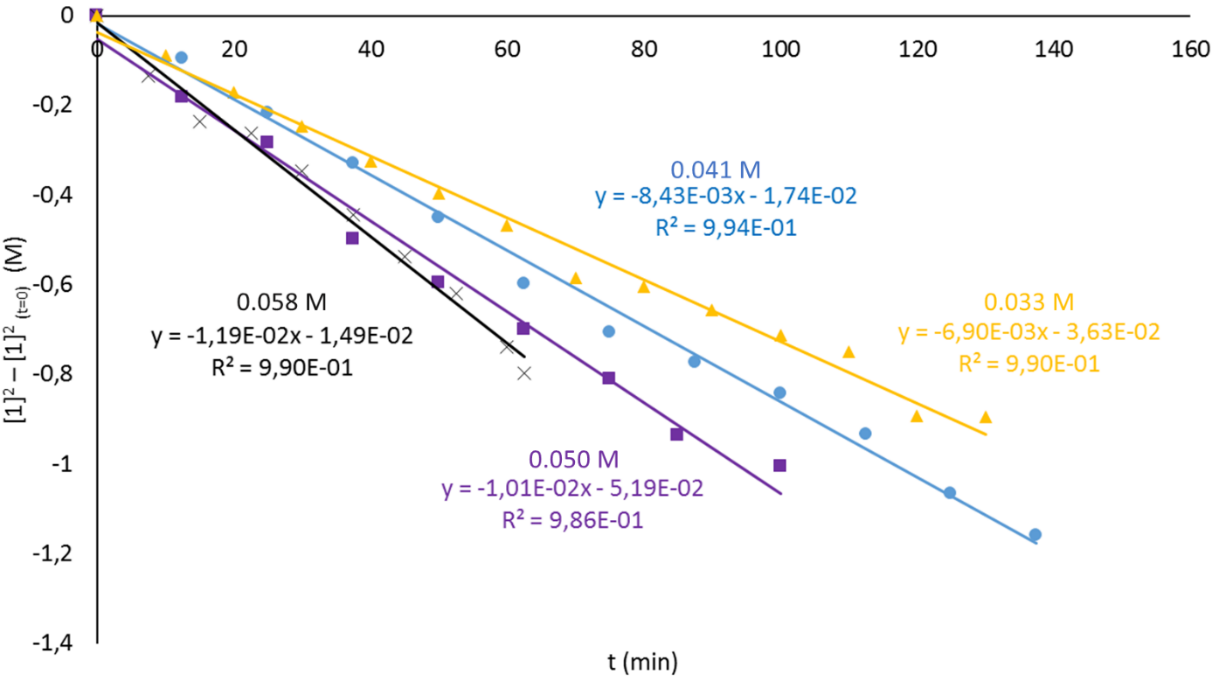

S1 - Consumption of 4-pentynol **1** (C_6D_6, 343 K) at varied catalyst loadings (0.033 - 0.058 M)

Catalytic cyclisation reactions

Optimisation – (2a) 2-methylenetetrahydrofuran + (2b) 5-methyl-2,3-dihydrofuran[S3]

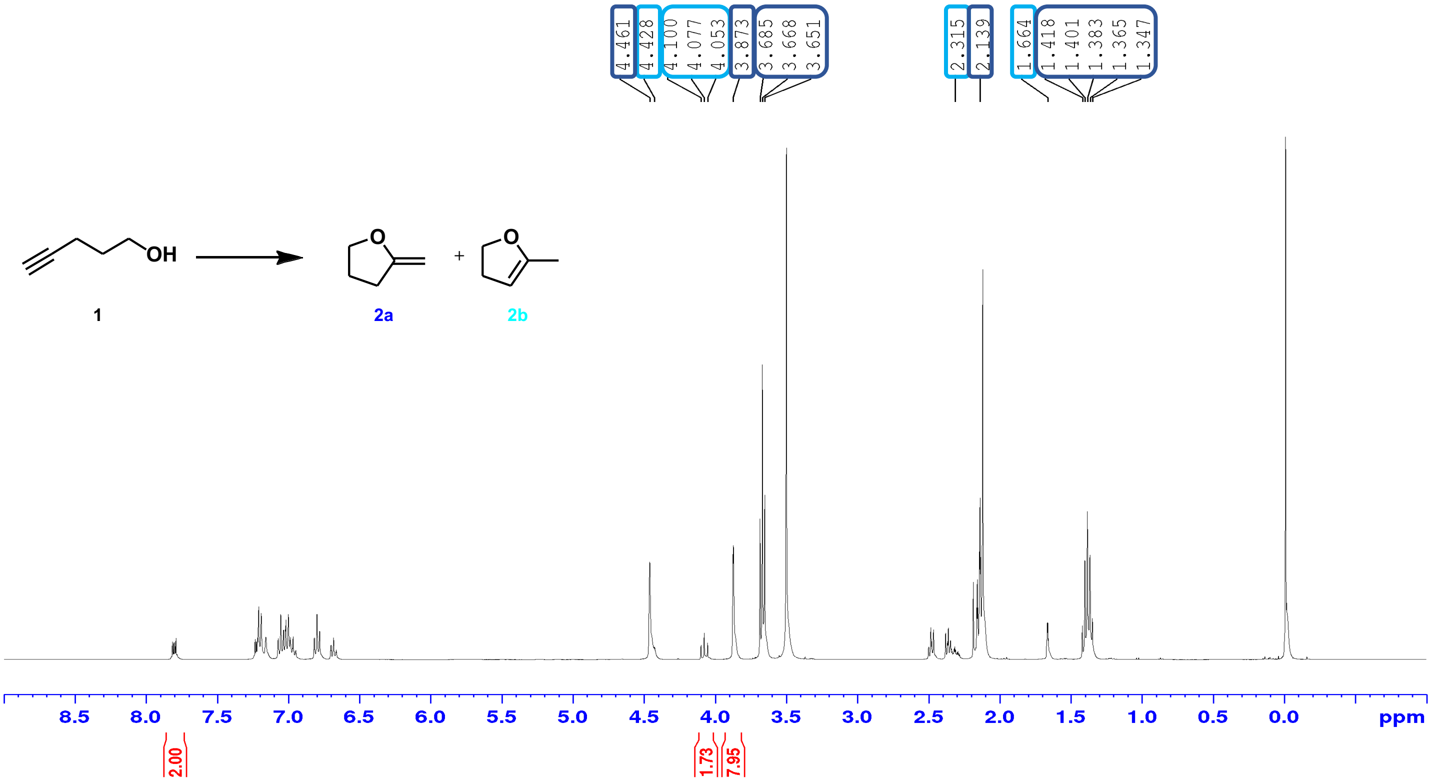

S3 – Products 2a + 2b in C_6D_6, with 1,2,3,4-tetraphenylnapthalene standard, yield 88% (90:10) [catalyst - $K_2MgR_4(PMDETA)_4$ + 18-C-6]

Table 2, entry 1 - (4a) 2-Methylenetetrahydropyran, (4b) 6-methy-3,4-dihyropyran and (4c) hex-4-yn-1-ol[S4]

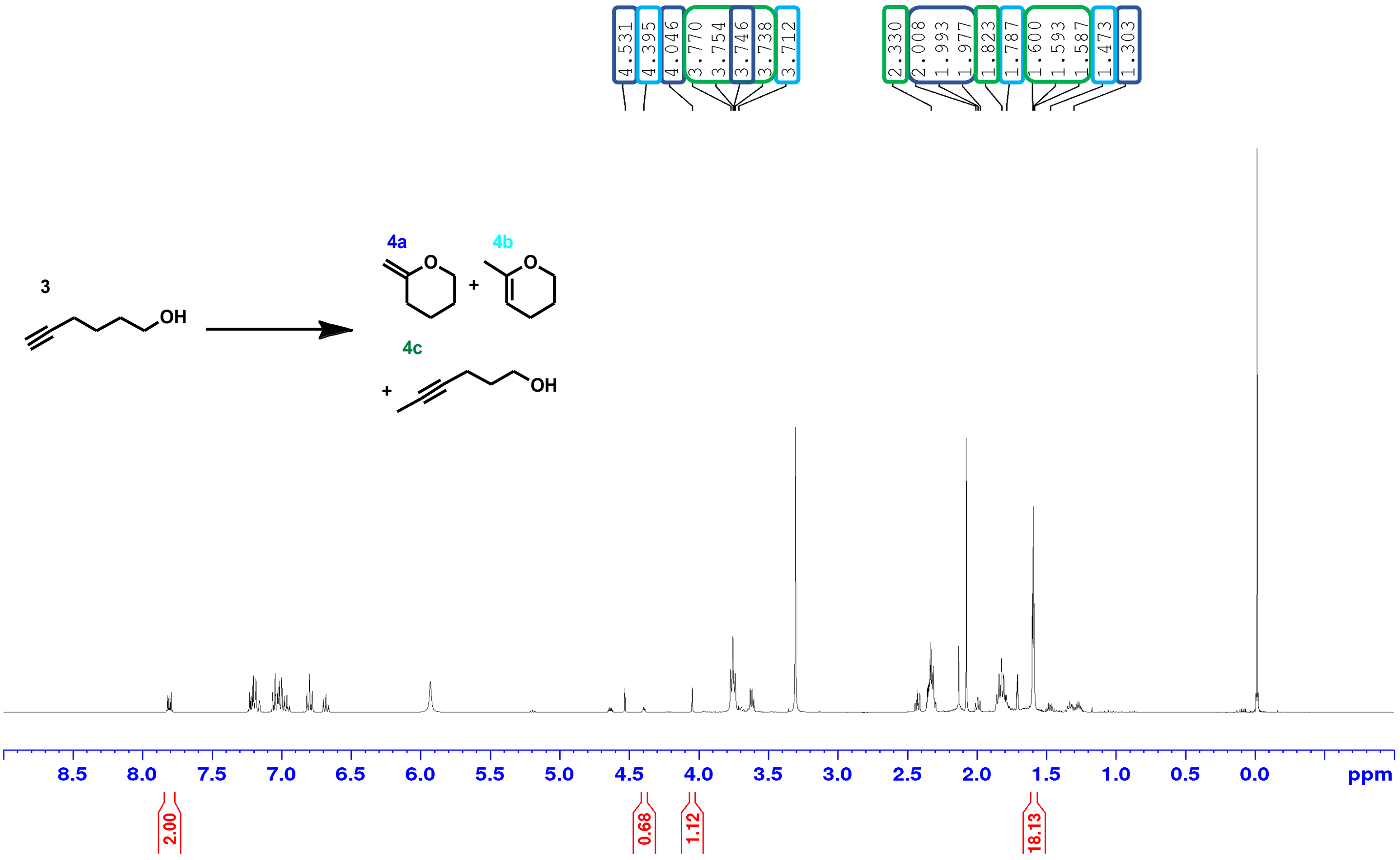

S4 - Products 4a + 4b +4c in C_6D_6, with 1,2,3,4-tetraphenylnapthalene standard, yield 98% (14:9:77) [75°C, 1 h]

Table 2, entry 2 - (6) 1-methylene-1,3-dihydroisobenzofuran[S5]

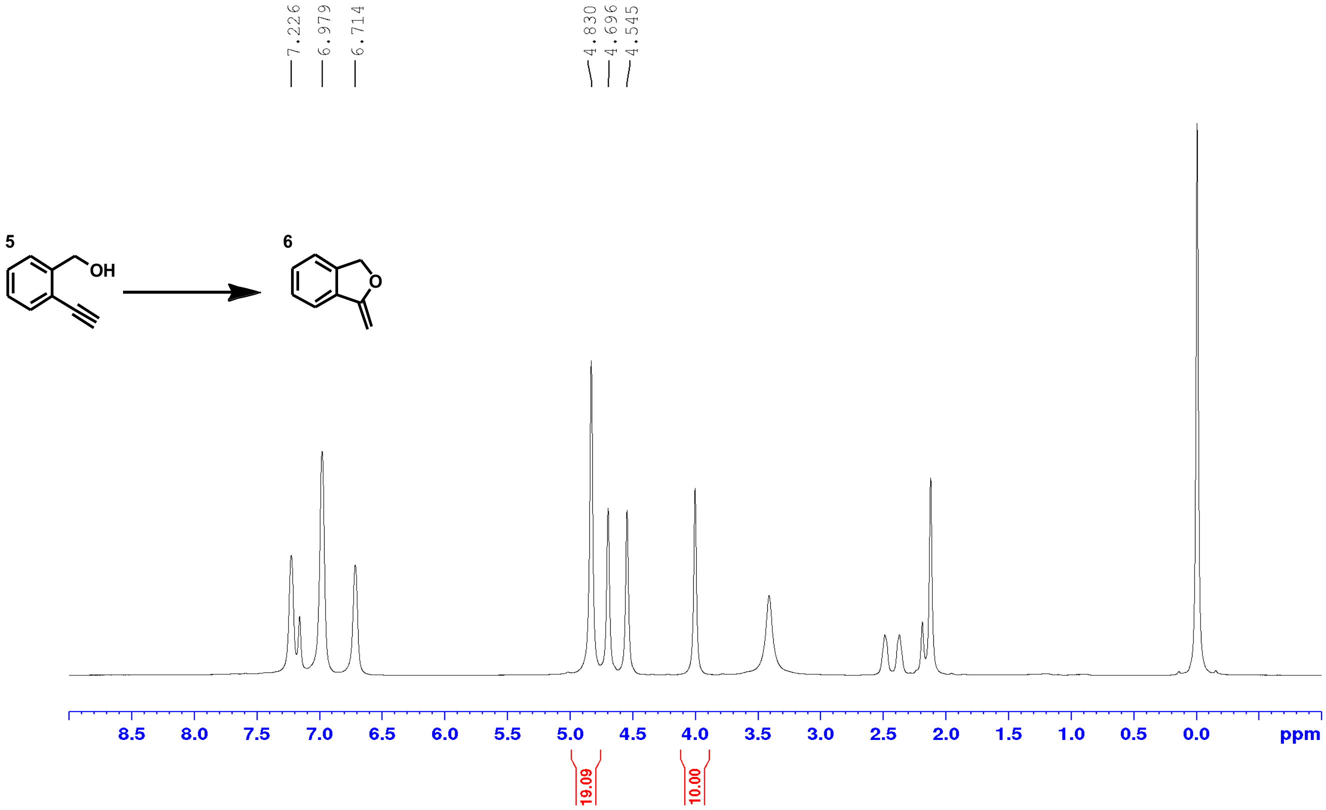

S5 - Product 6 in C_6D_6, with ferrocene standard, yield 86 % [rt., 1 h]

Table 2, entries 3 & 4 – (8a) 2,3-dimethylfuran and (8b) 2-methylene-3-methyl-2,5-dihydrofuran[S6]

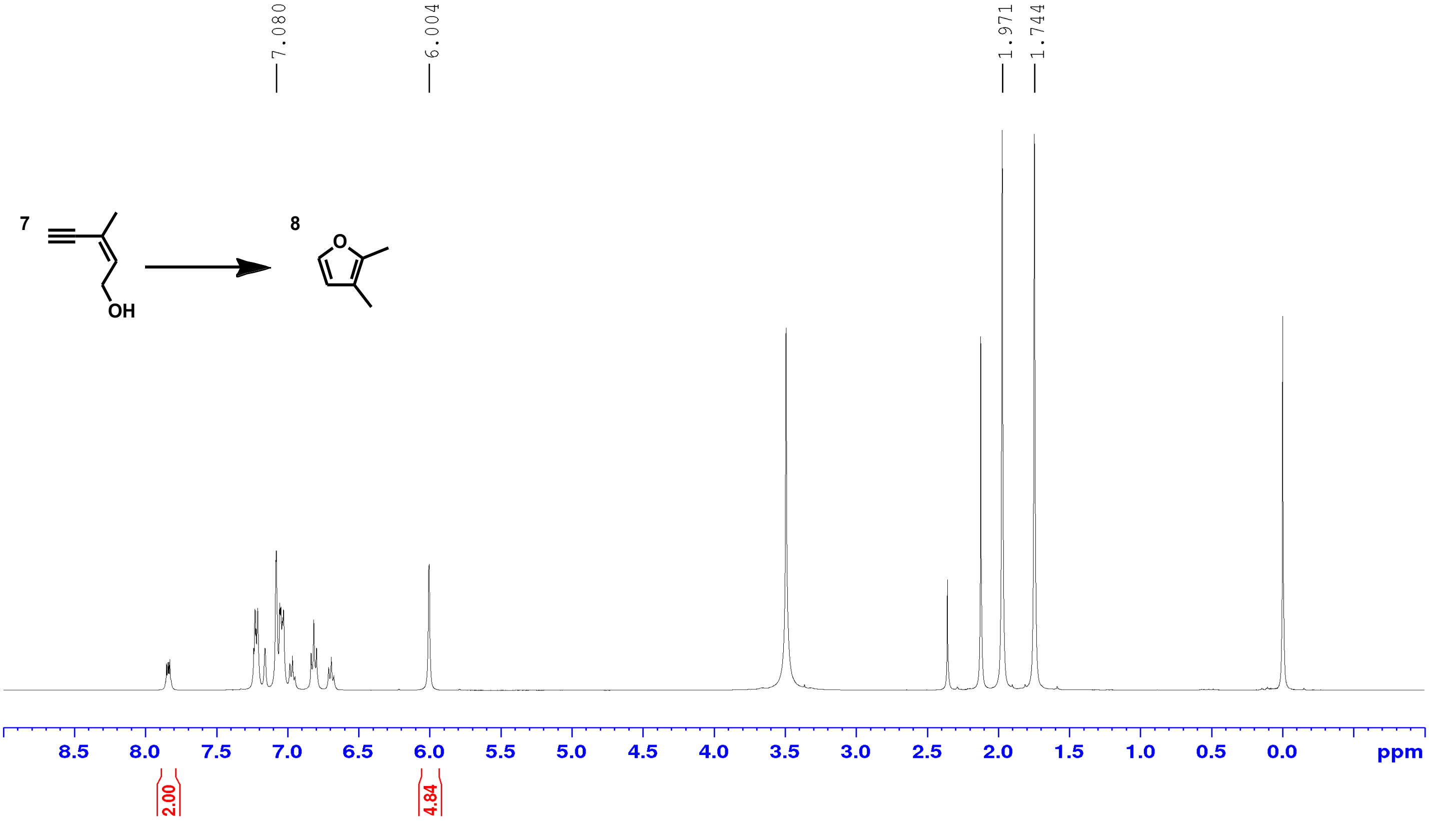

S6 - Product 8 in C_6D_6, with 1,2,3,4-tetraphenylnapthalene standard, yield 89% [75°C, 1 h]

Table 2, entries 5 & 6 – (10a) 2-benzyl-3-methylfuran and (10b) 2-benzylidene--3-methyl-2,5-dihydrofuran[S6]

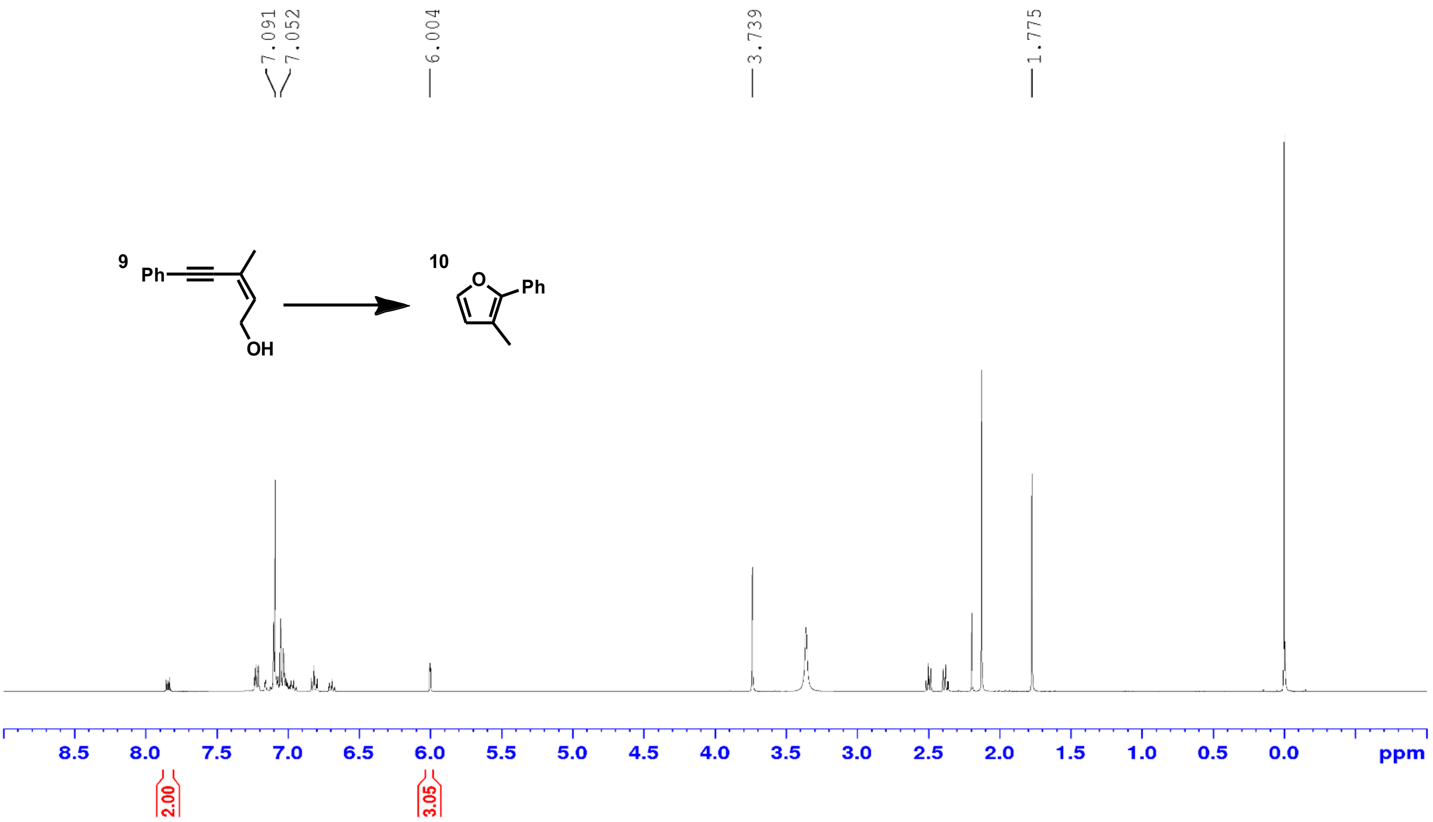

S7 - Product 9 in C_6D_6, with 1,2,3,4-tetraphenylnapthalene standard, yield 89% [75°C, 1 h]

Table 3, entry 1 – (12a/b) (E/Z)-2-benzylidenetetrahydrofuran[S7]

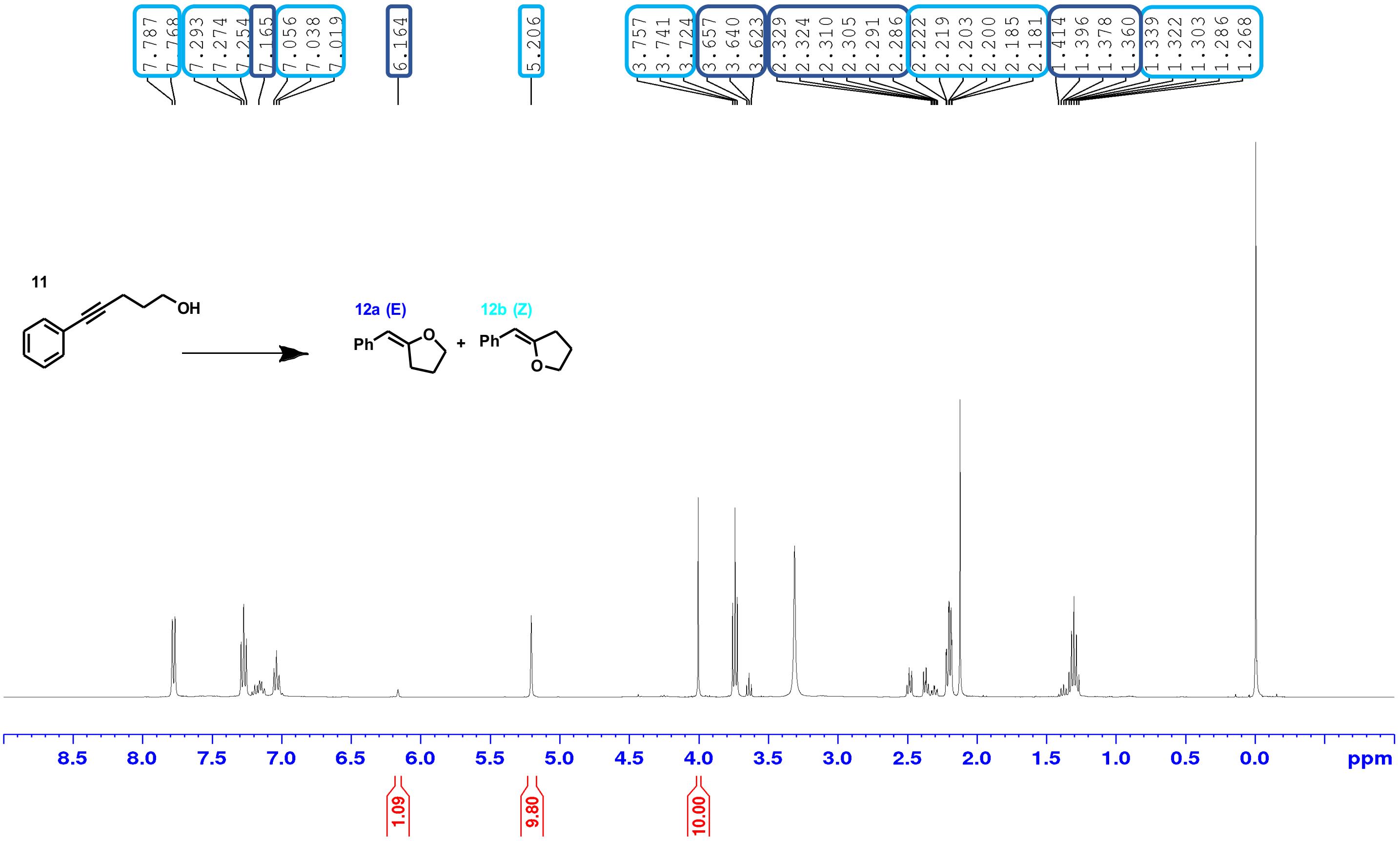

S9 - Products 12a + 12b in C_6D_6, with ferrocene standard, yield 84% (10:90). [75°C, 1 h]

Table 3, entry 2 - (14a/b) (E/Z)-2-(4-methoxybenzylidene)tetrahydrofuran

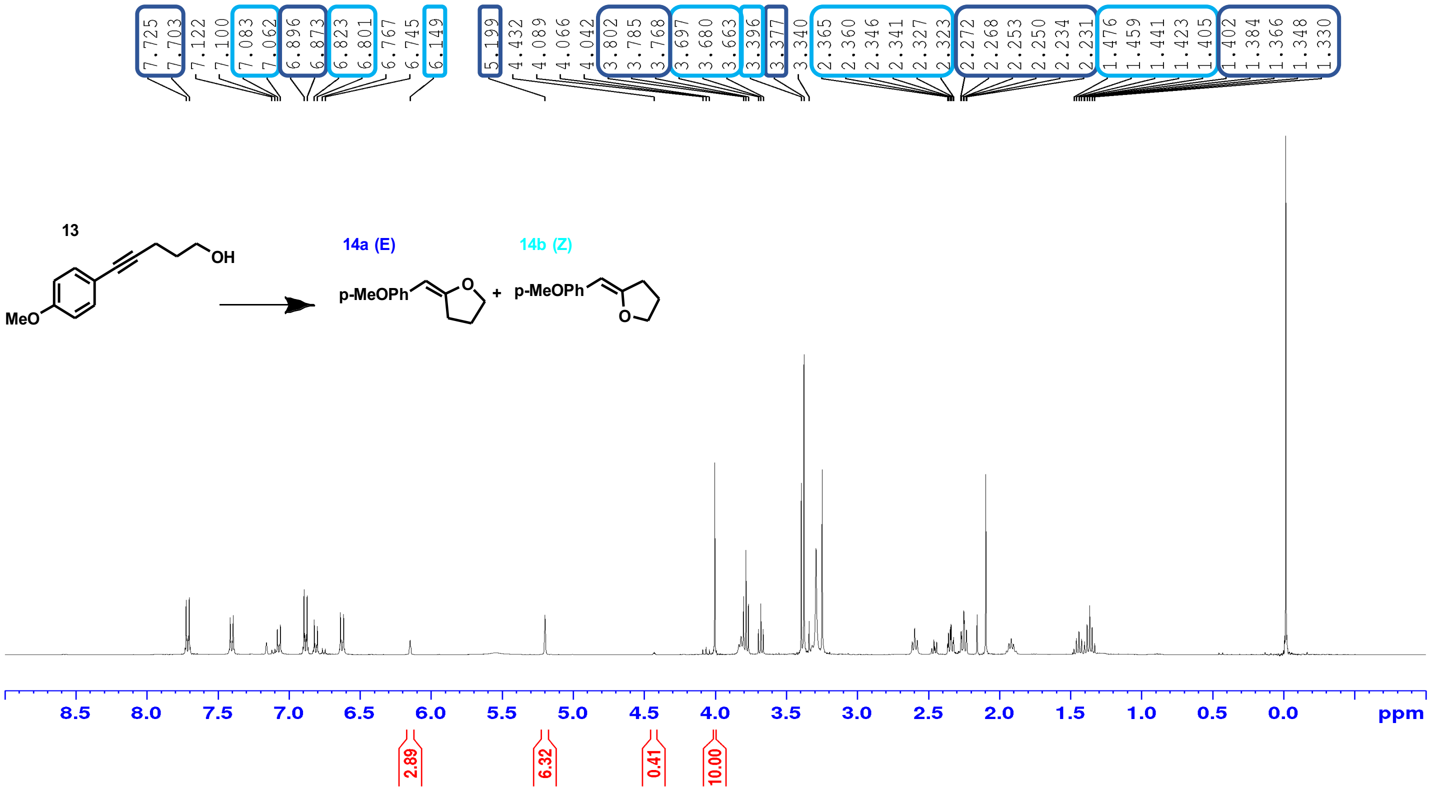

S 10 - Products 14a + 14b in C_6D_6, with ferrocene standard, yield 64% (31:69). [75°C, 36 h]

Table 3, entry 3 - (16a/b) (E/Z)-2-(4-cholorobenzylidene)tetrahydrofuran

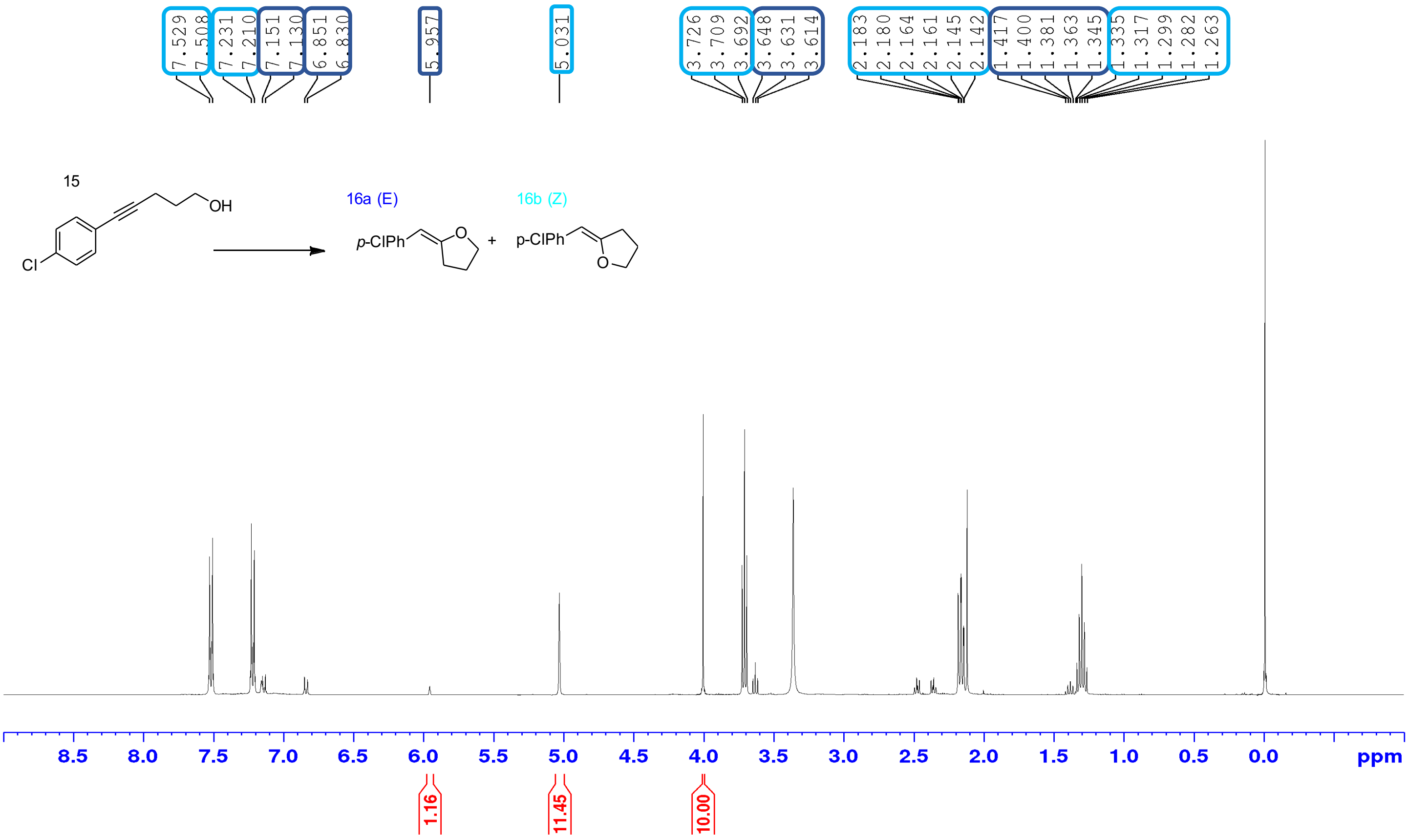

S11 - Products 16a + 16b in C_6D_6, with ferrocene standard, yield 99% (9:91). [75°C, 1 h]

Table 3, entry 4 - (18a/b) (E/Z)-2-(3-fluorobenzylidene)tetrahydrofuran

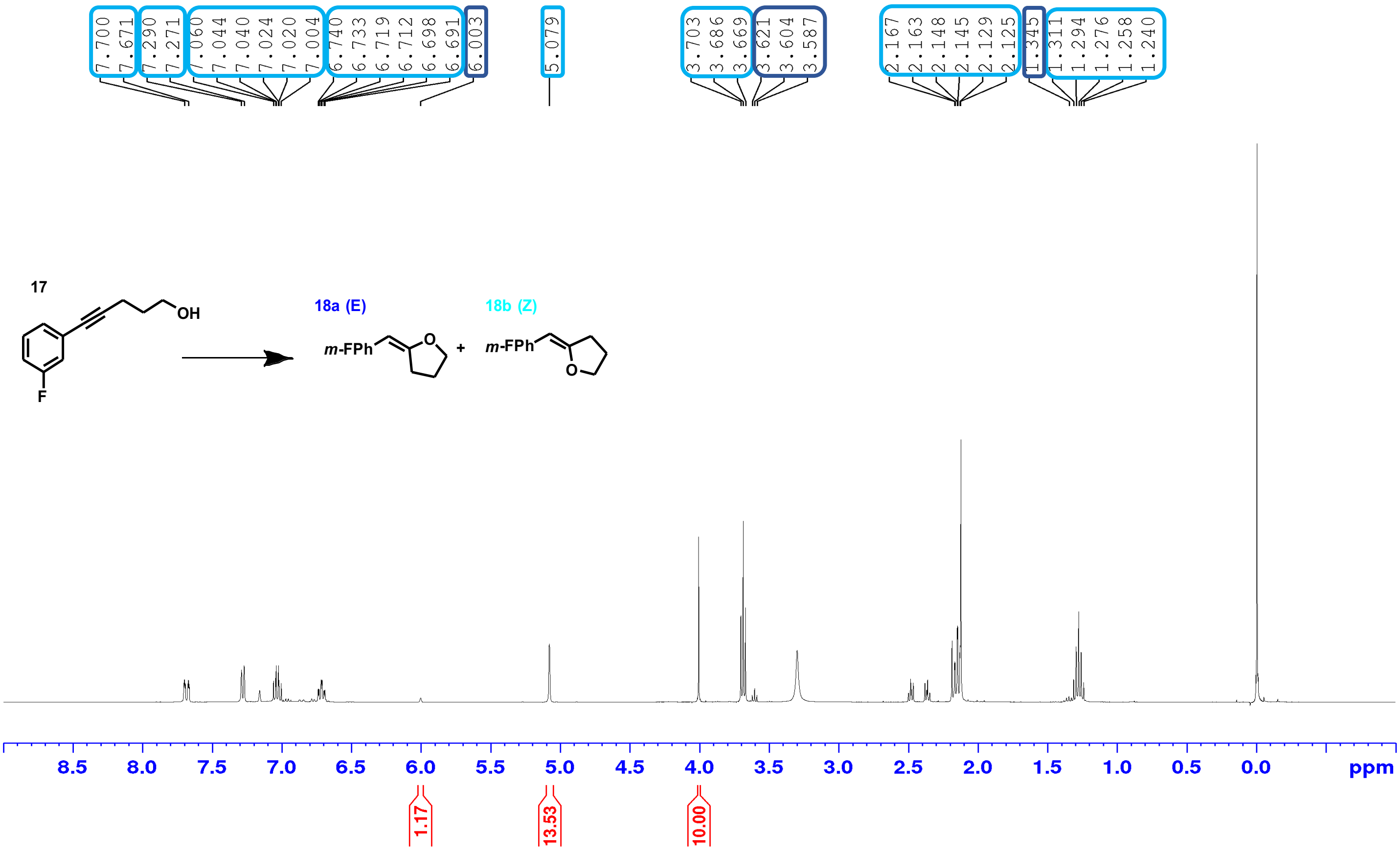

S12 - Products 18a + 18b in C_6D_6, with ferrocene standard, yield 95% (8:92). [75°C, 0.5 h]

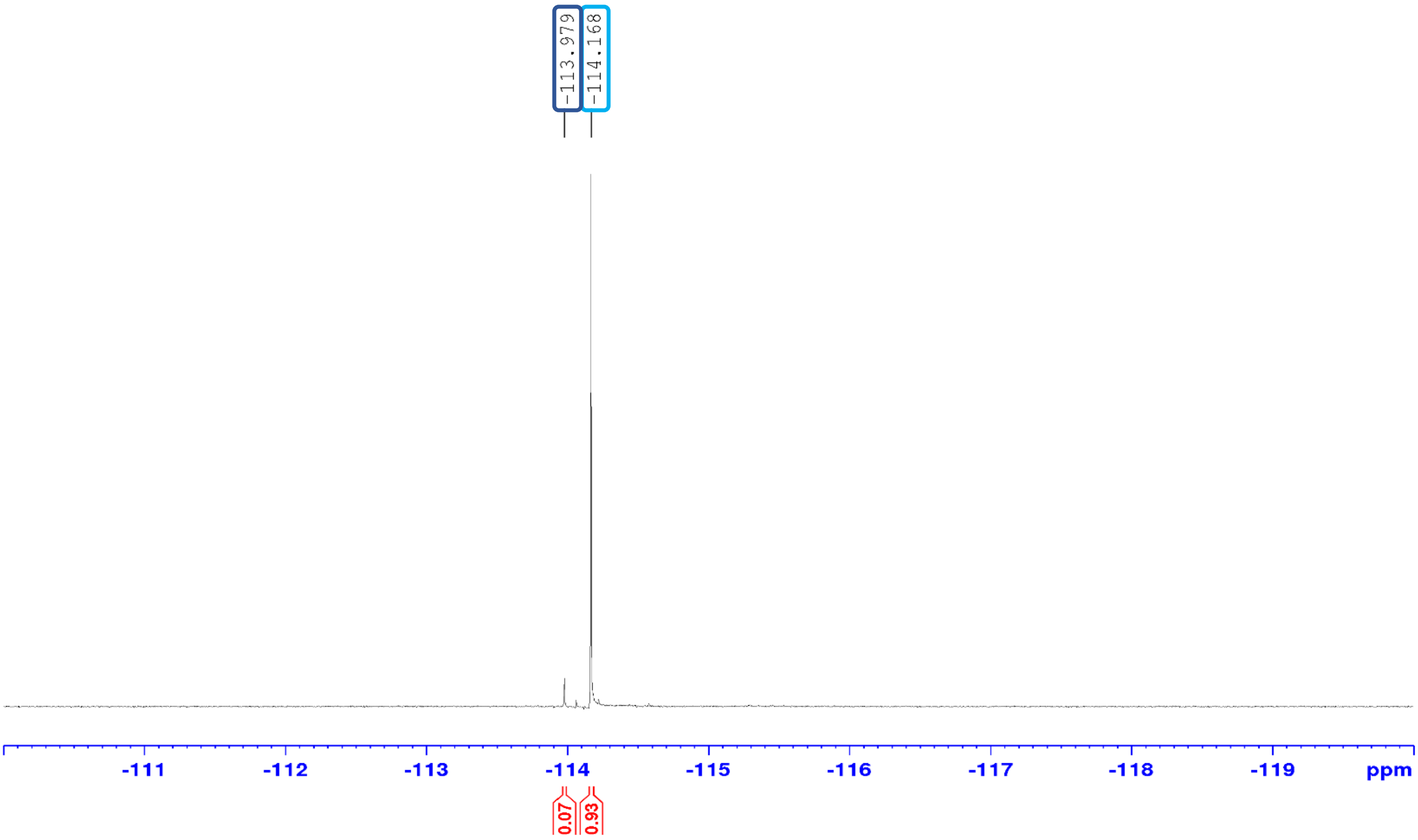

S13 - ^{19}F spectrum of products 18a + 18b in C_6D_6. [75°C, 0.5 h]

Table 3, entry 5 – (20a/b) (E/Z)-2-(3,5-(bistrifluoromethyl)benzylidene)tetrahydrofuran

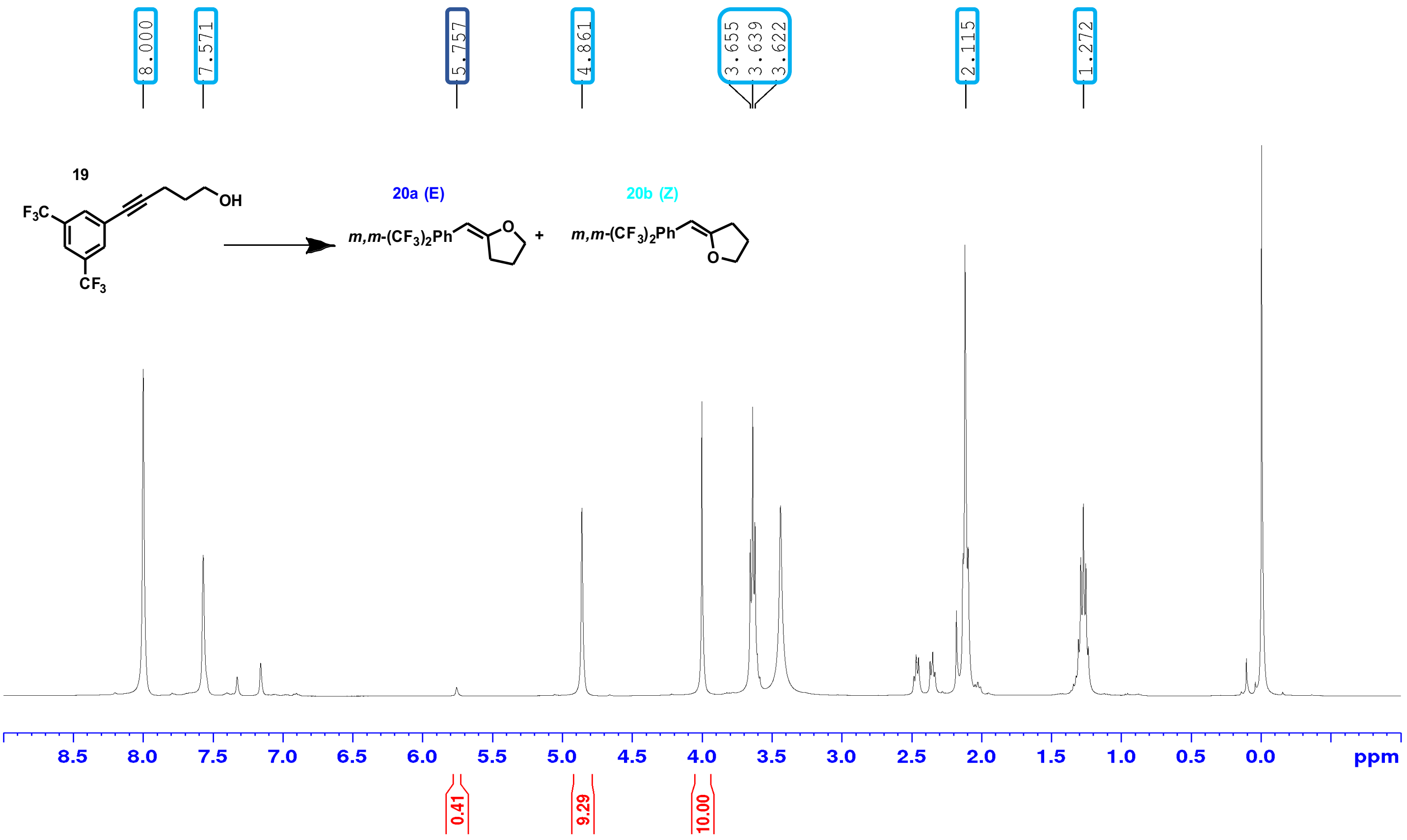

S14 - Products 20a + 20b in C_6D_6, with ferrocene standard, yield 97% (4:96). [75°C, 0.5 h]

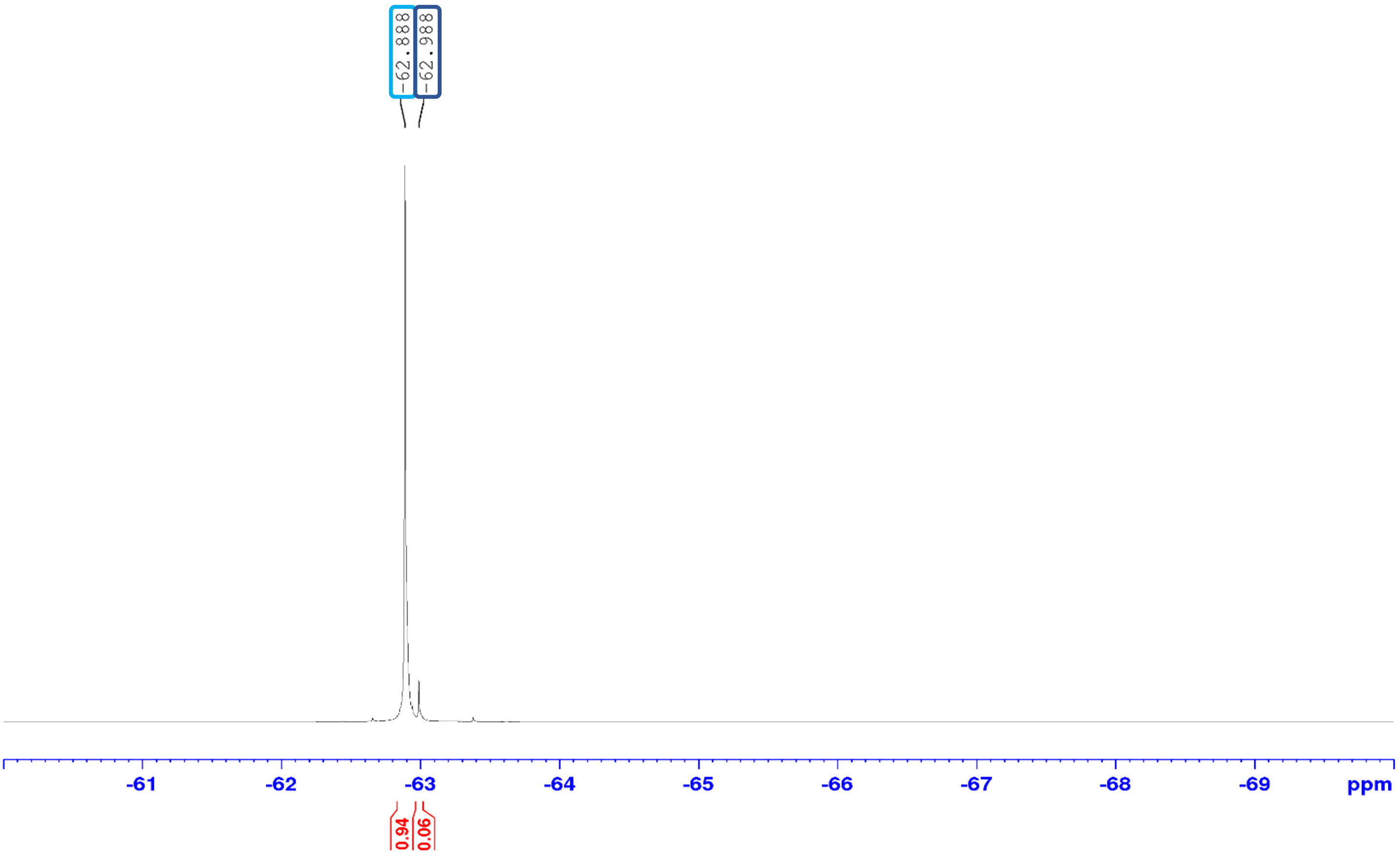

S15 - ^{19}F spectrum of products 20a + 20b in C_6D_6. [75°C, 0.5 h]

Table 3, entry 6 – (22a/b) (E/Z)-4-((dihydrofuran-2(3H)-ylidene)methyl)benzonitrile

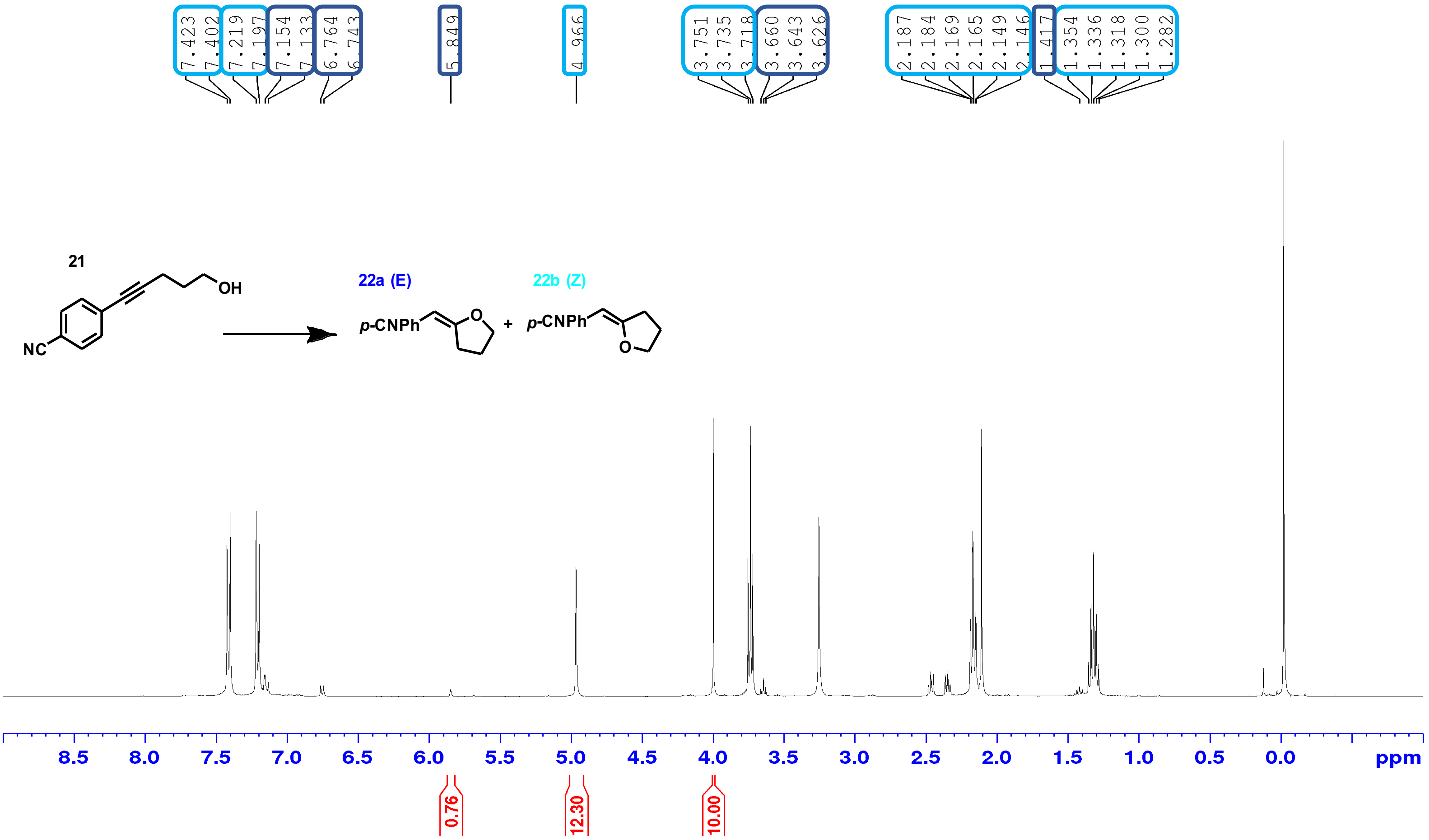

S 16 - - Products 22a + 22b in C_6D_6, with ferrocene standard, yield 84% (6:94). [75°C, 0.5 h]

Table 3, entry 7 – (24a/b) (E/Z)-2-(2,2-dimethylpropylidene)tetrahydrofuran

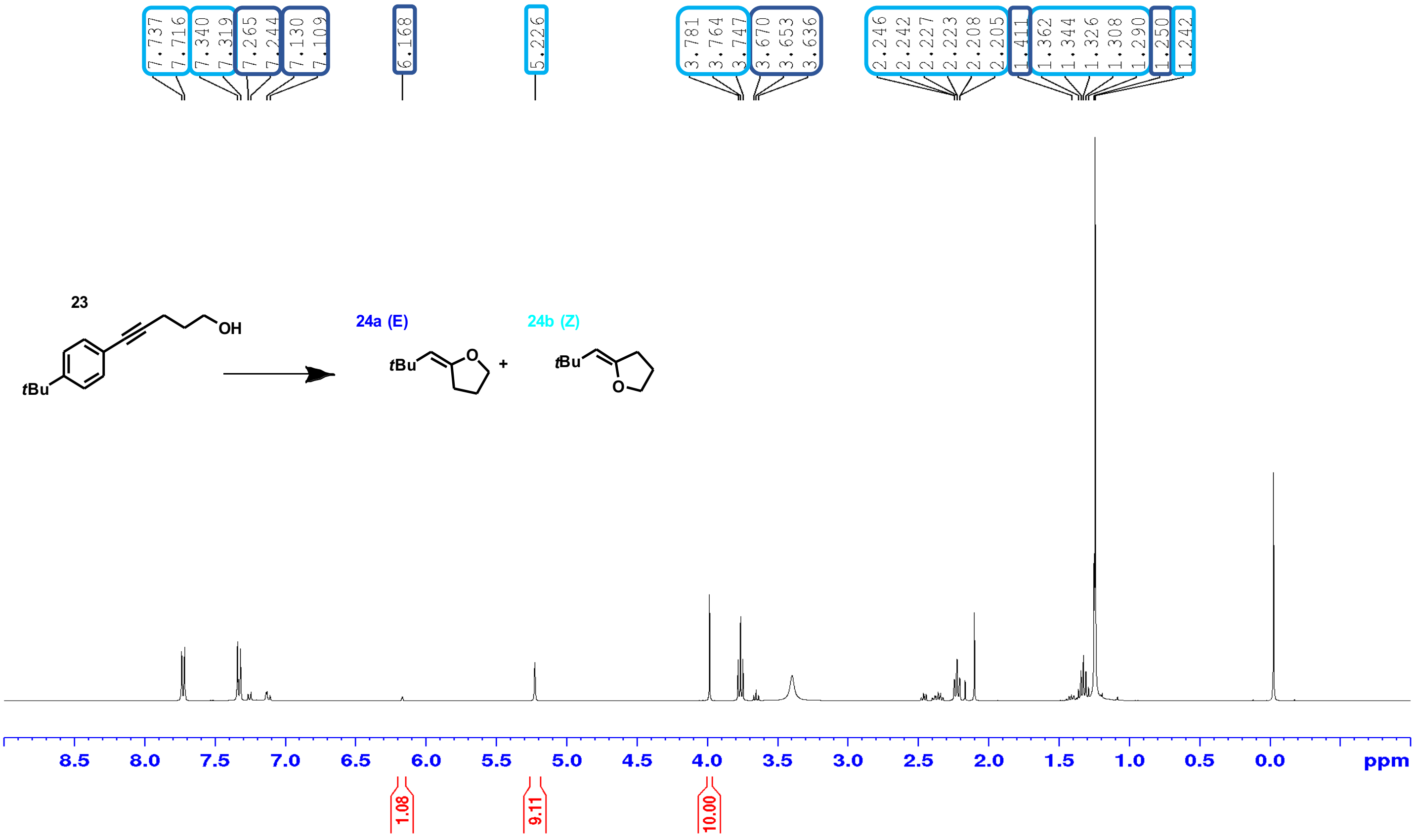

S17 - Products 24a + 24b in C_6D_6, with ferrocene standard, yield 87% (11:89). [75°C, 6 h]

Synthesis & characterisation of new alkynol and cyclic products

General procedure for synthesis and isolation

Compounds (**11**, **13**, **15**, **17**, **19** and **21**) were prepared by Sonogashira cross-coupling[S8] from 4-pentynol and the relevant Iodoaryl compound. For the example of **11**:

49 mg $Pd(PPh_3)_4$ (0.04 mmol/0.01 eq.) and 15 mg Cu(I)I (0.08 mmol/0.02 eq.) were added to a stirring mixture of 0.56 mL iodobenzene (5 mmol/1.25 eq.), 0.37 mmol 4-pentynol (4 mmol/1 eq.) and 11 mL triethylamine (80 mmol/20 eq.) in 1 mL THF and left to stir overnight at rt. The resultant product mixture was filtered and concentrated under reduced pressure yielding the crude product mixture.

The crude product mixture was purified by silica column chromatography eluted by hexane:ethyl acetate on a 10:1 to 3:1 gradient. The appropriate fraction was concentrated under reduced pressure to yield the purified product (typical yield 80%).

Compounds (**14a**, **16a**, **18a**, **20a**, **22a**) were formed by catalytic cyclisation and isolated by silica column chromatography eluted by hexane:ethyl acetate [or hexane:diethyl ether (**20a**)] on a 10:1 to 3:1 gradient. The appropriate fraction was concentrated under reduced pressure to yield the purified product.

(13) 5-(4-methoxyphenyl)pent-4-yn-1-ol

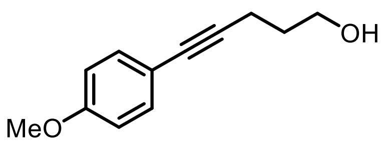

Pale-yellow waxy solid
mp. : 38-40°C / R_f : (Hx:EtOAc 2:1) 0.21, (1:1) 0.38

NMR: **^{1}H** (400.1 MHz, $CDCl_3$) δ 7.32 (2H, td, J = 9.0, 2.3 Hz, *Ar*), 6.1 (2H, td, J = 9.0, 2.2 Hz, *Ar*), 3.81 (2H, t, J = 6.2 Hz, C*H_2*OH), 3.78 (3H, s, OC*H_3*), 2.52 (2H, t, J = 6.9 Hz, CCC*H_2*), 1.85 (2H, quin, J = 6.5 Hz, C*H_2*CH_2OH), 1.70 (1H, s, O*H*). **^{13}C{^{1}H}** (100.7 MHz, $CDCl_3$) δ 159.2 (Ar_{ipso}), 134.0 (Ar), 116.0 (Ar_{ipso}), 114.0 (Ar), 87.8 (Ar*C*C), 81.0 (ArC*C*), 62.0 (*C*H_2OH), 55.4 (O*Me*), 31.6 (*C*H_2CH_2OH), 16.1 (CC*C*H_2).
MS (NSI-FTMS): 192.1097 $(M+H)^+$ - $C_{12}H_{15}O_2$

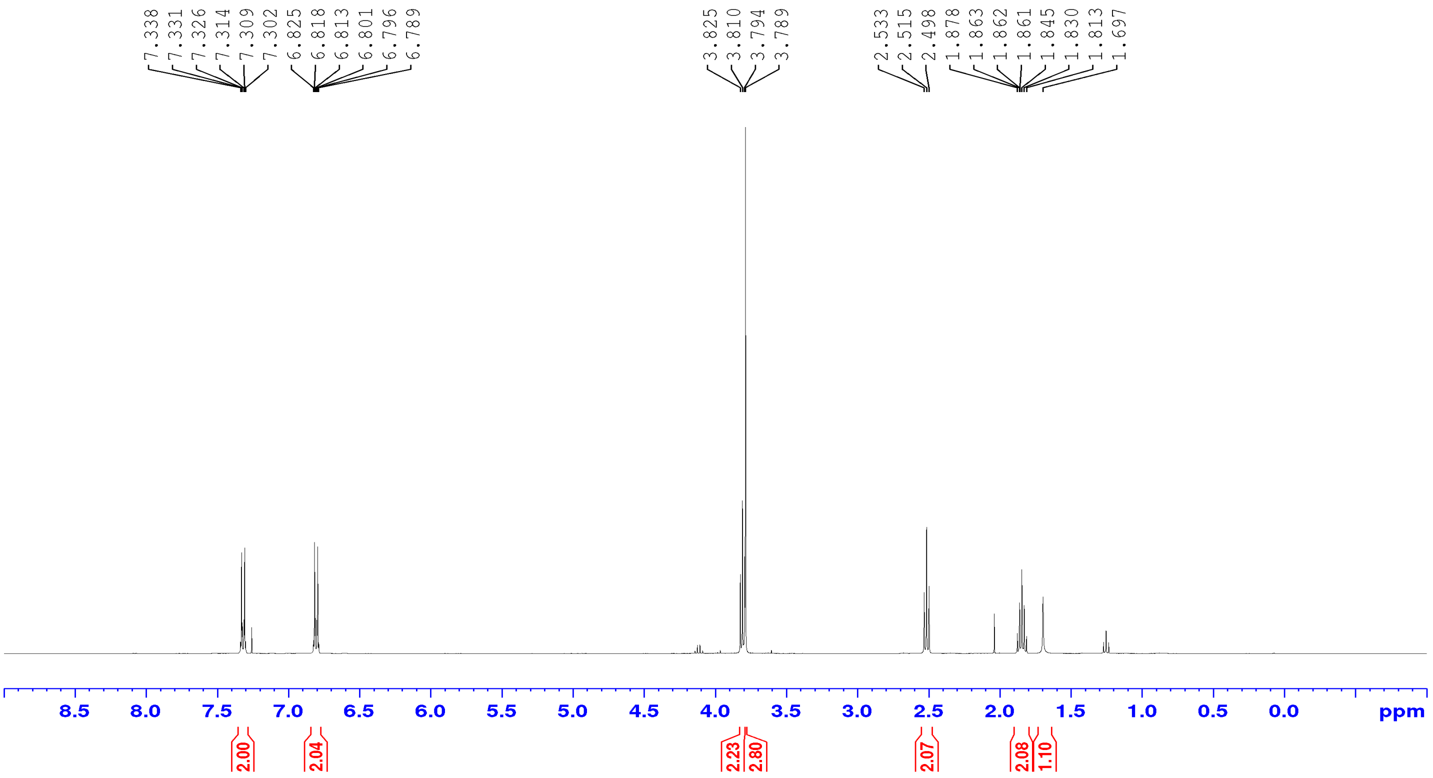

S18 – ^{1}H spectrum of 5-(4-methoxyphenyl)pent-4-yn-1-ol in $CDCl_3$

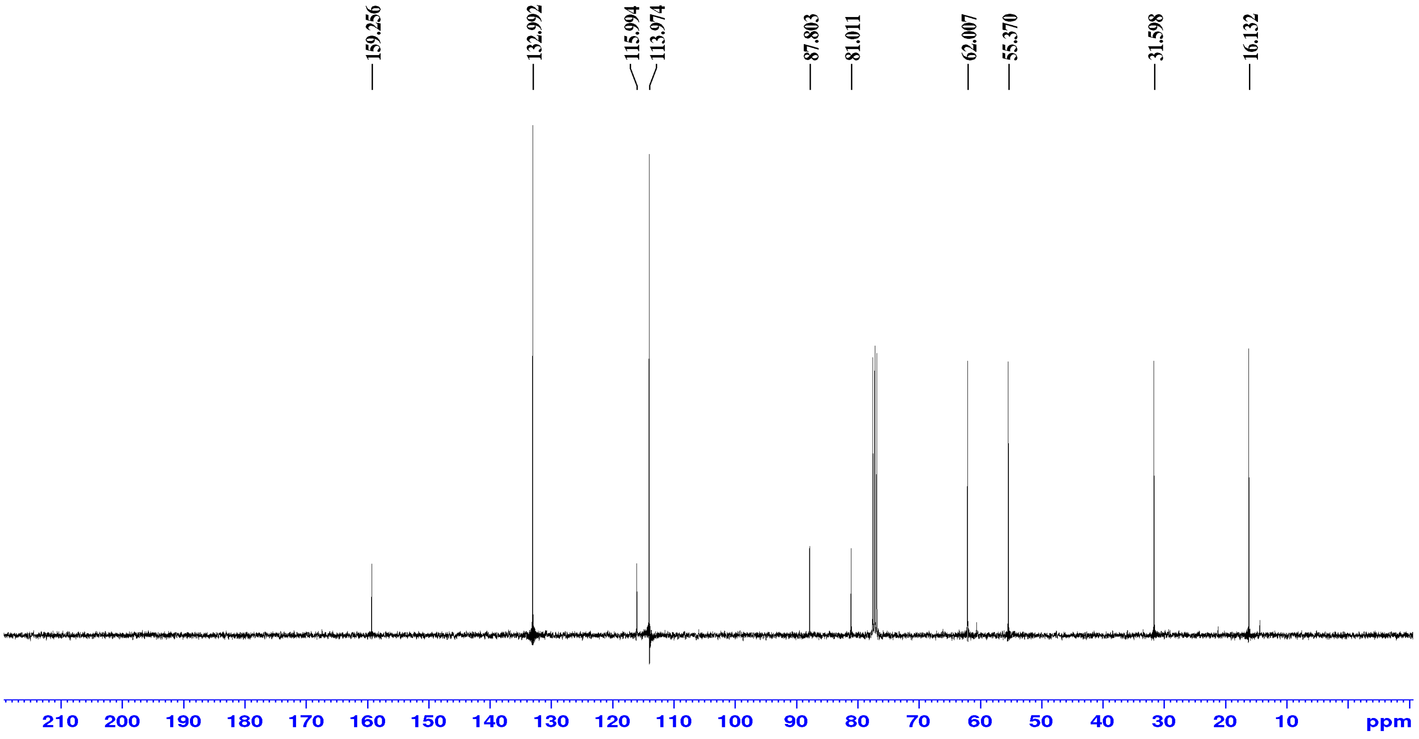

S19 - ^{13}C{^{1}H} spectrum of 5-(4-methoxyphenyl)pent-4-yn-1-ol in $CDCl_3$

(14a) (Z)-2-(4-methoxybenzylidene)tetrahydrofuran

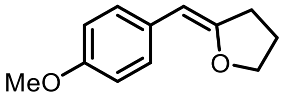

White solid

mp. : 53-55°C / R_f : (Hx:EtOAc 2:1) 0.54, (1:1) 0.61

NMR: **^{1}H** (400.1 MHz, $CDCl_3$) δ 7.48 (2H, dt, J = 8.9, 2.2 Hz, *Ar*), 6.83 (2H, dt, J = 8.9, 2.2 Hz, *Ar*),5.20 (1H, s, CC*H*), 4.30 (2H, t, J = 6.7 Hz, C*H*$_2$O), 3.79 (3H, s, O*Me*), 2.70 (2H, dt, J = 7.5, 1.4 Hz, CC*H*$_2$), 2.0 (2H, quin, J = 7.0 Hz, OCH$_2$C*H*$_2$). **^{13}C{^{1}H}** (100.7 MHz, $CDCl_3$) δ 156.9 (Ar$_{ipso}$), 155.9 (O*C*CH), 130.0 (Ar$_{ipso}$), 128.3 (Ar), 113.8 (Ar), 96.4 (C*C*H), 72.1 (O*C*H$_2$), 55.4 (O*Me*), 30.9 (OCH$_2$*C*H$_2$), 24.6 (CC*C*H$_2$).

MS (EI/TOF-MS): 190.0994 (M) - $C_{12}H_{14}O_2$

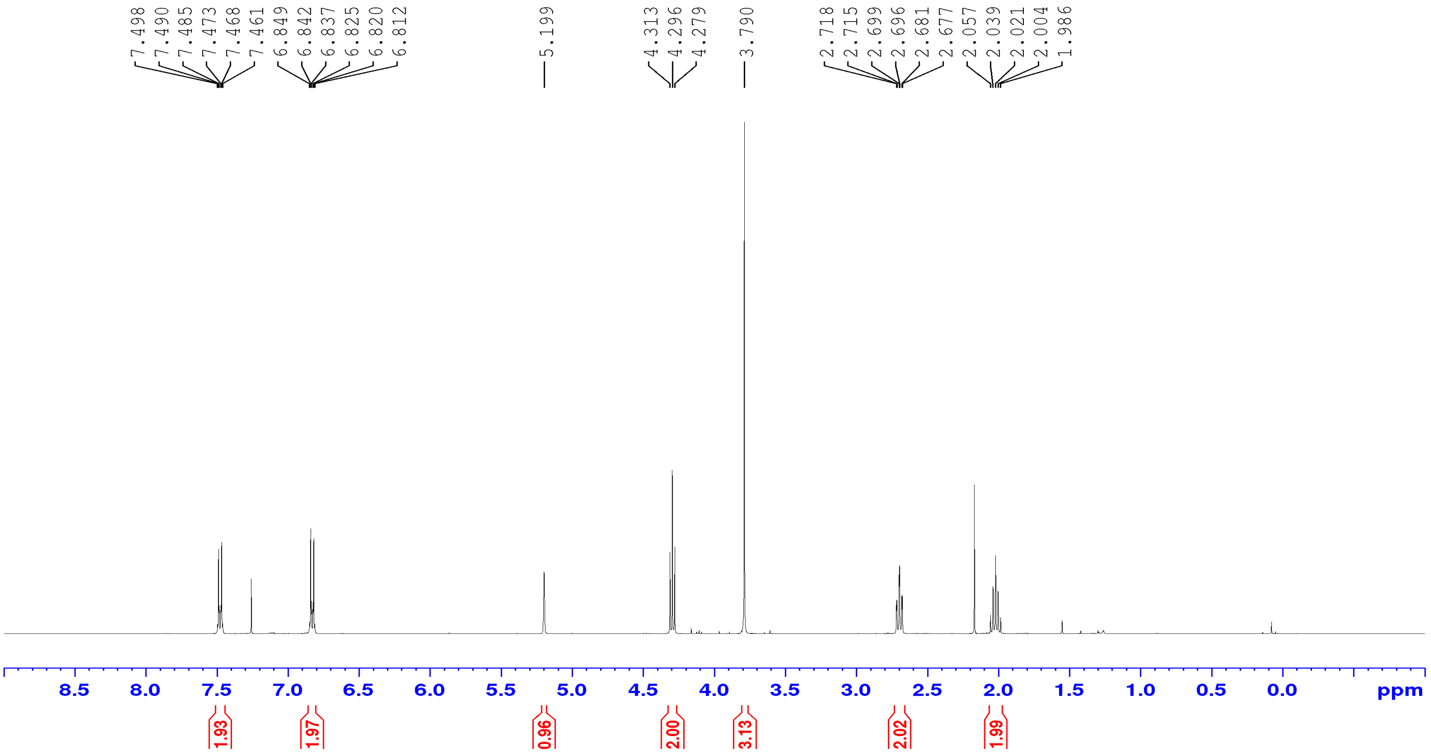

S20 - ^{1}H spectrum (Z)-2-(4-methoxybenzylidene)tetrahydrofuran in $CDCl_3$

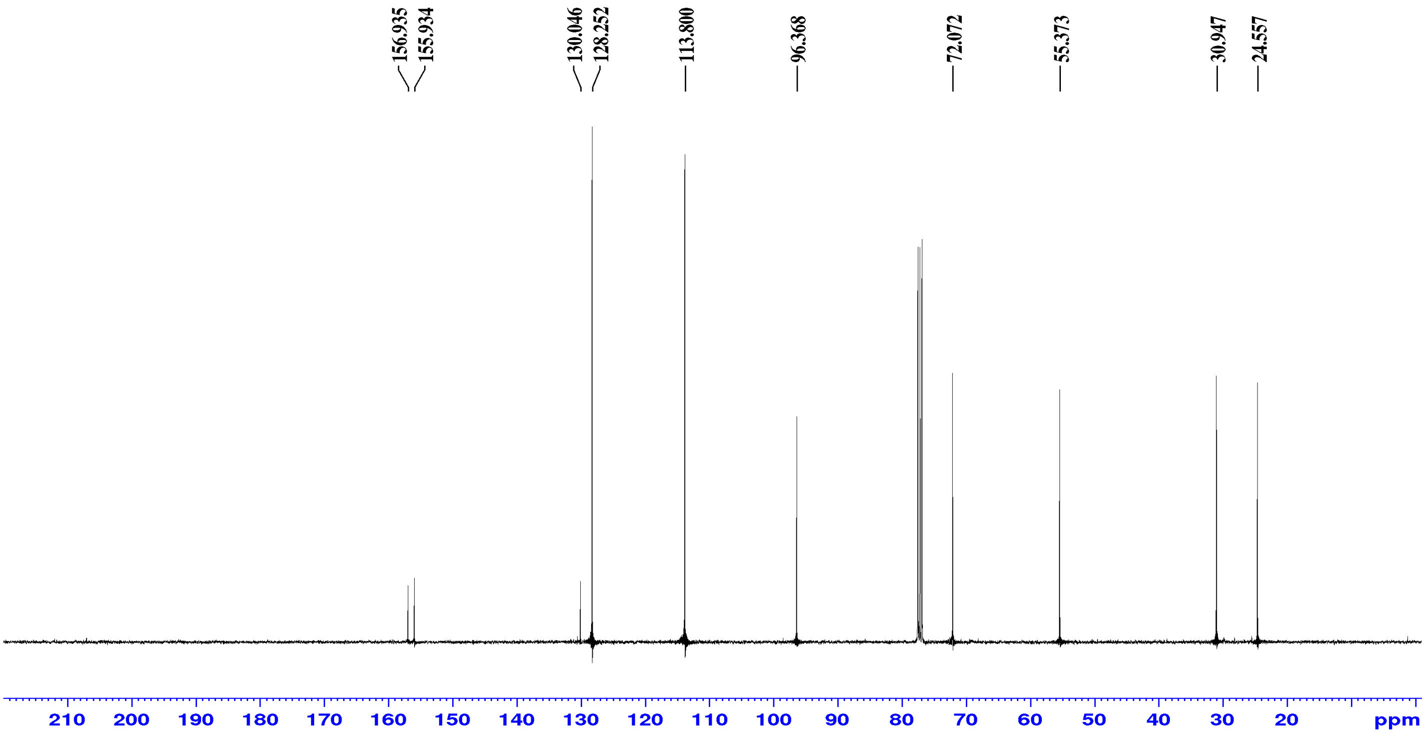

S21 - ^{13}C{^{1}H} spectrum (Z)-2-(4-methoxybenzylidene)tetrahydrofuran in $CDCl_3$

(15) 5-(4-chlorophenyl)pent-4-yn-1-ol

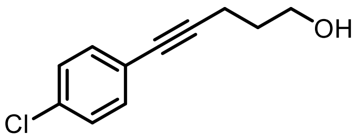

Pale-yellow waxy solid

mp. : 32-34°C / R_f : (Hx:EtOAc 2:1) 0.23, (1:1) 0.39

NMR: **^{1}H** (400.1 MHz, $CDCl_3$) δ 7.30 (2H, dt, J = 8.6, 2.1 Hz, *Ar*), 7.24 (2H, J = 8.6, 2.1 Hz, *Ar*), 3.79 (2H, t, J = 6.1 Hz, *C*H_2OH), 2.52 2H, t, J = 7.0 Hz, CCC*H_2*), 1.93 (1H, s, O*H*), 1.84 (2H, quin, J = 6.6 Hz, C*H_2*CH_2OH). **^{13}C{^{1}H}** (100.7 MHz, $CDCl_3$) δ 133.7 (Ar_{ipso}), 132.9 (Ar), 128.6 (Ar), 122.3 (Ar_{ipso}), 90.5 (ArC*C*), 80.1 (Ar*C*C), 61.7 (*C*H_2OH), 31.3 (CH_2*C*H_2OH), 16.0 (*C*$H_2$$CH_2$OH).

MS (ASAP/TOF-MS): 197.0550 (M+H) – $C_{11}H_{12}OCl$

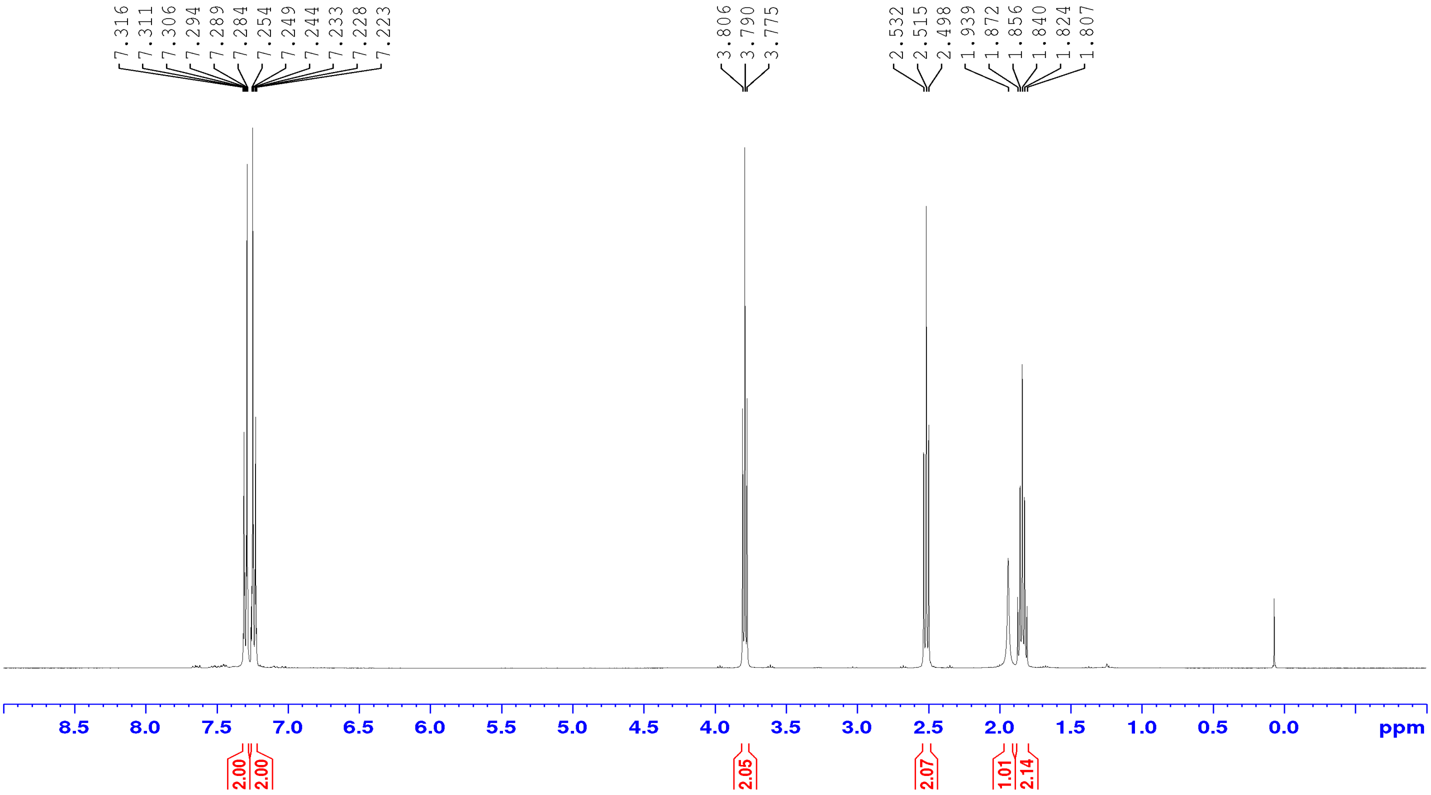

S22 – ^{1}H spectrum of 5-(4-chlorophenyl)pent-4-yn-1-ol in $CDCl_3$

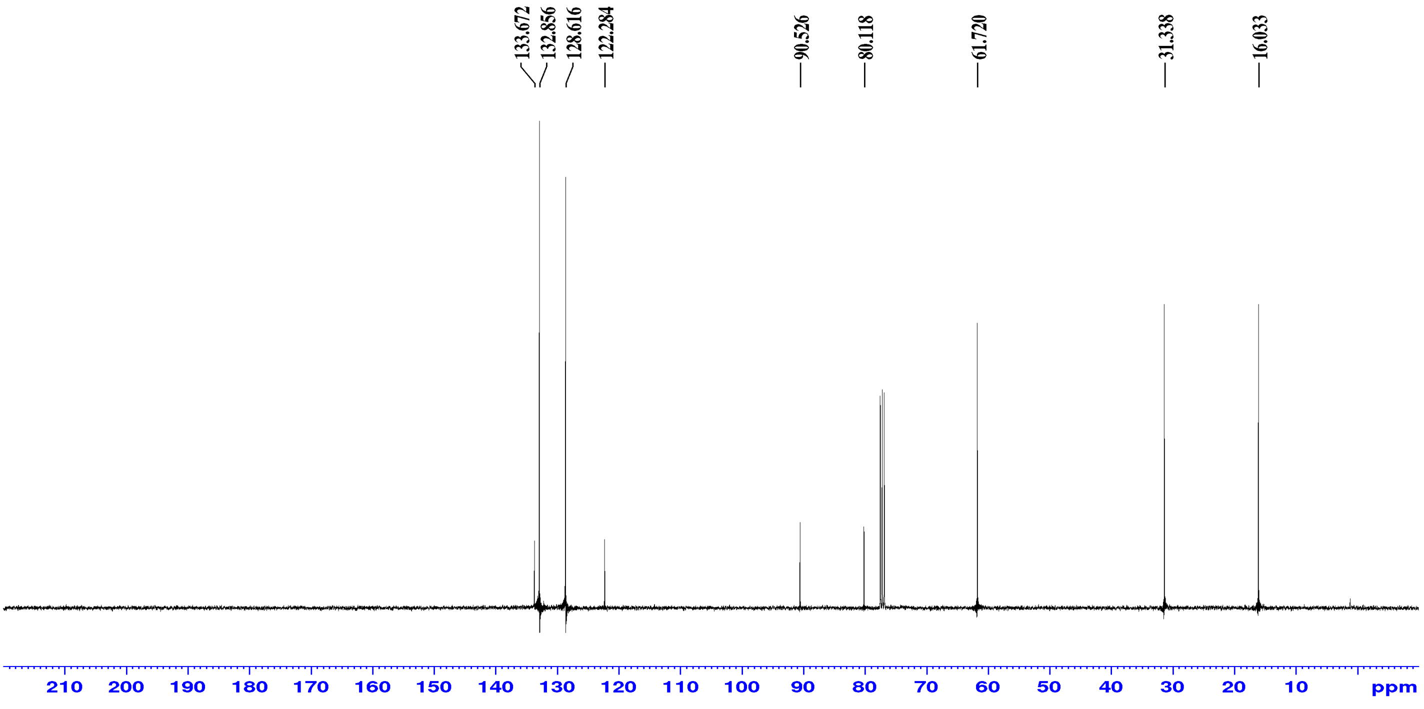

S23 – ^{13}C{^{1}H} spectrum of 5-(4-chlorophenyl)pent-4-yn-1-ol in $CDCl_3$

(16a) (Z)-2-(4-cholorobenzylidene)tetrahydrofuran

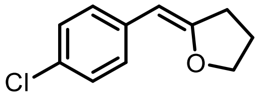

Pale-yellow oil

R_f : (Hx:EtOAc 2:1) , (1:1)

NMR: **^{1}H** (400.1 MHz, $CDCl_3$) δ 7.46 (2H, d, J = 8.6 Hz, *Ar*), 7.21(2H, d, J = 8.6 Hz, *Ar*), 5.20 (1H, s, CC*H*), 4.33 (2H, t, J = 6.7 Hz, C*H*$_2$O), 2.716 (2H, td, J = 6.7, 1.1 Hz, CC*H*$_2$), 2.04 (2H, quin, J = 7.2 Hz, OCH_2C*H*$_2$). **^{13}C{^{1}H}** (100.7 MHz, $CDCl_3$) δ 156.9 (O*C*CH), 135.0 (Ar_{ipso}), 129.2 (Ar_{ipso}), 127.7 (Ar), 95.4 (C*C*H), 71.9 (O*C*H$_2$), 30.6 (OCH_2*C*H$_2$), 23.8 (CC*C*H$_2$).

MS (CI/TOF-MS): 195.0571$(M+H)^+$ - $C_{11}H_{12}O_1Cl$

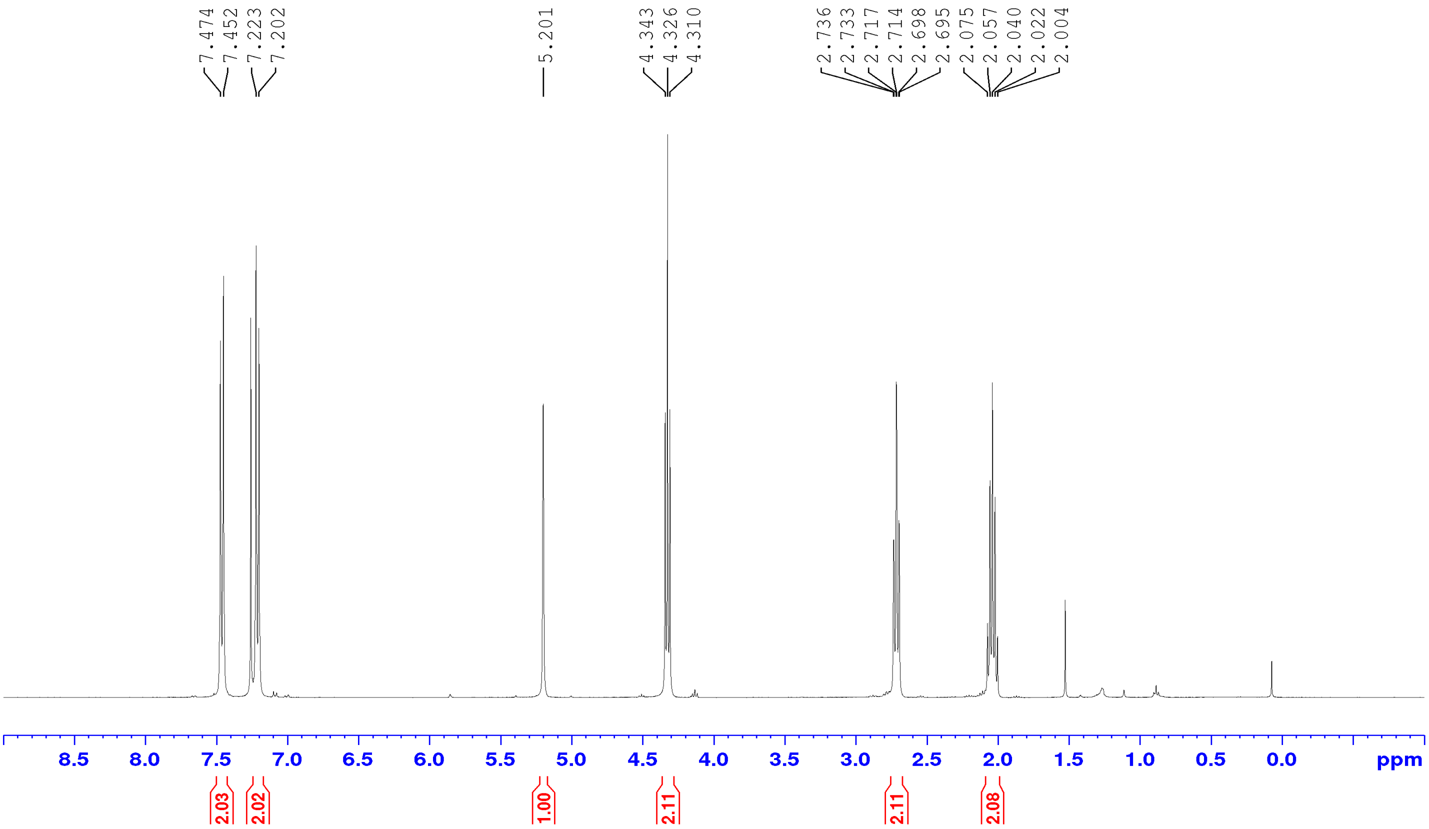

S24 - ^{1}H spectrum (Z)-2-(4-cholorobenzylidene)tetrahydrofuran in $CDCl_3$

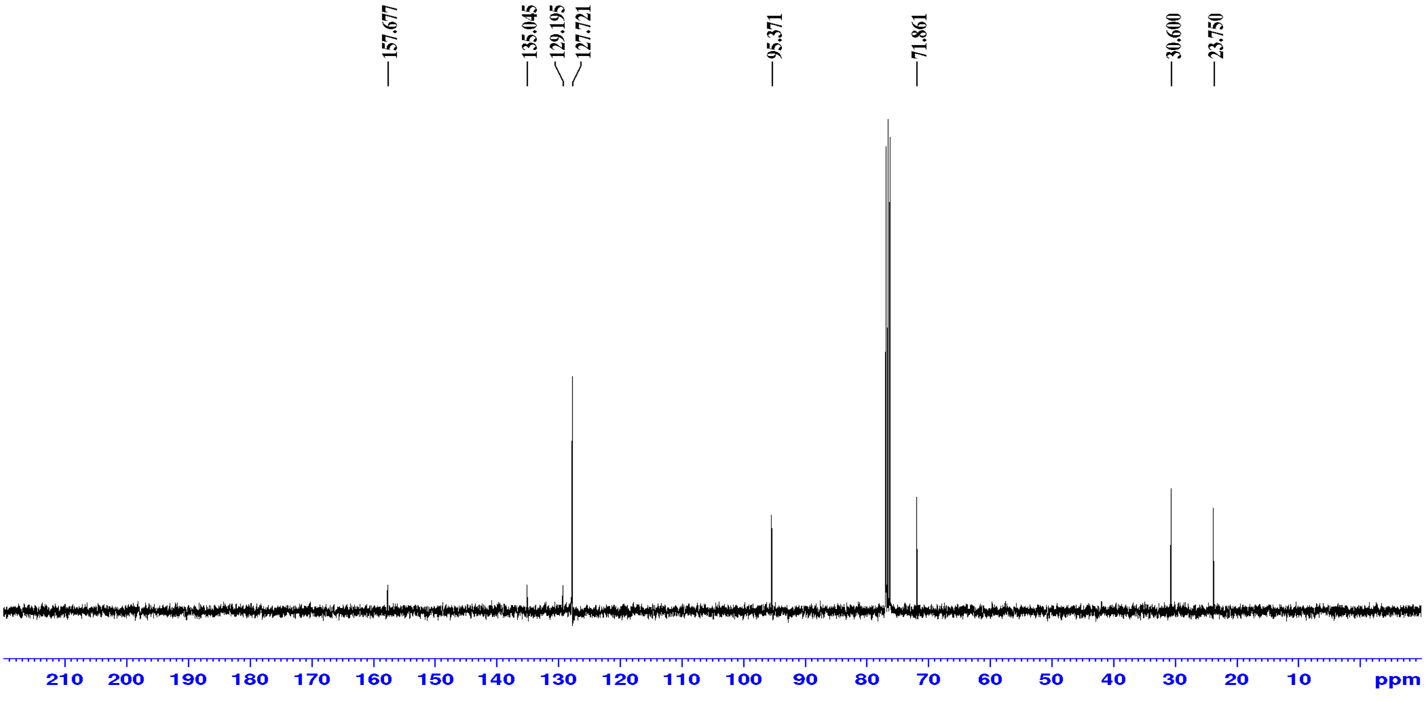

S25 - ^{13}C{^{1}H} spectrum (Z)-2-(4-cholorobenzylidene)tetrahydrofuran in $CDCl_3$

(17) 5-(3-fluorophenyl)pent-4-yn-1-ol

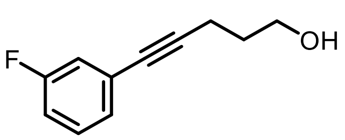

Red oil

R_f : (Hx:EtOAc 2:1) 0.55, (1:1) 0.58

NMR: **^{1}H** (400.1 MHz, $CDCl_3$) δ 7.23 (1H, td, J = 8.0, 5.9 Hz, *Ar*), 7.06 (1H, dt, J = 7.7, 1.2 Hz, *Ar*), 7.08 (1H, ddd, J = 9.6, 2.5, 1.4 Hz, *Ar*), 6.97 (1H, tdd, J = 8.5, 2.6, 1.1 Hz, *Ar*), 3.81 (2H, t, J = 6.1 Hz, CH_2OH), 2.53 (2H, t, J = 6.9 Hz, $CCCH_2$), 1.86 (2H, quin, J = 6.6 Hz, CH_2CH_2OH). **^{13}C{^{1}H}** (100.7 MHz, $CDCl_3$) δ 162.5 (d, J = 144.8 Hz, Ar_{ipso}), 129.9 (d, J = 8.8 Hz, Ar), 127.5 (d, J = 3.1 Hz, Ar), 125.7 (d, J = 9.4 Hz, Ar_{ipso}), 177.8 (d, J = 22.4 Hz, Ar), 115.1 (d, J = 21.0 Hz, Ar), 90.6 (ArC*C*), 80.2 (d, J = 3.3 Hz, Ar*C*C), 61.8 (CH_2OH), 31.4 (CH_2CH_2OH), 16.1 (CH_2CH_2OH). **^{19}F** (128.4 MHz, $CDCl_3$) δ -113.3 (td, J = 9.3, 6.0 Hz).

MS (ASAP/TOF-MS): 178.0794 (M+H) – $C_{11}H_{11}OF$

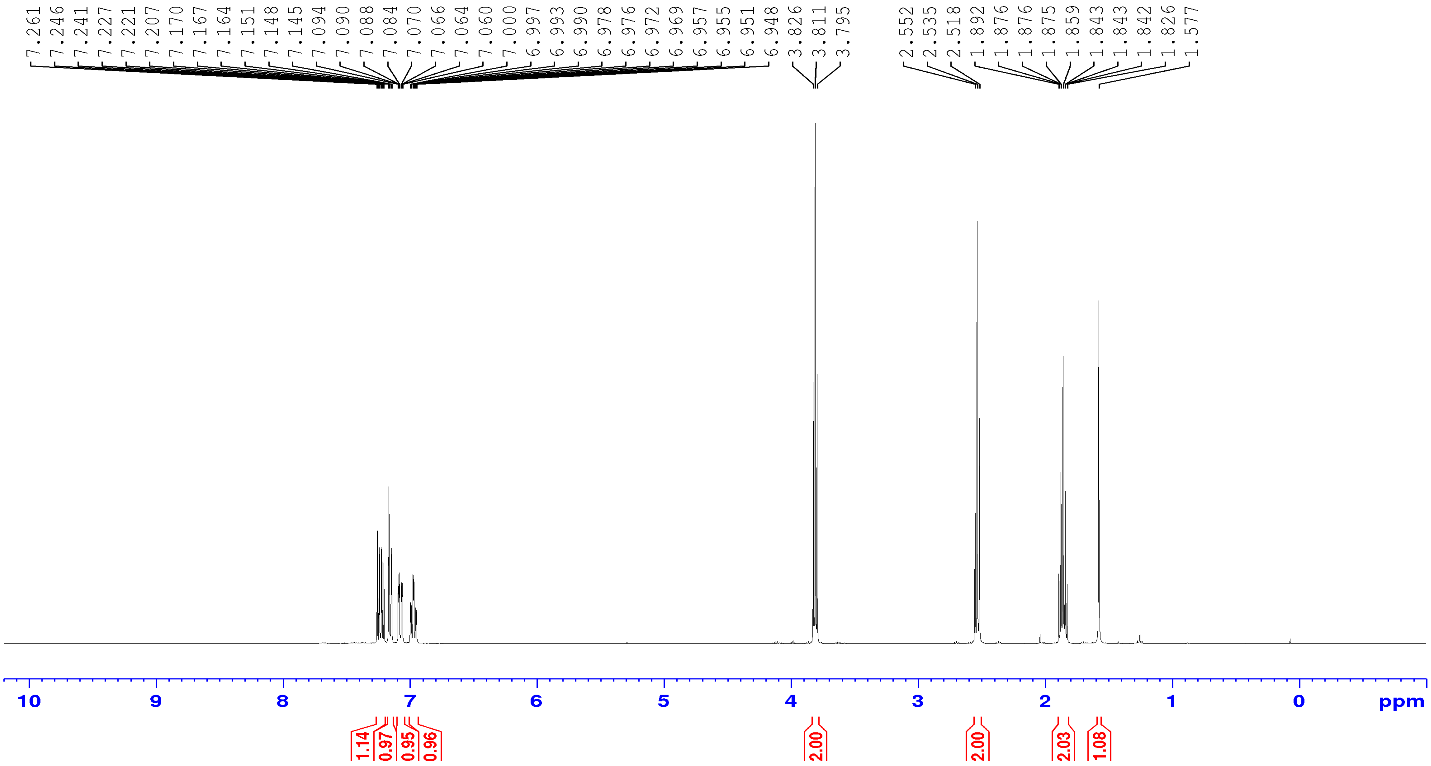

S27 - ^{1}H spectrum of 5-(3-fluorophenyl)pent-4-yn-1-ol in $CDCl_3$

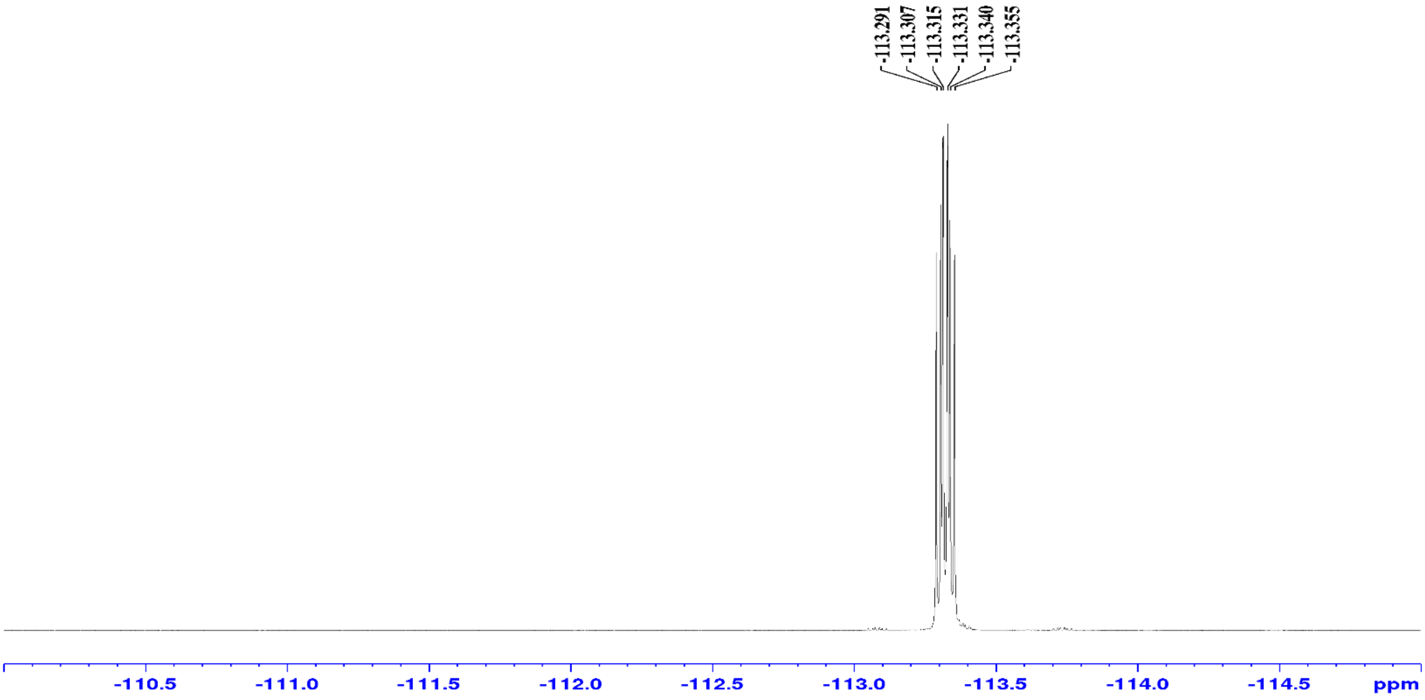

S26 – ^{19}F spectrum of 5-(3-fluorophenyl)pent-4-yn-1-ol in $CDCl_3$

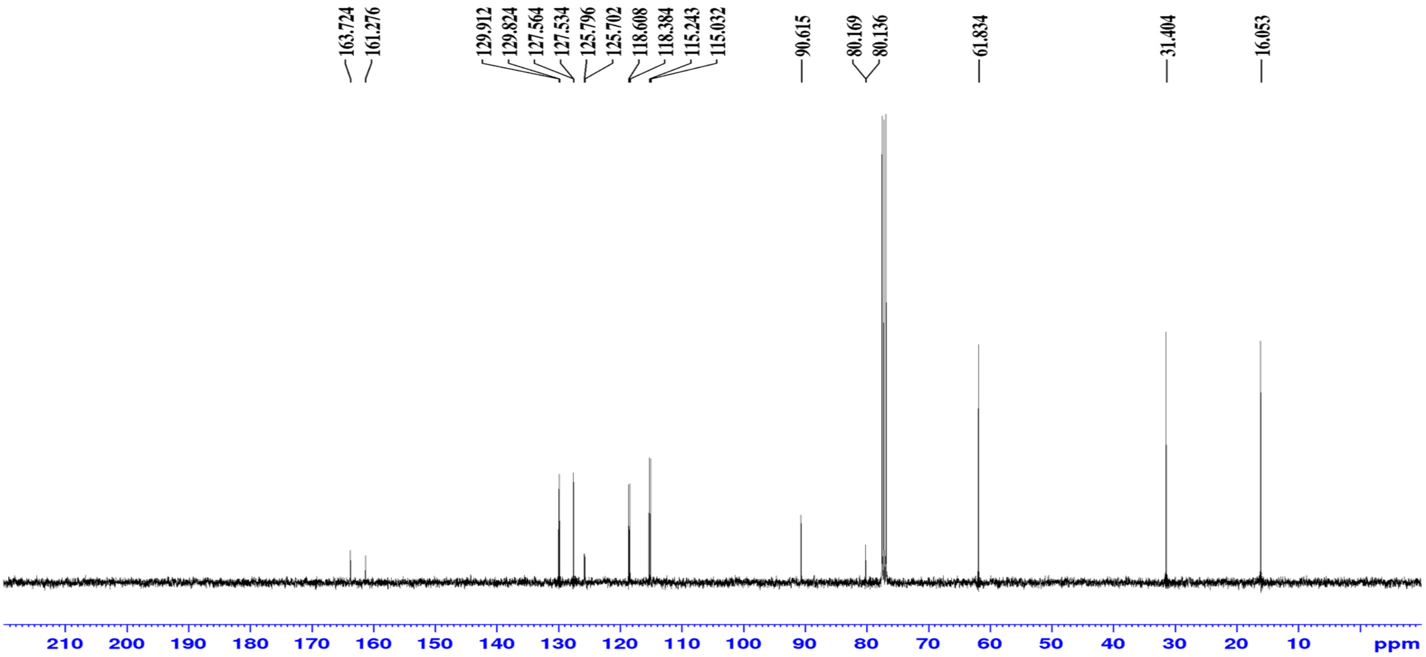

S28 – $^{13}C\{^{1}H\}$ spectrum of 5-(3-fluorophenyl)pent-4-yn-1-ol in $CDCl_3$

(18a) (Z)-2-(3-fluorobenzylidene)tetrahydrofuran

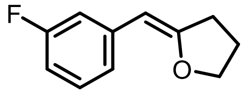

Pale yellow oil

R_f : (Hx:EtOAc 2:1) 0.52, (1:1) 0.60

NMR: **^{1}H** (400.1 MHz, $CDCl_3$) δ 7.37 (1H, m, *Ar*), 7.20 (1H, m, *Ar*), 6.76 (1H, m, *Ar*), 5.23 (1H, s, CC*H*), 4.34 (2H, t, J = 6.8 Hz, OCH_2), 2.73 (2H, td, J = 7.6, 1.1 Hz, CCH_2), 2.04 (2H, quin, J = 7.1 Hz, OCH_2CH_2). **^{13}C{^{1}H}** (100.7 MHz, $CDCl_3$) δ 163.0 (d, J = 241.0 Hz, Ar_{ipso}), 158.8 (O*C*CH), 139.2 (d, J = 8.6 Hz, Ar_{ipso}), 129.3 (d, 8.8 Hz, Ar), 122.7 (Ar), 133.5 (d, J = 22.3 Hz, Ar), 111.2 (d, J = 21.4 Hz, Ar), 96.13 (C*C*H), 72.4 (OCH_2), 31.1 (OCH_2CH_2), 24.2 (CCCH_2). **^{19}F** (128.4 MHz, $CDCl_3$) δ -114.3.

MS (EI/TOF-MS): 178.0794 (M) – $C_{11}H_{11}OF$

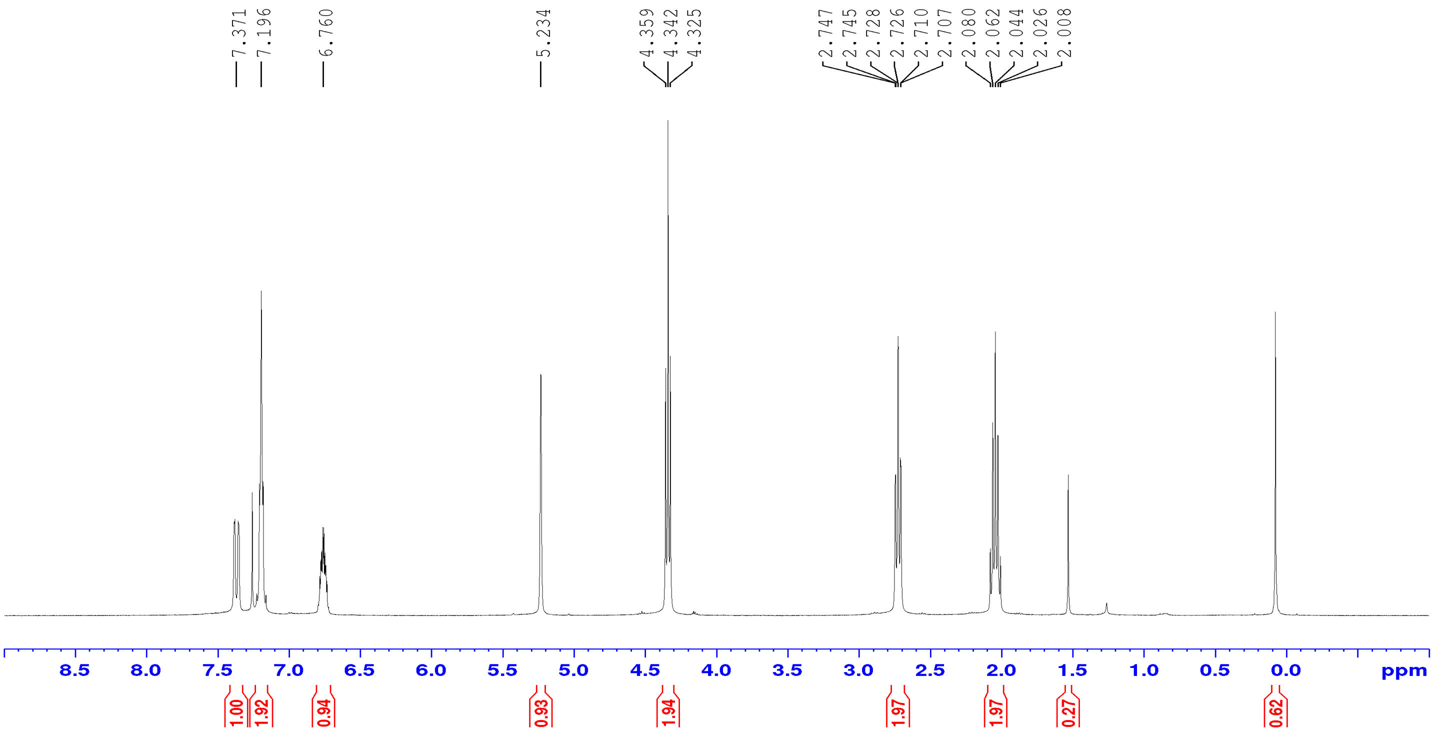

S29 – ^{1}H spectrum of (Z)-2-(3-fluorobenzylidene)tetrahydrofuran in $CDCl_3$

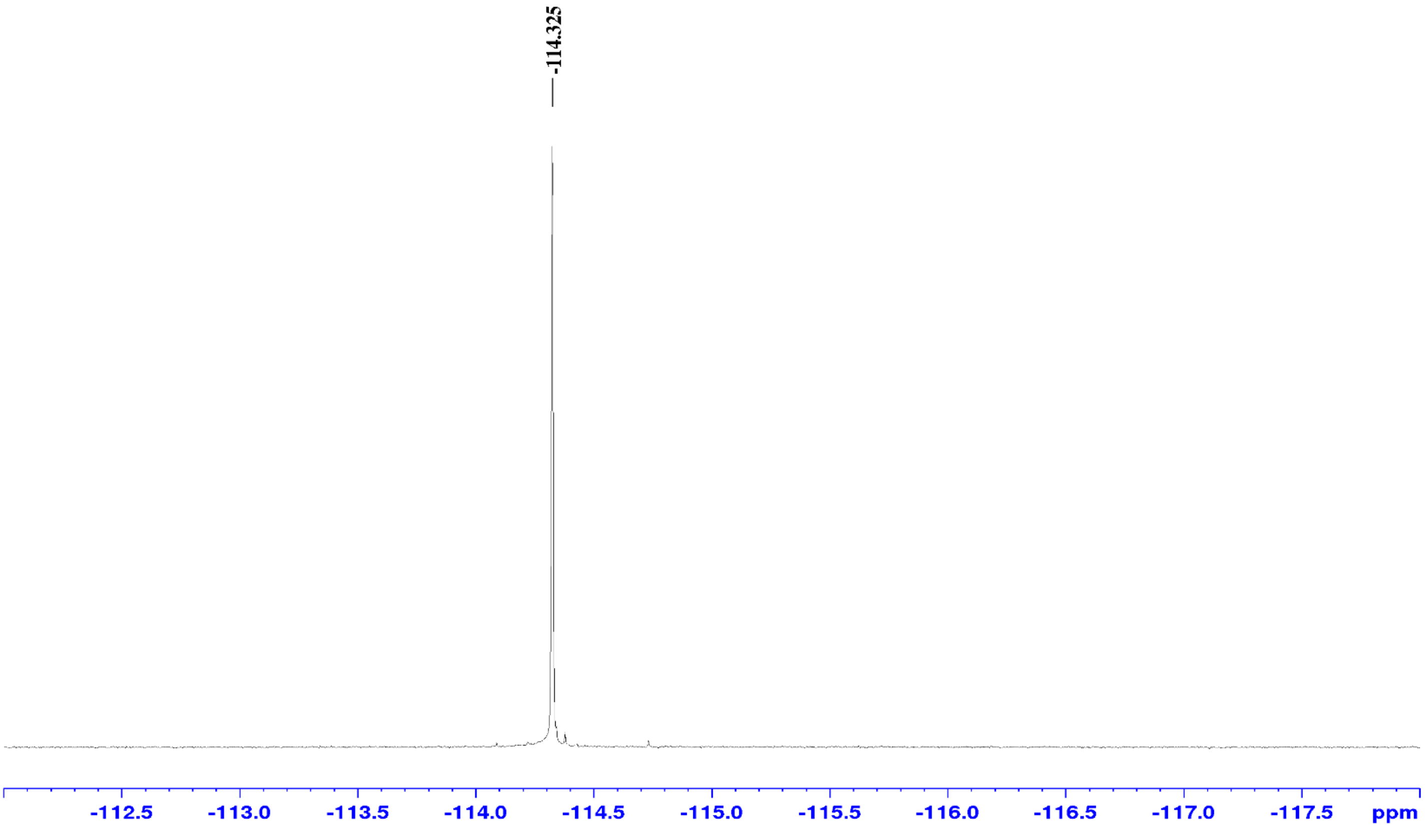

S30 – [19]f spectrum of (Z)-2-(3-fluorobenzylidene)tetrahydrofuran

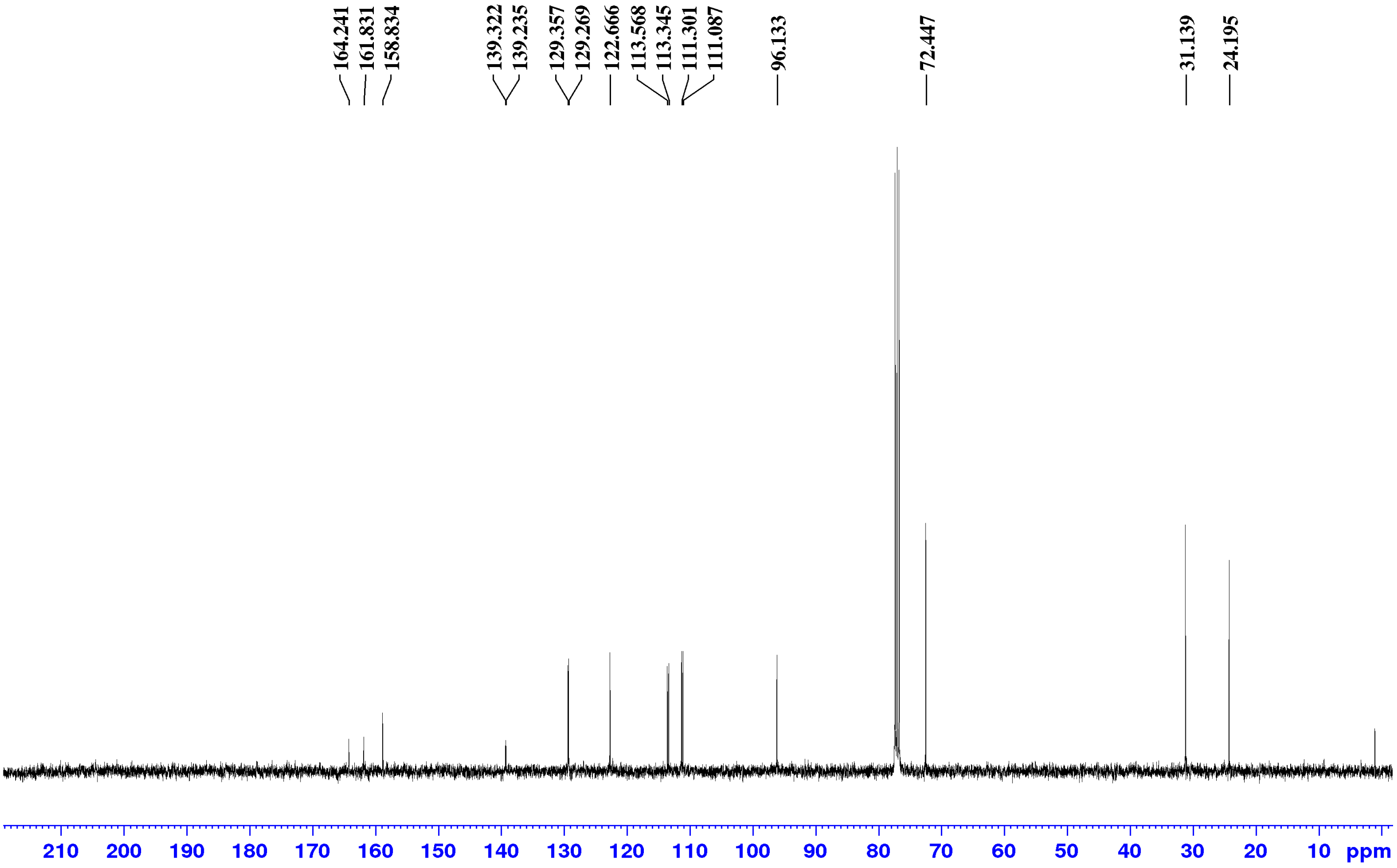

S31 - $^{13}C[^{1}H\}$ spectrum of (Z)-2-(3-fluorobenzylidene)tetrahydrofuran

(19) 5-(3,5-(trifluoromethyl)phenyl)pent-4-yn-1-ol

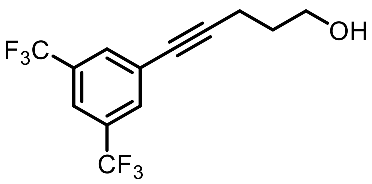

Orange oil
R_f : (Hx:EtOAc 2:1) 0.28, (1:1) 0.46

NMR: ^{1}H (400.1 MHz, $CDCl_3$) δ 7.79 (2H, s, *Ar*), 7.74 (1H, s, *Ar*), 3.80 (2H, m, C*H*$_2$OH), 2.56 (2H, t, J = 7.0 Hz, CCC*H*$_2$), 1.87 (3H, m, C*H*$_2$CH$_2$OH/O*H*). **^{13}C{^{1}H}** (100.7 MHz, $CDCl_3$) δ 131.9 (q, J = 33.6 Hz, Ar_{ipso}), 131.6 (m, Ar), 126.3 (Ar_{ipso}), 123.1 (quin, J = 272.5 Hz, *C*F$_3$), 121.1 (q, J = 3.7 Hz, Ar), 93.7 (s, ArC*C*), 78.6 (s, Ar*C*C), 61.5 (*C*H$_2$OH), 31.2 (CH$_2$*C*H$_2$OH), 16.0 (*C*H$_2$CH$_2$OH). **^{19}F** (128.4 MHz, $CDCl_3$) δ -63.3.

MS (ASAP/TOF-MS):) 297.0714 (M+H) – $C_{13}H_{11}OF_6$

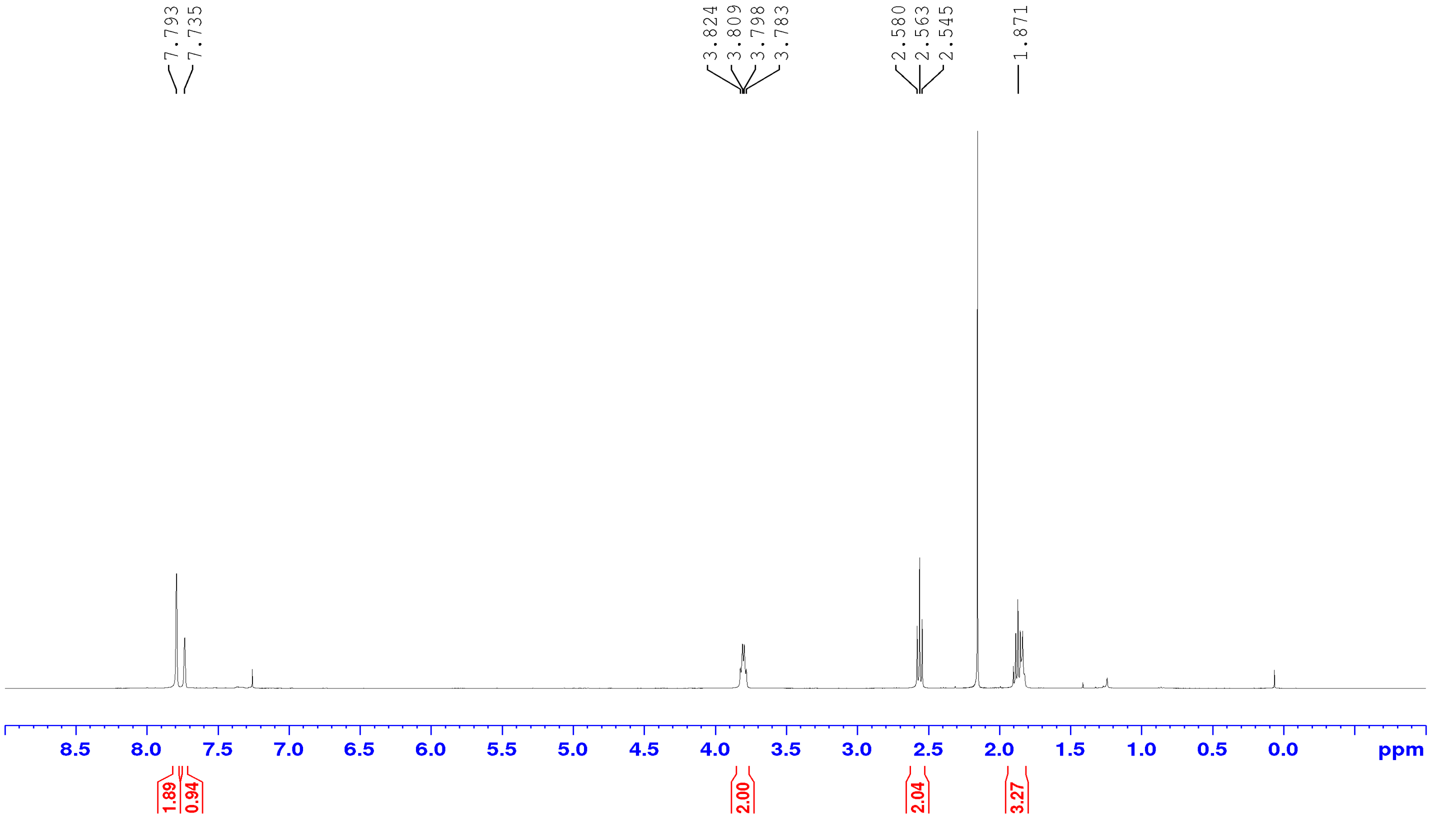

S32 – ^{1}H spectrum of 5-(3,5-(trifluoromethyl)phenyl)pent-4-yn-1-ol in $CDCl_3$

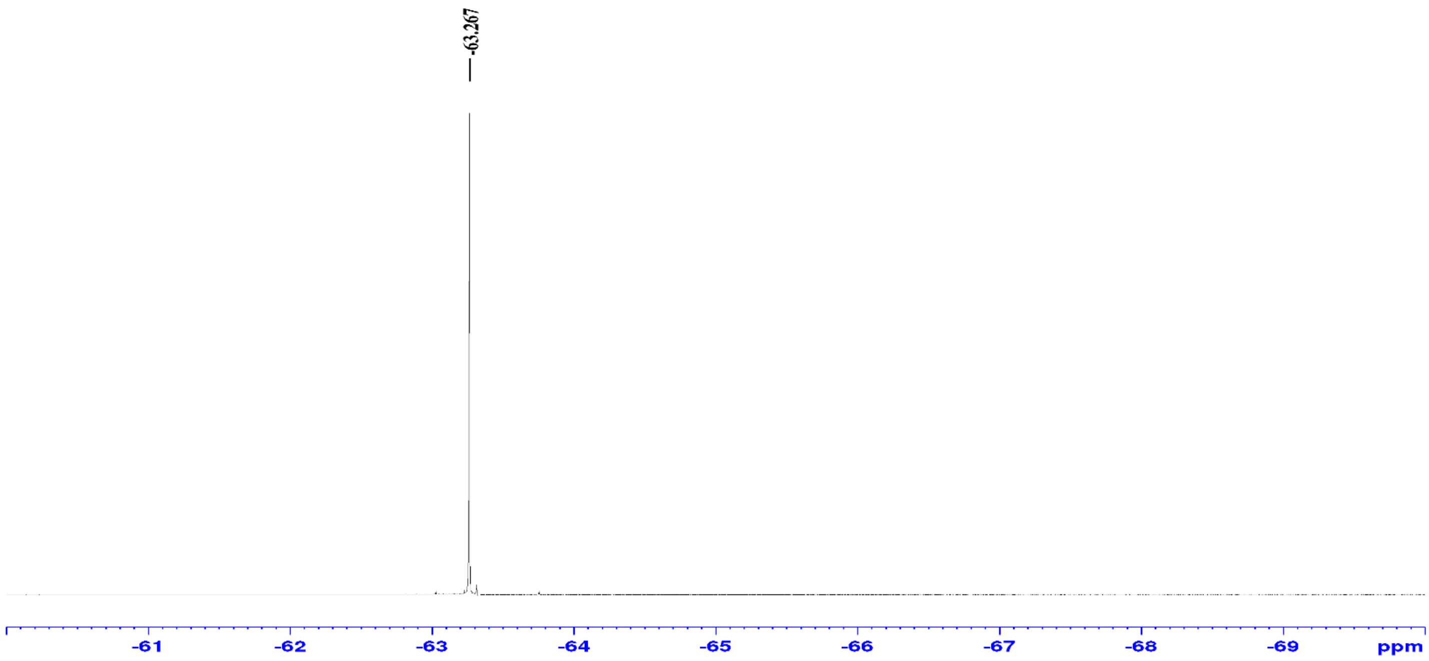

S33 – ^{19}F spectrum of 5-(3,5-(trifluoromethyl)phenyl)pent-4-yn-1-ol in $CDCl_3$

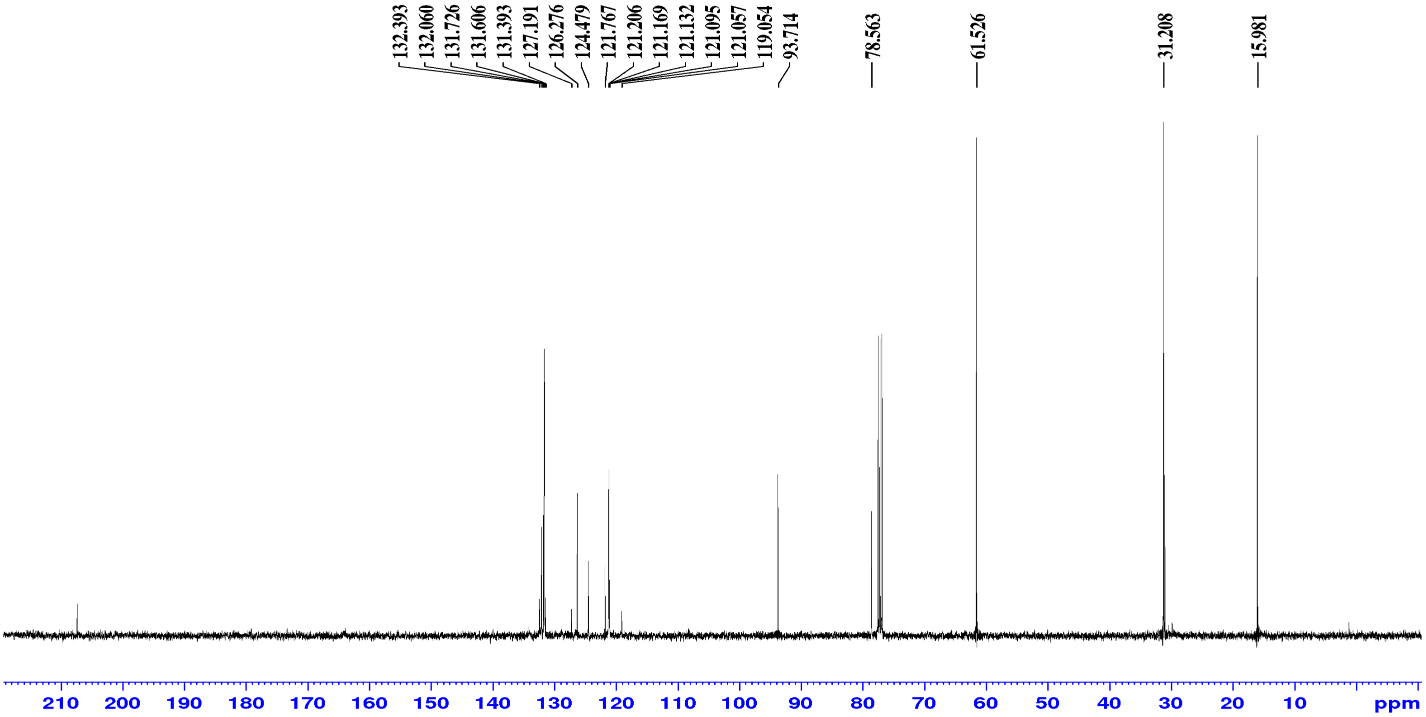

S34 – $^{13}C[^{1}H]$ spectrum of 5-(3,5-(trifluoromethyl)phenyl)pent-4-yn-1-ol in $CDCl_3$

(20a) (Z)-2-(3,5-(bistrifluoromethyl)benzylidene)tetrahydrofuran

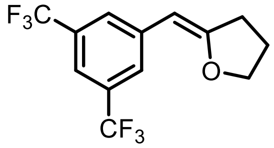

White crystalline solid

mp. : 46-48°C / R_f : (Hx:EtOAc 2:1) 0.64, (1:1) 0.78

NMR: **^{1}H** (400.1 MHz, $CDCl_3$) δ 7.95 (2H, s, *Ar*), 7.53 (1H, s, *Ar*), 5.31 (1H, s, CC*H*), 4.40 (2H, t, J = 6.8 Hz, OC*H*$_2$), 2.77 (2H, td, J = 7.6, 1.2 Hz, CC*H*$_2$), 2.08 (2H, quin, J = 7.4 Hz, OCH$_2$C*H*$_2$). **^{13}C{^{1}H}** (100.7 MHz, $CDCl_3$) δ 161.4 (O*C*CH), 139.2 (Ar$_{ipso}$), 131.3 (q, J = 32.6 Hz, Ar$_{ipso}$), 126.7 (m, Ar), 123.9 (q, J = 272.5 Hz, CF_3), 117.7 (quin, J = 3.7 Hz, Ar), 95.1 (s, ArC*C*), 73.2 (s, Ar*C*C), 61.5 (*C*H$_2$OH), 31.6 (CH$_2$*C*H$_2$OH), 24.2 (*C*H$_2$CH$_2$OH). **^{19}F** (128.4 MHz, $CDCl_3$) δ -63.0.

MS (ASAP/TOF-MS): 296.0636 (M) – $C_{13}H_{11}OF_6$

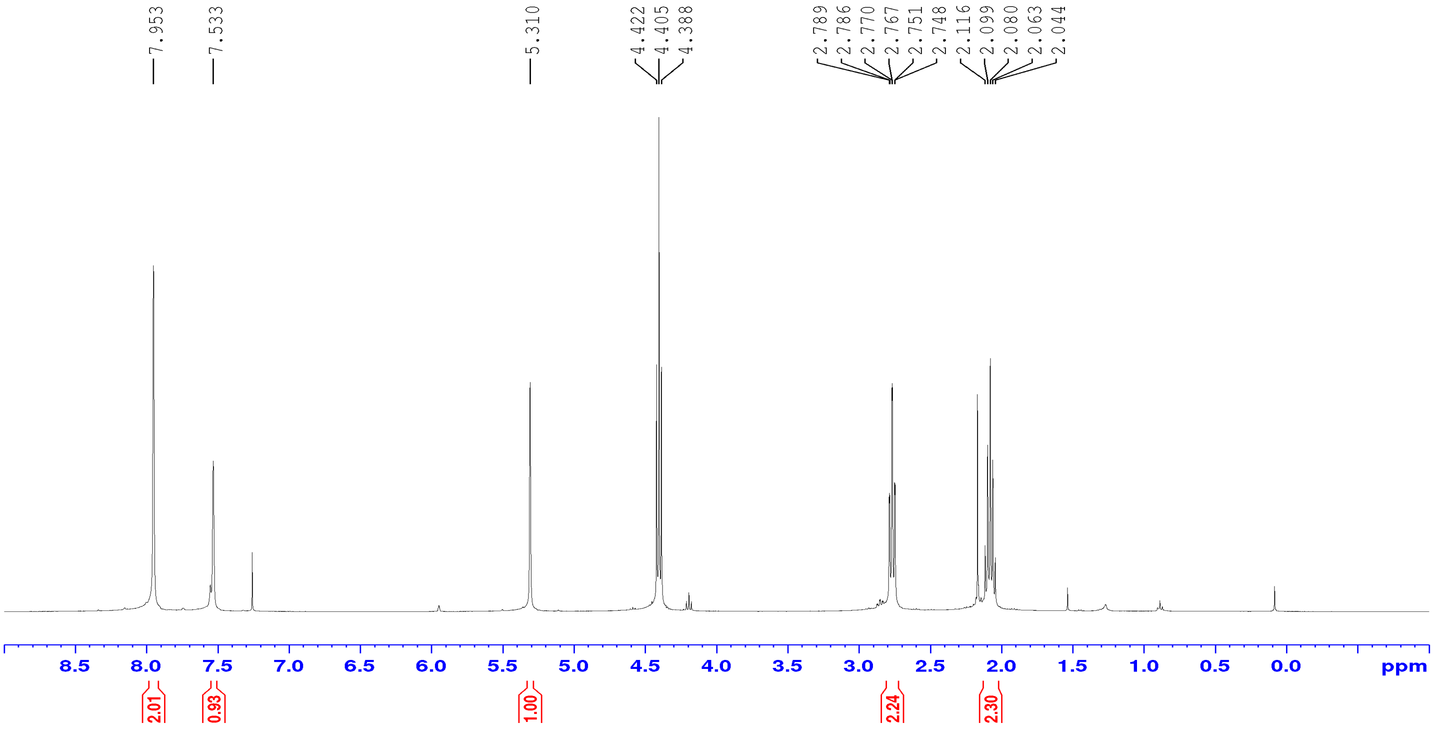

S35 - ^{1}H spectrum of (Z)-2-(3,5-(bistrifluoromethyl)benzylidene)tetrahydrofuran in $CDCl_3$

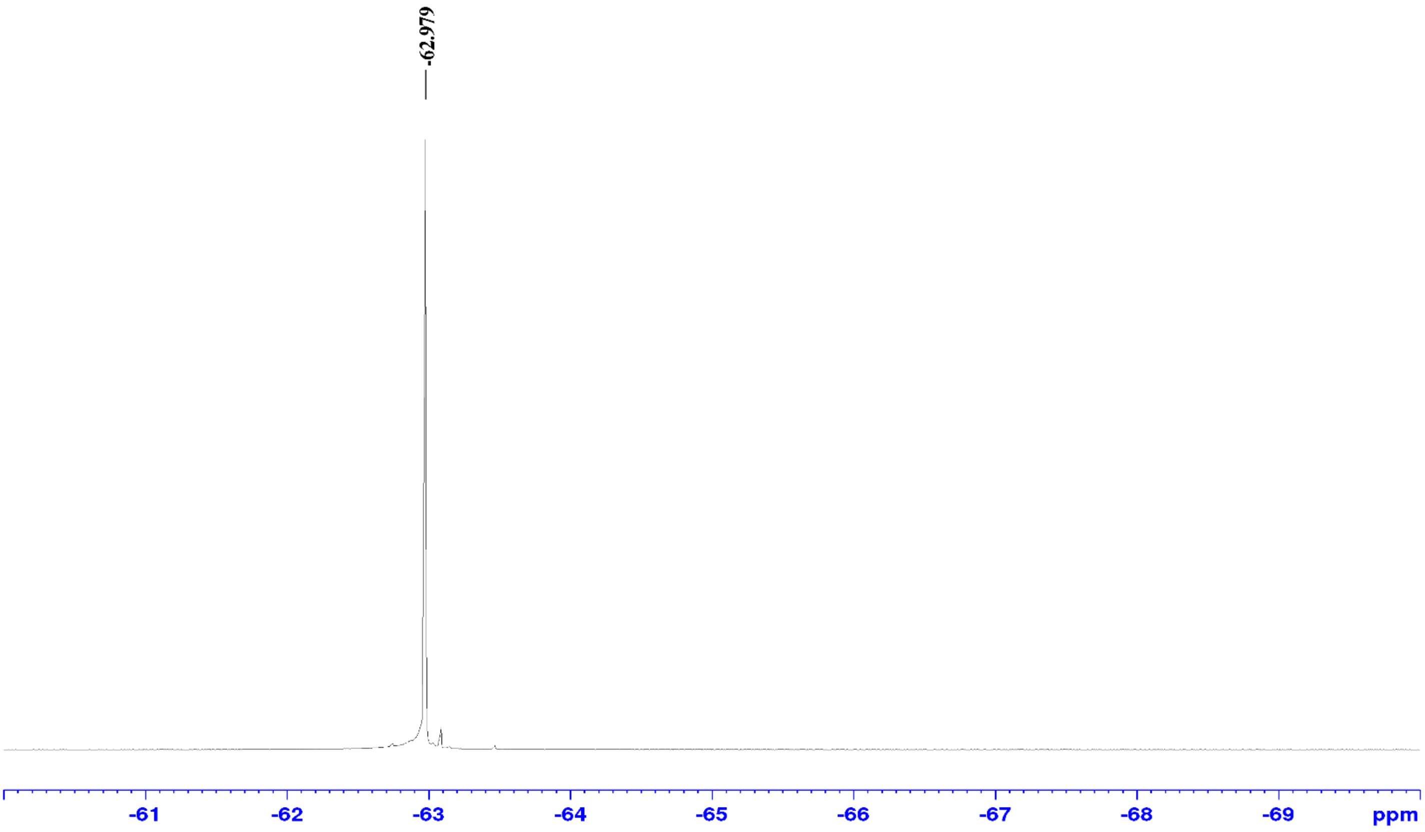

S37 - ^{19}F spectrum of (Z)-2-(3,5-(bistrifluoromethyl)benzylidene)tetrahydrofuran in $CDCl_3$

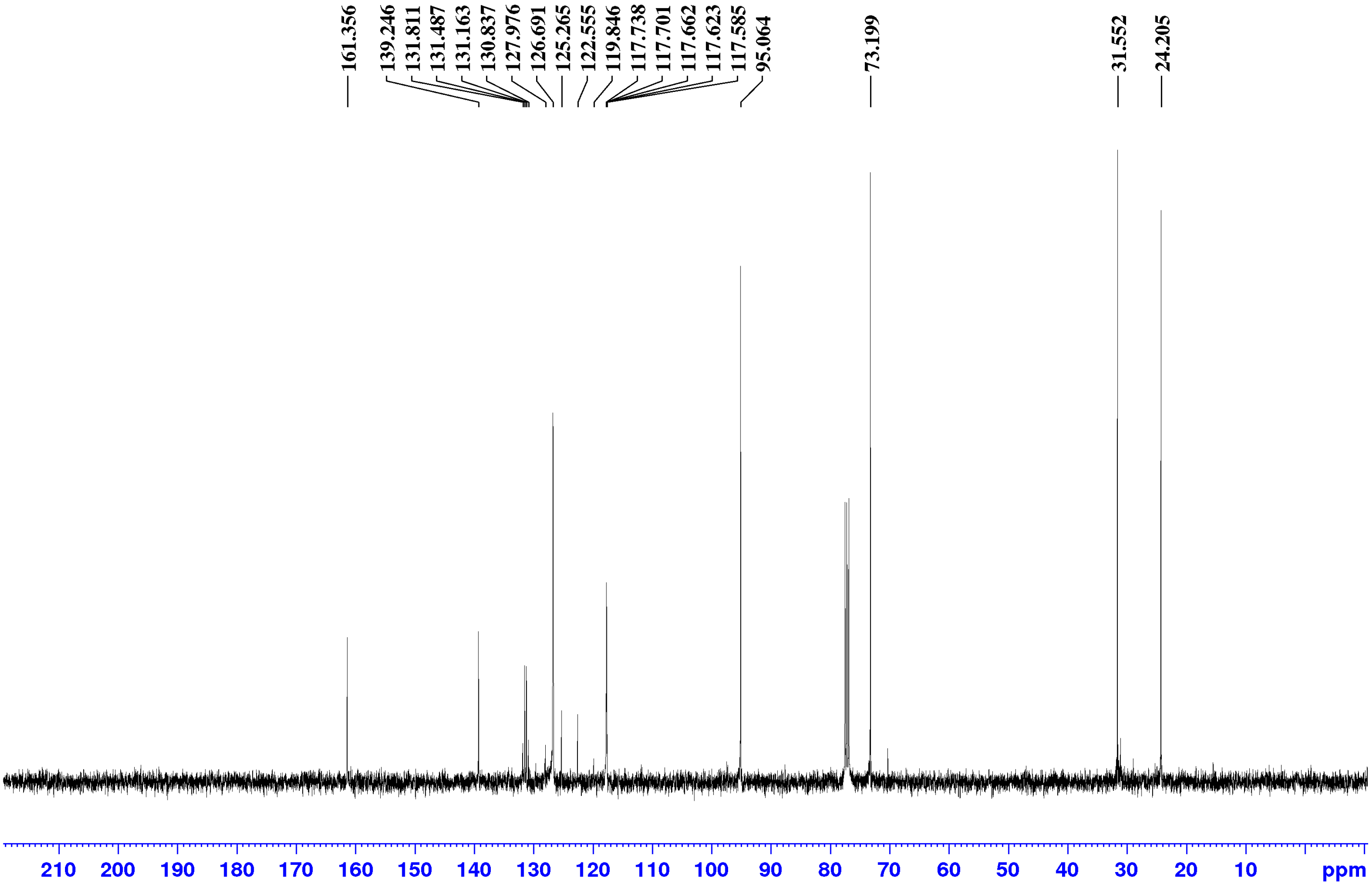

S36 - $^{13}C[^{1}H]$ spectrum of (Z)-2-(3,5-(bistrifluoromethyl)benzylidene)tetrahydrofuran in $CDCl_3$

(21) 4-(5-hydroxypent-1-yn-1-yl)benzonitrile

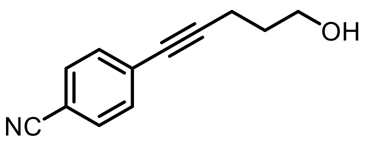

Yellow powdery solid
mp. : 76-79°C / R_f : (Hx:EtOAc 2:1) 0.15, (1:1) 0.30

NMR: **^{1}H** (400.1 MHz, $CDCl_3$) δ 7.56 (2H, dt, J = 8.5, 1.9 Hz, *Ar*), 7.44 (2H, dt, J = 8.4, 1.9 Hz, *Ar*), 3.80 (2H, t, J = 6.2 Hz, C*H₂*OH), 2.56 (2H, t, J = 7.0 Hz, CCC*H₂*), 1.86 (3H, quin, J = 6.6 Hz, C*H₂*CH₂OH), 1.65 (1H, s, O*H*). **^{13}C{^{1}H}** (100.7 MHz, $CDCl_3$) δ 132.2 (Ar), 132.1 (Ar), 129.0 (AR_{ipso}), 118.7 (*C*N), 111.1 (Ar_{ipso}), 94.7 (ArC*C*), 79.9 (Ar*C*C), 61.6 (*C*H_2OH), 31.3 (CH_2*C*H_2OH), 16.2 (*C*H_2CH_2OH).

MS (NSI-FTMS): 209.0766 $(M+Na)^+$ – $C_{17}H_{11}ONNa$

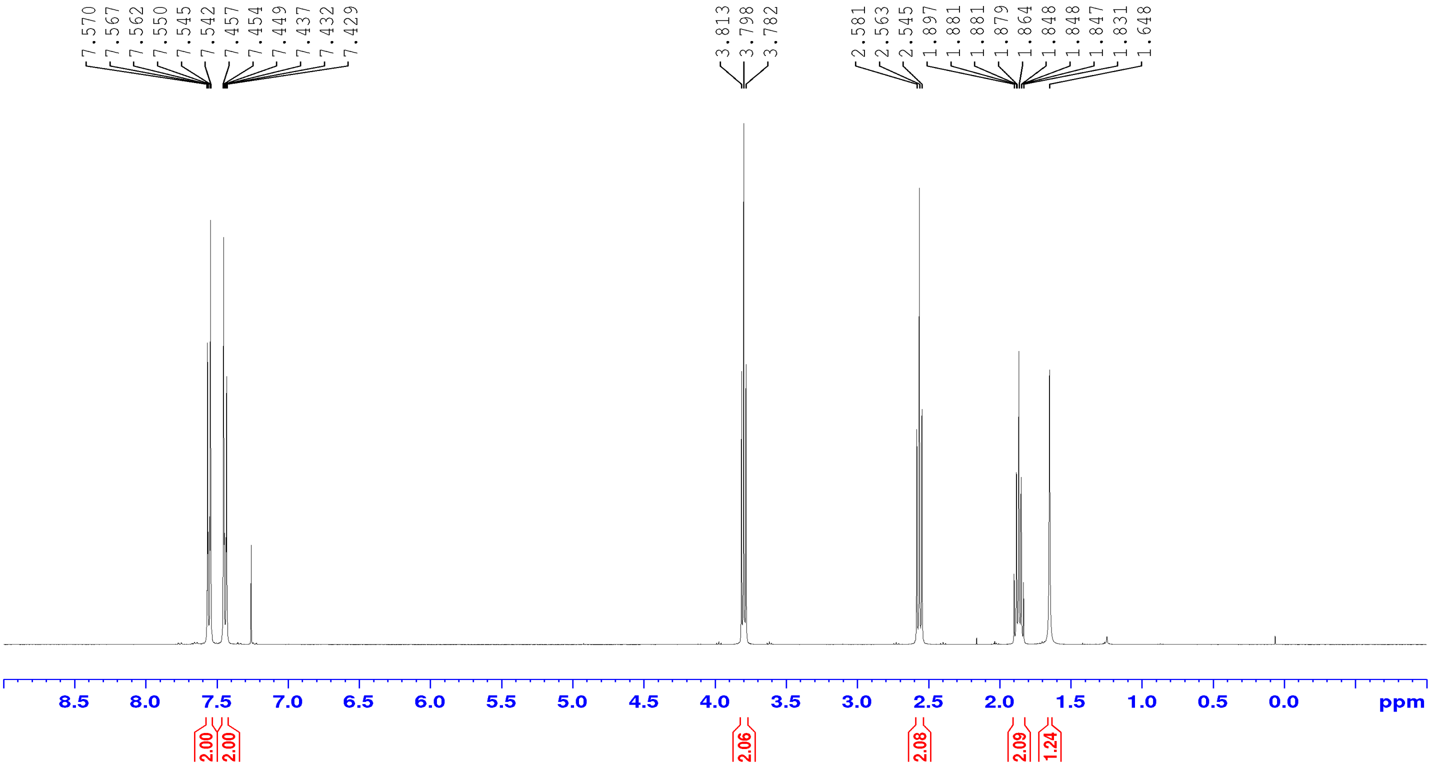

S39 – ^{1}H spectrum of 4-(5-hydroxypent-1-yn-1-yl)benzonitrile in $CDCl_3$

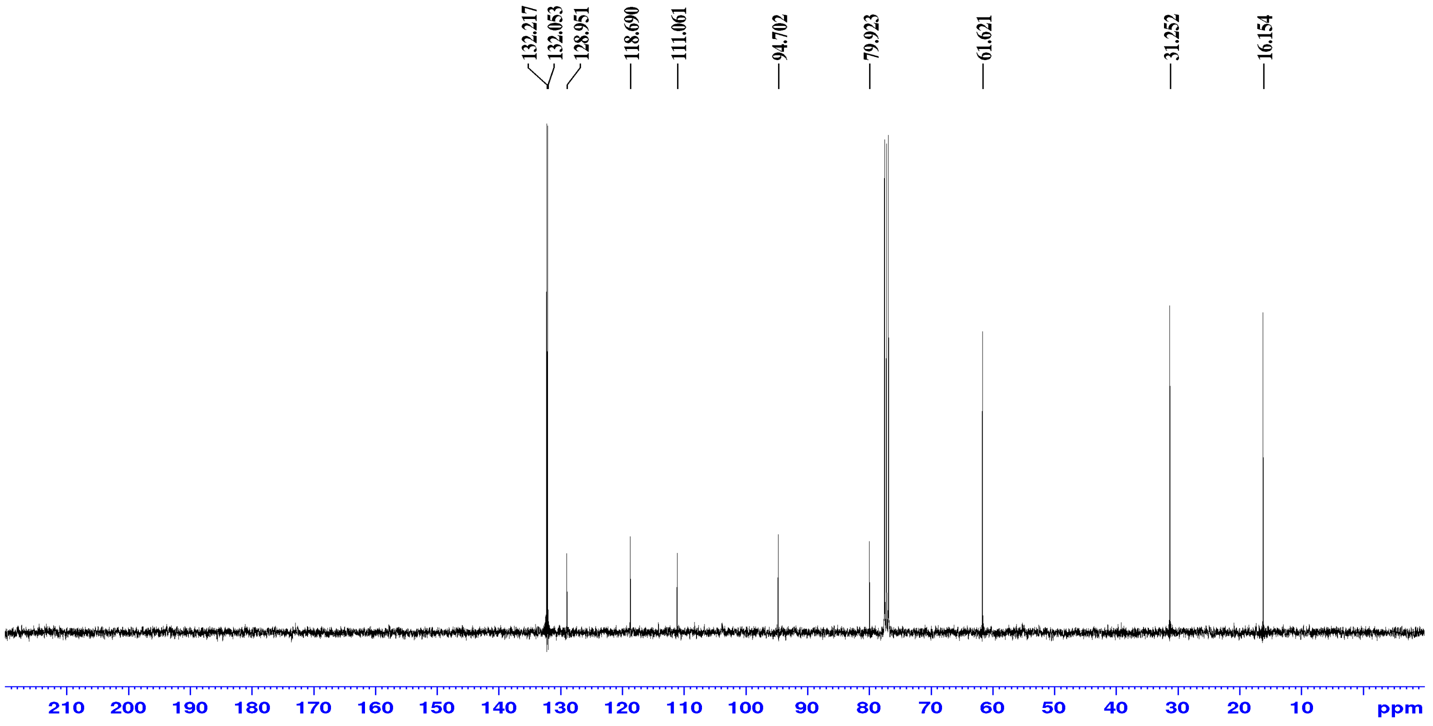

S38 – ^{13}C[^{1}H} spectrum of 4-(5-hydroxypent-1-yn-1-yl)benzonitrile in $CDCl_3$

(22a) (Z)-4-((dihydrofuran-2(3H)-ylidene)methyl)benzonitrile

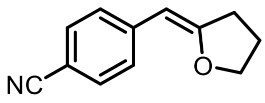

Colourless crystalline solid
mp. : 109-111°C / R_f : (Hx:EtOAc 2:1) 0.50, (1:1) 0.69

NMR: **^{1}H** (400.1 MHz, $CDCl_3$) δ 7.56 (2H, d, J = 8.4 Hz, *Ar*), 7.48 (2H, d, J = 8.4 Hz, *Ar*), 5.24), 5.20 (1H, s, CC*H*), 4.36 (2H, t, J = 6.8 Hz, C*H*$_2$O), 2.74 (2H, td, J = 7.6, 0.9 Hz, CC*H*$_2$), 2.04 (2H, quin, J = 7.2 Hz, OCH$_2$C*H*$_2$). **^{13}C{^{1}H}** (100.7 MHz, $CDCl_3$) δ 161.6 (O*C*CH), 142.0 (Ar$_{ipso}$), 131.93 (Ar), 127.2 (Ar), 119.9 (*C*N), 106.8 (Ar$_{ipso}$), 96.0 (C*C*H), 73.1 (O*C*H$_2$), 31.6 (OCH$_2$*C*H$_2$), 24.0 (CC*C*H$_2$).
MS (ASAP/TOF-MS): 186.0919 (M+H) - $C_{12}H_{12}NO$

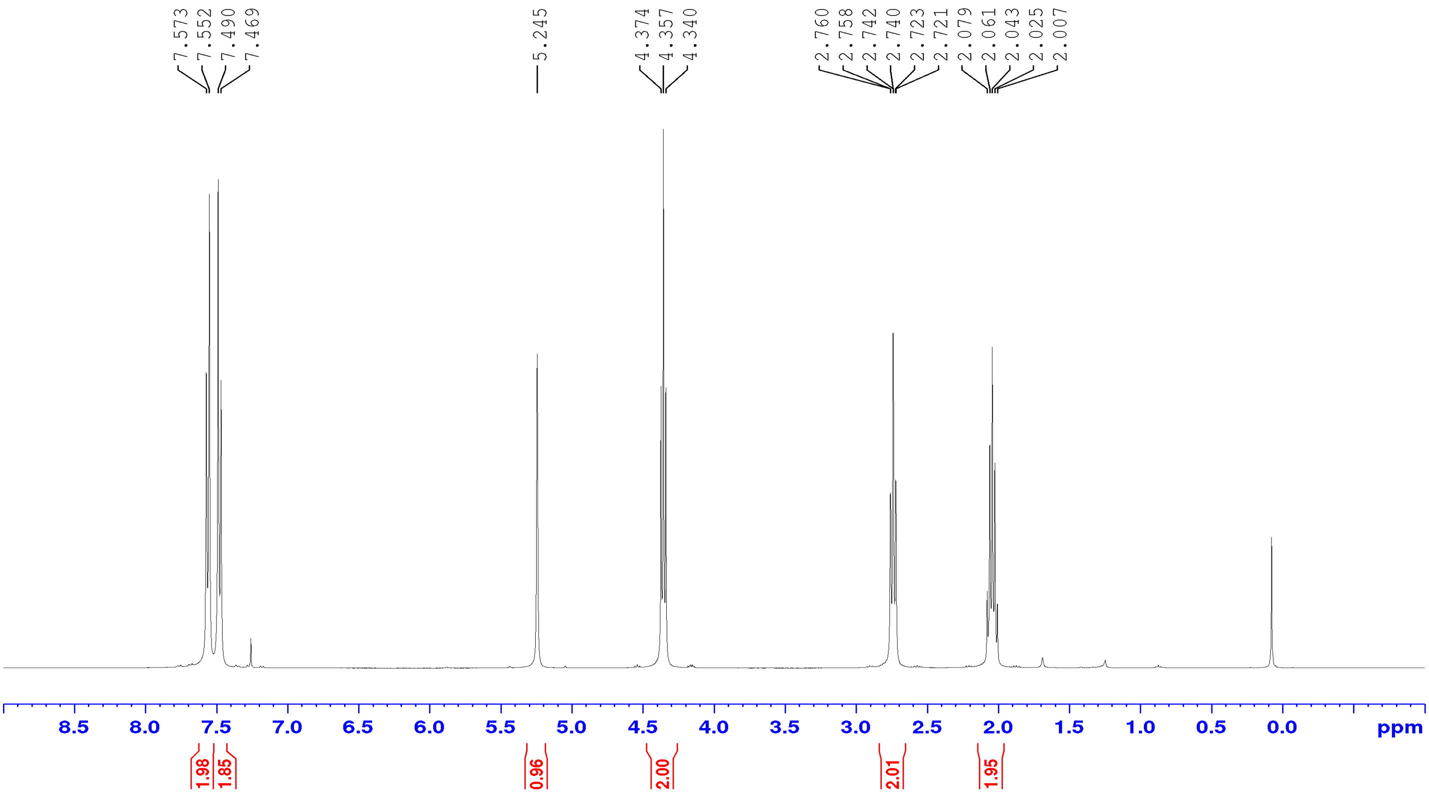

S41 - ^{1}H spectrum of (Z)-4-((dihydrofuran-2(3H)-ylidene)methyl)benzonitrile in $CDCl_3$

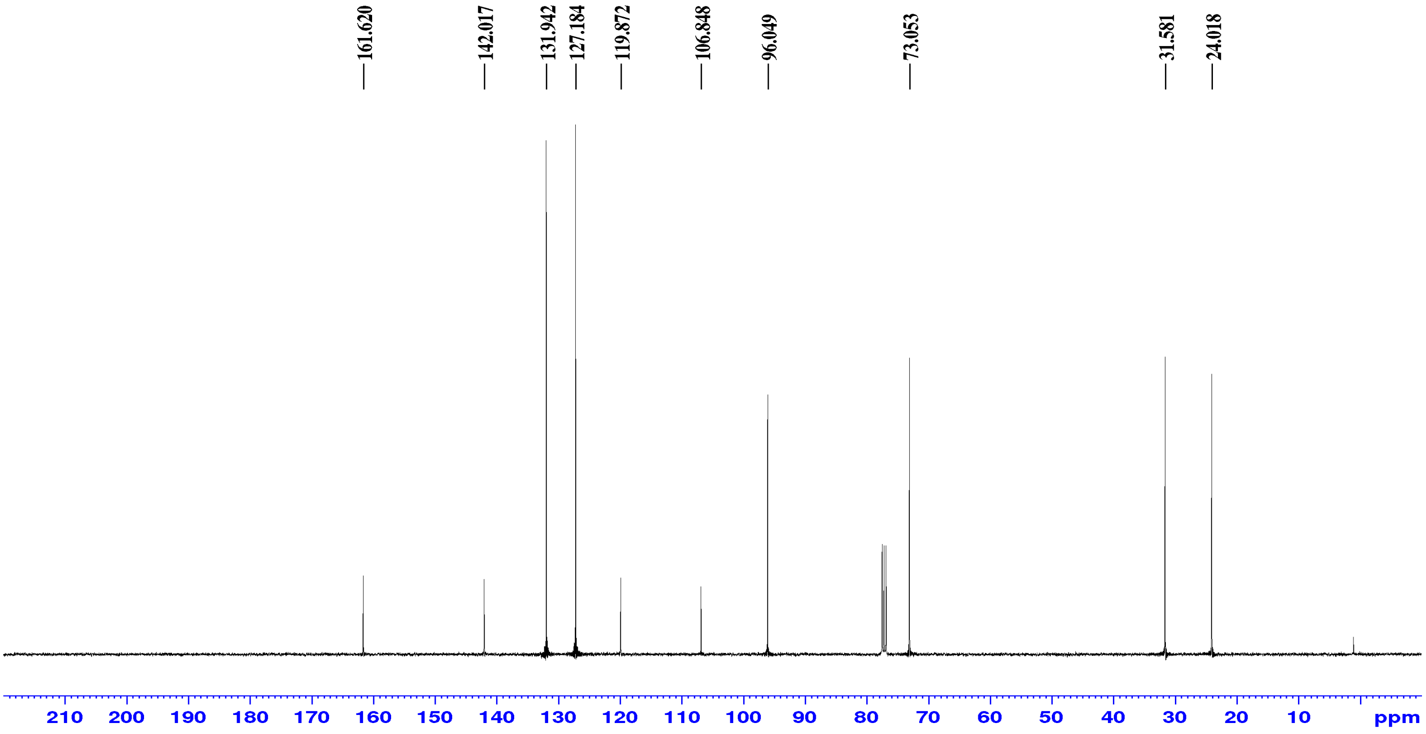

S40 – ^{13}C[^{1}H] spectrum of (Z)-4-((dihydrofuran-2(3H)-ylidene)methyl)benzonitrile in $CDCl_3$

(23) 5-(4-(tert-butyl)phenyl)pent-4-yn-1-ol

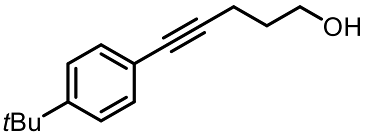

R_f: (Hx:EtOAc 2:1) 0.33, (1:1) 0.55

NMR: **^{1}H** (400.1 MHz, $CDCl_3$) δ 7.33 (2H, d, J = 8.7 Hz, *Ar*), 7.30 (2H, d, J = 8.7 Hz, *Ar*), 3.82 (2H, m, CH_2OH), 2.53 (3H, t, J = 6.9 Hz, CCCH_2), 1.86 (2H, quin, J = 6.5 Hz, CH_2CH_2OH), 1.63 (1H, s, O*H*), 1.30 (9H, s, *t*Bu). **^{13}C{^{1}H}** (100.7 MHz, $CDCl_3$) δ 150.9 (Ar_{ipso}), 131.4 (Ar), 125.4 (Ar), 120.8 (Ar_{ipso}), 88.6 (Ar*C*C), 81.3 (ArC*C*), 62.0 (CH_2OH), 34.8 (*C*$[CH_3]_3$), 31.6 (CH_2CH_2OH), 31.3 (C$[CH_3]_3$), 16.2 (CCCH_2).

MS (ASAP/TOF-MS): 217.1592 (M+H) - $C_{15}H_{21}O$

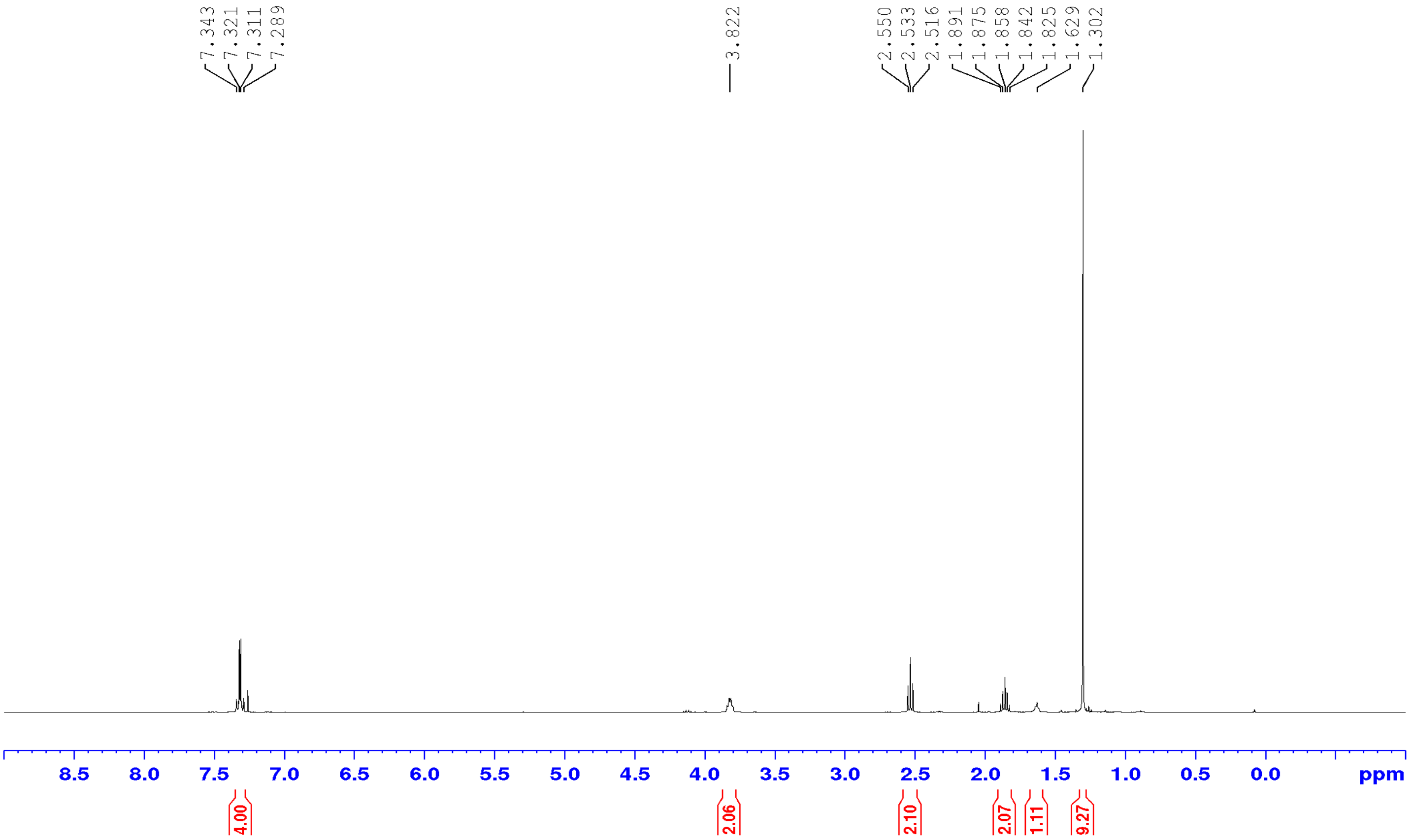

S43 – ^{1}H spectrum of 5-(4-(tert-butyl)phenyl)pent-4-yn-1-ol in $CDCl_3$

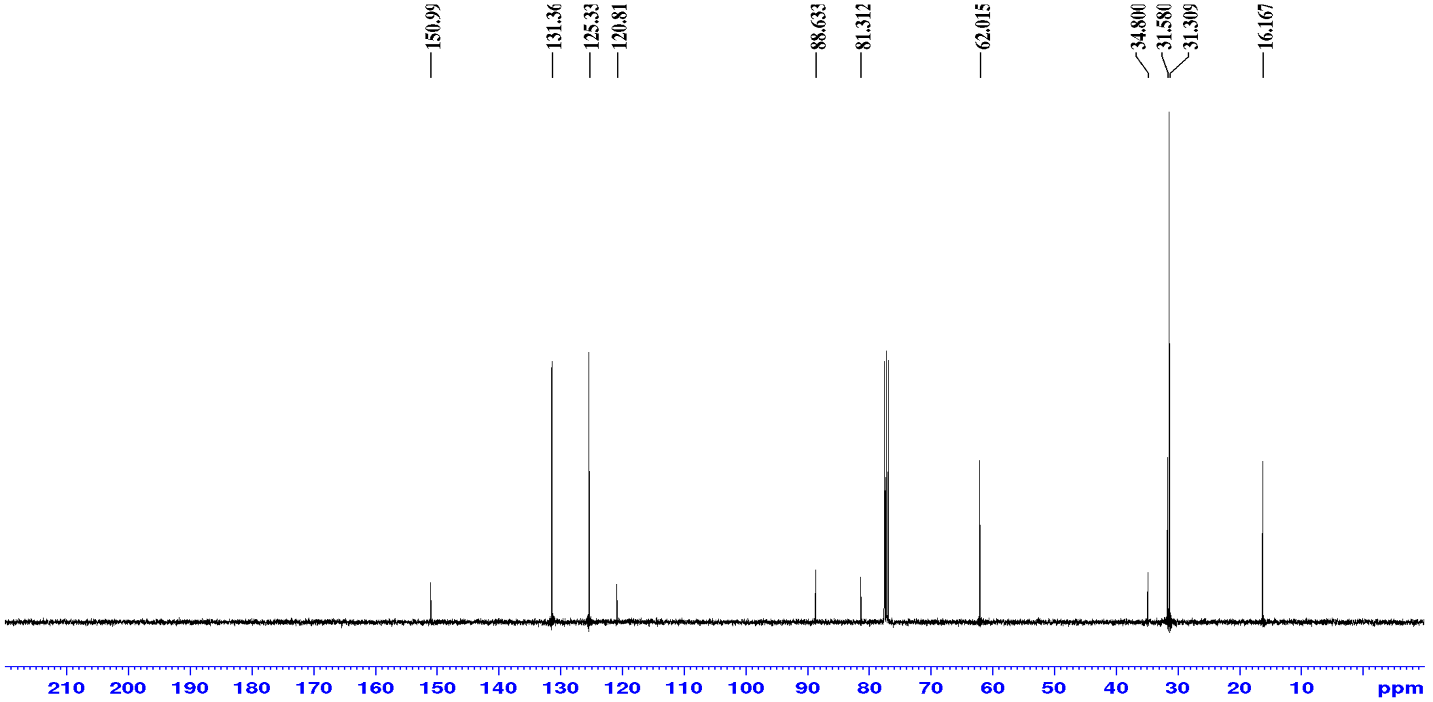

S42 – ^{13}C[^{1}H} spectrum of 5-(4-(tert-butyl)phenyl)pent-4-yn-1-ol in $CDCl_3$

(24a) (Z)-2-(2,2-dimethylpropylidene)tetrahydrofuran

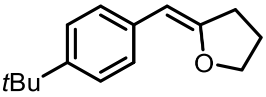

R_f : (Hx:EtOAc 2:1) 0.59, (1:1) 0.68

NMR: **^{1}H** (400.1 MHz, $CDCl_3$) δ 7.31 (2H, dt, J = 8.4, 2.0 Hz, *Ar*), 7.13 (2H, dt, J = 8.4, 2.0 Hz, *Ar*),5.90 (1H, s, CC*H*), 4.11 (2H, t, J = 6.8 Hz, C*H*$_2$O), 2.82 (2H, dt, J = 7.6, 2.0 Hz, CC*H*$_2$), 2.0 (2H, quin, J = 7.0 Hz, OCH_2C*H*$_2$), 1.32 (9H, s, *t*Bu). **^{13}C{^{1}H}** (100.7 MHz, $CDCl_3$) δ 158.7 (O*C*CH), 147.5 (Ar_{ipso}), 134.9 (Ar_{ipso}), 126.7 (Ar), 125.3 (Ar), 98.8 (*C*CH), 69.5 (O*C*H_2), 34.5 (*C*[CH_3]$_3$), 31.5 (C[*C*H_3]$_3$), 28.3 (OCH_2*C*H_2), 25.5 (CC*C*H_2).

MS (ASAP/TOF-MS): 216.1514 (M) - $C_{15}H_{20}O$

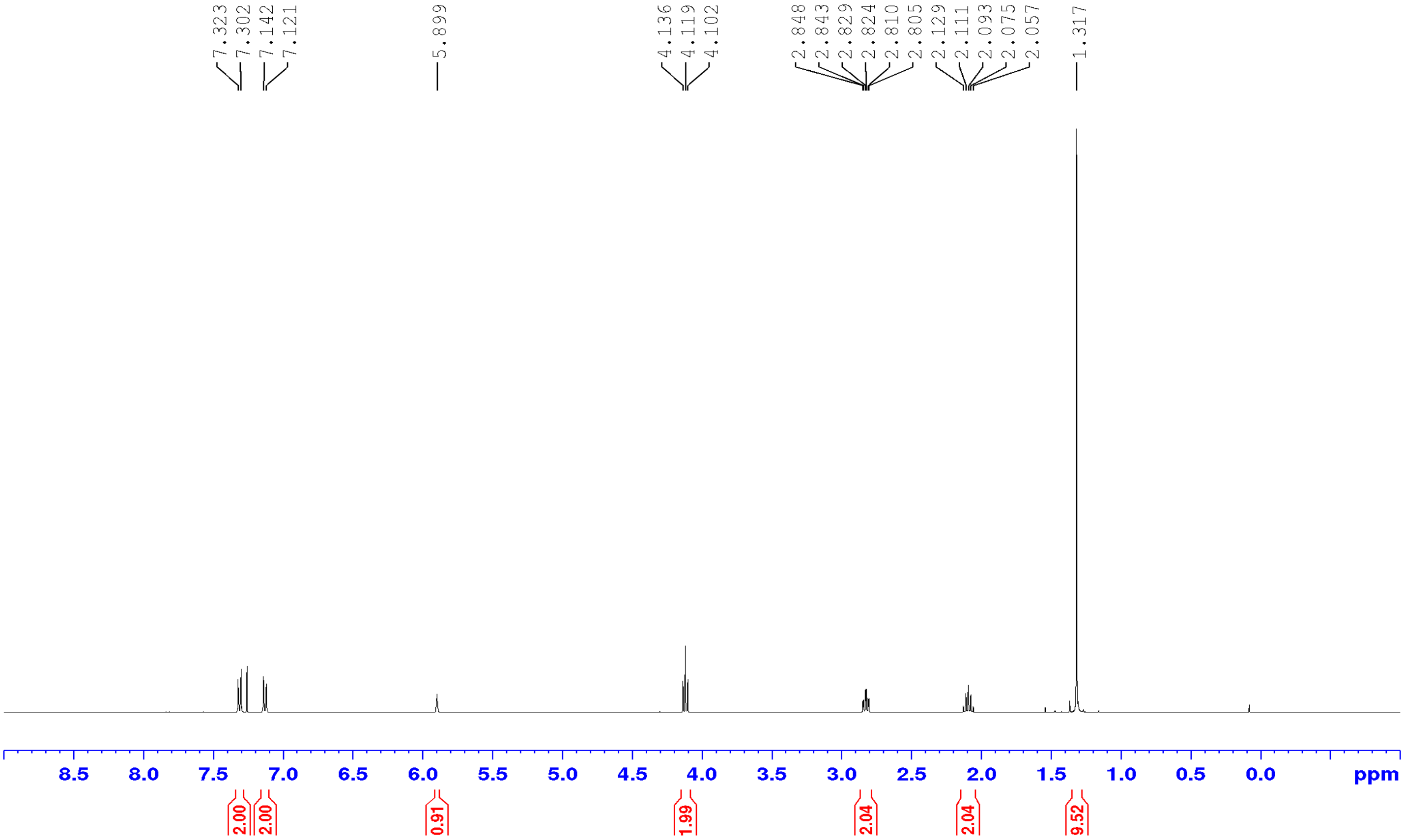

S45 – ^{1}H spectrum of (Z)-2-(2,2-dimethylpropylidene)tetrahydrofuran in $CDCl_3$

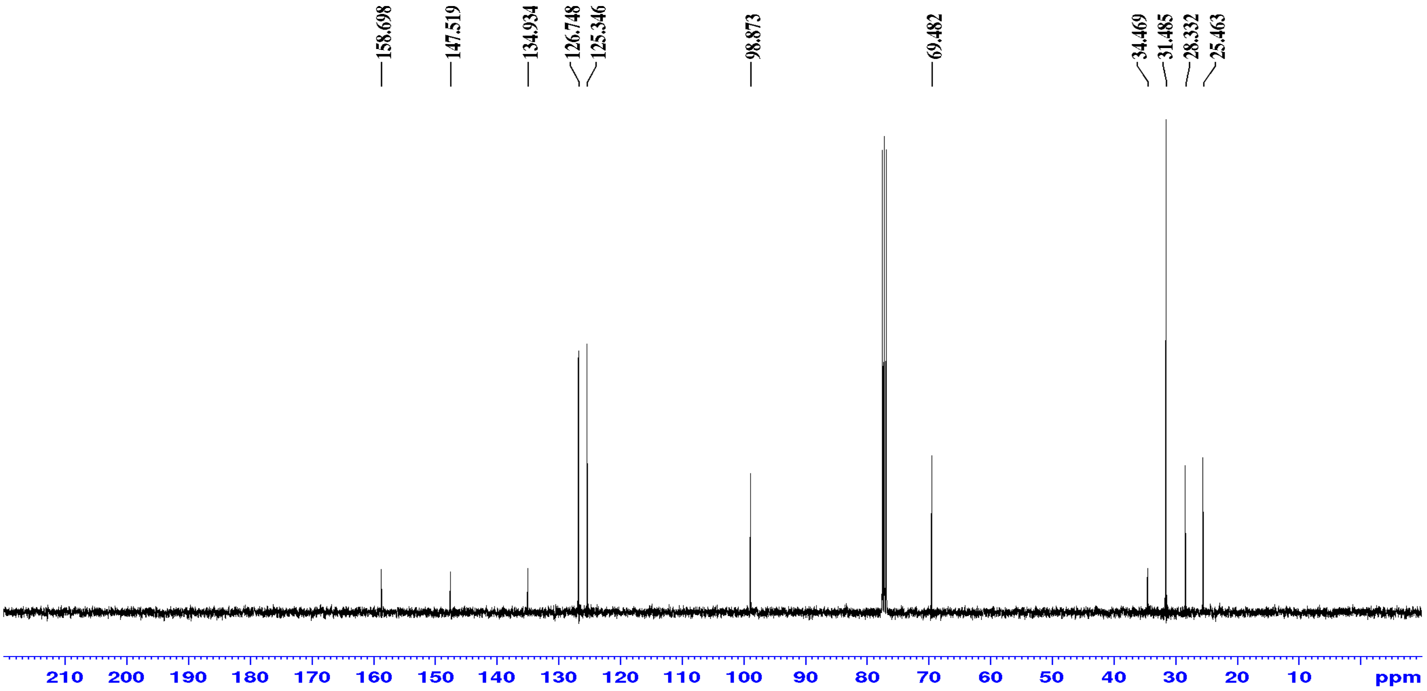

S44 – ^{13}C[^{1}H} spectrum of (Z)-2-(2,2-dimethylpropylidene)tetrahydrofuran in $CDCl_3$

References

S1 (a) E. Hevia, *Chem. Commun.*, 2011, **47**, 388; (b) J. Klett, *Eur. J. Inorg. Chem.*, 2011, 721 (c) E. Hevia, *Organometallics*, 2012, **31**, 5131; (d) E. Hevia, *Chem. Commun.*, 2014, **50**, 12859; (e) E. Hevia, *Dalton Trans.*, 2016, **45**, 6175.

S2 N. T. Coles, M. F. Mahon and R. L. Webster, *Chem. Commun.*, 2018, **54**, 10443-10446.

S3 (a) T. J. Marks, *J. Am. Chem. Soc.*, 2009, **131**, 263; (b) J. F. Sebastian, *J. Org. Chem.*, 1980, **45**, 4959.

S4 M. J. Coster, *Tetrahdron lett.*, 2001, **52**, 1070.

S5 T. J. Marks, *J. Am. Chem. Soc.*, 2007, **129**, 7244.

S6 (a) P. Pale, *Eur. J. Inorg, Chem.*, 2000, **6**, 1019; (b) J. García-Álvarez*, Green Chem*., 2015, **17**, 3870.

S7 A. G. M. Barrett, M. S. Hill, *Organometallics*, 2012, **31**, 7287.

S8 (a) S. Stahl, *J. Am. Chem. Soc.*, 2007, **129**, 6328 ;(b) X. P. Zang, *Angew. Chem. Int. Ed.*, 2014, **53**, 7028.
